# Sociodemographic Heterogeneity in the Associations of Social Isolation With Mortality

**DOI:** 10.1001/jamanetworkopen.2024.13132

**Published:** 2024-05-24

**Authors:** Atsushi Nakagomi, Masashige Saito, Toshiyuki Ojima, Takayuki Ueno, Masamichi Hanazato, Katsunori Kondo

**Affiliations:** 1 Department of Social Preventive Medical Sciences, Center for Preventive Medical Sciences, Chiba University, Chiba, Japan; 2 Faculty of Social Welfare, Nihon Fukushi University, Aichi, Japan; 3 Center for Well-being and Society, Nihon Fukushi University, Aichi, Japan; 4 Department of Community Health and Preventive Medicine, Hamamatsu University School of Medicine, Shizuoka, Japan; 5 Department of Environmental Preventive Medical Sciences, Center for Preventive Medical Sciences, Chiba University, Chiba, Japan; 6 Center for Gerontology and Social Science, Research Institute, National Center for Geriatrics and Gerontology, Aichi, Japan

## Abstract

**Importance:**

There are limited data on whether the vulnerabilities and impacts of social isolation vary across populations.

**Objective:**

To explore the association between social isolation and mortality due to all causes, cardiovascular diseases (CVD), and malignant neoplasms focusing on heterogeneity by sociodemographic factors.

**Design, Setting, and Participants:**

This cohort study used a moderator-wide approach to examine the heterogeneity in the association of social isolation with all-cause, CVD, and malignant neoplasm mortality using baseline data from the Japan Gerontological Evaluation Study in 2010 and 2011. Eligible participants were adults aged 65 years or older without heart disease, stroke, cancer, or impaired activity of daily living across 12 Japanese municipalities. Follow-up continued until December 31, 2017, identifying 6-year all-cause, cardiovascular disease (CVD), and malignant neoplasm mortality. Logistic regression assessed effect modification by age, gender, education, income, population density, marital status, and employment on mortality associations. Data analysis was performed from September 13, 2023, to March 17, 2024.

**Exposure:**

Social isolation, determined by a 3-item scale (scores of 2 or 3 indicating isolation) was the primary exposure variable.

**Main Outcomes and Measures:**

Six-year all-cause, CVD, and malignant neoplasms mortality.

**Results:**

This study included 37 604 older adults, with a mean (SD) age of 73.5 (5.9) years (21 073 women [56.0%]). A total of 10 094 participants (26.8%) were classified as experiencing social isolation. Social isolation was associated with increased all-cause (odds ratio [OR], 1.20 [95% CI, 1.09-1.32]), CVD (OR, 1.22 [95% CI, 0.98-1.52]), and malignant neoplasm mortality (OR, 1.14 [95% CI, 1.01-1.28]). Stratified analysis showed associations of social isolation with all-cause and malignant neoplasm mortality among people with high income (highest tertile all cause: OR, 1.27 [95% CI, 1.06-1.53]; malignant neoplasm: OR, 1.27 [95% CI, 1.02-1.60]), living in areas with high population density (highest tertile all cause: OR, 1.47 [95% CI, 1.26-1.72]; malignant neoplasm: OR, 1.38 [95% CI, 1.11-1.70]), not married (all cause: OR, 1.33 [95% CI, 1.15-1.53]; malignant neoplasm: OR, 1.25 [95% CI, 1.02-1.52]), and retirees (all cause: OR, 1.27 [95% CI, 1.14-1.43]; malignant neoplasm: OR, 1.27 [95% CI, 1.10-1.48]). Formal testing for effect modification indicated modification by population density and employment for all-cause mortality and by household income and employment for neoplasm mortality.

**Conclusions and Relevance:**

Social isolation was associated with increased risks of all-cause, CVD, and malignant neoplasm mortality, with associations varying across populations. This study fills an important gap in research on social isolation, emphasizing its varied associations across demographic and socioeconomic groups.

## Key Points

**Question:**

Does social isolation increase mortality due to all causes, cardiovascular diseases, and malignant neoplasms differently across sociodemographic strata?

**Findings:**

In this cohort study of 37 604 participants, a moderator-wide approach illustrated diverse associations between social isolation and mortality based on income, population density, marital status, and employment status. Formal assessments for effect modification found heterogeneity by population density and employment for all-cause mortality and by income and employment for malignant neoplasm mortality.

**Meaning:**

These results suggest that tailoring interventions to target specific sociodemographic groups could enhance the effectiveness of efforts aimed at mitigating the mortality risks associated with social isolation.

## Teaser

This cohort study of older Japanese adults examines how income, population density, marital, and employment status may affect the association between social isolation and mortality from all causes, cardiovascular disease, and malignant neoplasms.

## Introduction

Social isolation, the objective lack of social relationships or contact,^1^ is consistently associated with greater all-cause mortality in the general population.^2,3,4^ Emerging research also indicates a connection with mortality due to cardiovascular diseases (CVD) and malignant neoplasms.^5^ Recognizing this, the American Heart Association (AHA) has recently acknowledged social isolation as a likely independent risk factor for adverse cardiovascular and brain health outcomes.^6^

The possible mechanisms through which social isolation affect mortality include health behaviors, psychological, and physiological factors.^1,6^ For example, social isolation may increase CVD and malignant neoplasms mortality via harmful behaviors (eg, smoking), psychological stress (eg, depression^7^), and physiological changes (eg, activation of the hypothalamic-pituitary-adrenal axis^8^). In addition, less access to emergency and routine medical care due to a small social network may increase the risk of mortality.^9^

The effects of social isolation on mortality risk are well established, as are the plausible mechanisms that explain the effects. This has led to a growing interest in moderating factors that may influence the strength or direction of these effects.^1^ For example, social isolation may increase smoking, but the impacts may vary by gender.^10^ The National Academies of Sciences, Engineering, and Medicine’s Consensus Study Report identified age, gender, and socioeconomic status (SES) as potential moderating factors, although the evidence base is not robust.^1^ Age may alter the relationship between social isolation and all-cause mortality, particularly affecting younger adults more severely.^11,12^ Yet, limited evidence exists on age moderation in the impact of social isolation on CVD and neoplasm mortality. SES—encompassing education, income, and residence—is another potential key modifier. However, most studies have not explored whether the impact of social isolation on mortality varies by SES. A 2021 study demonstrated that the magnitude of mortality risks associated with social isolation was greatest in high-income countries, but it remains unclear if there is heterogeneity by individual income levels.^12^ This study also examined heterogeneity by residential area (urban vs rural), finding no statistically significant interaction. Nevertheless, the estimate of social isolation for all-cause mortality was higher in urban areas compared with rural areas, prompting further investigation. Marital and employment status may also modify the impact of social isolation on mortality, with potentially greater effects on unmarried or unemployed individuals.^13^ The extent to which these demographic and social factors moderate the impact of social isolation on mortality due to CVD and malignant neoplasms is not fully understood. The AHA highlights the need for research in these areas, especially among socially vulnerable groups prone to social isolation,^6^ due to lack of comprehensive data on these potential moderating factors.

This study explores the role of demographic and social factors as potential moderators, using a moderator-wide approach to assess if these factors affected associations between social isolation and mortality due to all-cause, CVD, and malignant neoplasms.^14^ The study analyzed how age, gender, education, income, population density, marital status, and employment status might moderate the link between social isolation and mortality in older adults. This method, hypothesis-generating and data-driven, seeks to identify which factors may affect these associations, potentially guiding future research. The goal is to understand the uneven effects of social isolation and pinpoint groups needing prioritized intervention.

## Methods

### Study Sample

Baseline data were collected using a self-administered questionnaire from August 2010 to December 2011 as part of the Japan Gerontological Evaluation Study (JAGES). Data were obtained from 46 616 older individuals in 12 municipalities of Japan aged 65 years or older, physically and cognitively independent, and residing independently in the community (response rate, 64.7%). These municipalities, representing both urban and rural areas in Japan, were diverse in regional representation and population size. The questionnaire was deployed via complete enumeration in 9 smaller municipalities, whereas a randomized sampling methodology was employed in 3 municipalities. Information on the date of death was sourced from the public long-term care insurance system database administered by the municipal governments. Subsequently, the study used vital statistics data to discern the causes of participants’ deaths. In the data integration process, gender, date of birth, date of death, and municipality name were key variables, including 46 144 respondents in the subsequent analysis (follow-up rate, 98.9%). Our questionnaire did not include race, as the majority of our study's participants were presumed to be Japanese. They were followed up until December 31, 2017, for cause of death using the vital national statistics. We excluded participants without data on population density (79 participants) and those who indicated stroke, heart disease, cancer, and impaired activities of daily living in their self-reported questionnaire (8461 participants).

The ethics committee of Chiba University granted ethical clearance. JAGES participants were informed of their voluntary participation, with returned questionnaires implying consent. Data anonymization was ensured, and all procedures complied with relevant guidelines and the Declaration of Helsinki.^15^ The study followed the Strengthening the Reporting of Observational Studies in Epidemiology (STROBE) reporting guideline.

### Measures

#### Six-Year All-Cause Mortality, CVD Mortality, and Malignant Neoplasm Mortality

We defined CVD mortality as death from acute myocardial infarction, other ischemic heart diseases, cerebrovascular disease, subarachnoid hemorrhage, intracerebral hemorrhage, cerebral infarction, and other cerebrovascular diseases. Malignant neoplasm mortality was defined as death from any malignant neoplasms. To maintain a consistent follow-up duration across all participants, we excluded follow-up periods exceeding 6 years to match the minimum follow-up period of municipalities.

### Social Isolation

Our exposure variable was measured in the baseline wave (2010). The social isolation scale comprises three measures: (1) living alone, (2) having less than monthly contact with friends, and (3) nonparticipation in social activities (sports, hobbies, or volunteering) weekly. Our scale ranges from 0 to 3. We classified the scale into social isolation (social isolation scale score of 2 or higher) and nonsocial isolation (isolation scale score below 2). This aligns with studies utilizing the UK Biobank, where the social scale was determined by assigning 1 point for living alone, 1 point for less than monthly visits from friends and family, and 1 point for nonparticipation in social activities every week.^16^

### Covariates

Our covariates were measured in the baseline wave. As confounders and potential moderators, we included age, gender, socioeconomic status (education, household equivalized income, and population density), marital status, and employment. As confounders and potential mediators, we included behavioral factors (smoking, drinking alcohol, walking, eating meat and fish, eating fruit and vegetables, and attending health check-ups), psychological factors (depressive symptoms), and physiological factors (body mass index [calculated as weight in kilograms divided by height in meters squared], self-rated health, self-reported hypertension, self-reported diabetes, and self-reported dyslipidemia). Details of the definitions for covariates are described in the eData in the Supplement 1.

### Statistical Analysis

We used logistic regression to calculate odds ratios (ORs) of social isolation for mortality during a 6-year follow-up, as outcomes (all-cause, CVD, and neoplasm mortality) were rare (under 10%). We adjusted for all covariates because we could not identify the temporal order of exposures and covariates. This is described in detail in eAppendix 1 in Supplement 1 (determining adjusting variables). Standard errors were clustered at the school district level to account for the potential correlation of the participants within the same districts. We used school district because a school district reflects a geographical scale wherein older Japanese people can move on foot or by bike and a unit of community organization.^17^

We used a moderator-wide approach to examine the heterogeneity in the association of social isolation with all-cause, CVD, and neoplasm mortality.^14^ We introduced product terms between social isolation and each moderator of interest (age, gender, education, household income, population density, marital status, and employment status). We also calculated the ORs within each moderator stratum. For the stratified analysis, age was categorized into 75 years or older and younger than 75 years, while household equivalized income and population density were divided into tertiles. We conducted formal assessments for effect modification on both additive and multiplicative scales. We measured the relative excess risk due to interaction (RERI) with a 95% CI obtained by the delta method^18^ and the ratio of odds ratios (ROR) based on the fully adjusted logistic models.^19^ The additive scale of effect modification assesses changes in absolute risk differences across effect modifier strata. This approach elucidates the potential variability in the absolute reduction of mortality attributable to modifications in exposure levels (eg, social isolation) across diverse social groups. While substantial multiplicative effect modification can exist, if the prevalence of the outcome is low, the population impact of effect heterogeneity may be minimal. Despite its direct relevance for policymakers and public health officials, the additive effect modification scale has rarely been reported.^19^ We also conducted 5 sensitivity analyses. The details are described in eAppendix 1 in Supplement 1.

All the analyses were conducted using the R software version 4.3.2 (R Foundation for Statistical Computing) and Stata 18.0 software (Stata Corp LLC) (eAppendix 2 in Supplement 1). Missing data were imputed using a random forest approach with missForest package.^20^ The number and percentage of missing values are presented in eTable 1 in Supplement 1. Two-tailed *P* < .05 was considered statistically significant. To account for multiple testing, we applied the Benjamini-Hochberg procedure to calculate correct *P* values for estimates of effect modification.^21^

## Results

### Descriptive Statistics

A total of 37 604 participants were included (mean [SD] age, 73.5 [5.9] years; 21 073 women [56.0%]), 20 050 (53.3%) with an education of 10 years or more, 10 601 (28.2%) unmarried, and 24 096 (64.1%) retired (Table; eTable 2 in the Supplement 1). The mean (SD) household equivalized income and population density were ¥234.0 (145.6) million and 4231.8 (3946.2) persons/km^2^, respectively. A total of 10 094 (26.8%) were classified as experiencing social isolation. Those who were socially isolated were typically older (mean [SD] age, 74.2 [6.5] years vs 73.2 [5.7] years), more often male (5248 of 10 094 [52.0%] vs 11 283 of 27 510 [41.0%]), had lower educational levels (below 10 years education: 5107 of 10 094 [50.6%] vs 12 447 of 27 510 [45.2%]) and household incomes (mean [SD] annual income, ¥214.4 [137.0] million vs ¥241.1 [148.0] million) (the conversion from Japanese yen to US dollars based on the exchange rate as of May 1, 2024, was $1 = ¥157.36), were less likely to be married (6017 of 10 094 [59.6%] vs 20 986 of 27 510 [72.3%]), engaged in fewer positive healthy behaviors (eg, participants with current smoking habits: 1616 of 10 094 [16.0%] vs 2643 of 27 510 [9.6%]), and reported poorer mental (Geriatric Depression Scale rating 5 or above: 4119 of 10 094 [40.8%] vs 5671 of 27 510 [20.6%]) and self-rated health (mean [SD] rating, 2.8 [0.6] vs 3.0 [0.6]). The counts for 6-year all-cause mortality, 6-year CVD mortality, and 6-year neoplasm mortality were 3568 (9.5%), 419 (1.1%), and 1463 (3.9%), respectively.

**Table.** Baseline Characteristics of Imputed Data From 2010 Survey

Baseline characteristics	Participants, No. (%)		
	Total (N = 37 604)	No social isolation (n = 27 510)	Social isolation (n = 10 094)
Age, mean (SD), y	73.5 (5.9)	73.2 (5.7)	74.2 (6.5)
Gender			
Women	21 073 (56.0)	16 227 (59.0)	4846 (48.0)
Men	16 531 (44.0)	11 283 (41.0)	5248 (52.0)
Socioeconomic status			
Education			
≥10 y	20 050 (53.3)	15 063 (54.8)	4987 (49.4)
<10 y	17 554 (46.7)	12 447 (45.2)	5107 (50.6)
Household equivalized income, mean (SD), million ¥^a^	234.0 (145.6)	241.1 (148.0)	214.4 (137.0)
Population density, mean (SD), persons/km^2^	4231.8 (3946.2)	4179.9 (3913.1)	4373.2 (4031.7)
Marital status			
Not married	10 601 (28.2)	6524 (23.7)	4077 (40.4)
Married	27 003 (71.8)	20 986 (72.3)	6017 (59.6)
Employment			
Current	8351 (22.2)	6070 (22.1)	2281 (22.6)
Retired	24 096 (64.1)	17 623 (64.1)	6473 (64.1)
Never	5157 (13.7)	3817 (13.9)	1340 (13.3)
Behavioral factors			
Smoking			
Never	23 297 (62.0)	17 857 (64.9)	5440 (53.9)
Quit	10 048 (26.7)	7010 (25.5)	3038 (30.1)
Current	4259 (11.3)	2643 (9.6)	1616 (16.0)
Drinking alcohol			
Never	13 758 (36.6)	10 084 (36.7)	3674 (36.4)
Quit	989 (2.6)	642 (2.3)	347 (3.4)
Current	22 857 (60.8)	16 784 (61.0)	6073 (60.2)
Walking			
<30 min/d	11 446 (30.4)	7680 (27.9)	3766 (37.3)
30-59 min/d	14 333 (38.1)	10 655 (38.7)	3678 (36.4)
60-89 min/d	6062 (16.1)	4738 (17.2)	1324 (13.1)
≥90 min/d	5763 (15.3)	4437 (16.1)	1326 (13.1)
Eating meat and fish every day	15 456 (41.1)	11 894 (43.2)	3562 (35.3)
Eating fruits and vegetables every day	29 984 (79.7)	22 631 (82.3)	7353 (72.8)
Last health check-up			
Never	6324 (16.8)	3885 (14.1)	2439 (24.2)
>4 y ago	3705 (9.9)	2531 (9.2)	1174 (11.6)
2-3 y ago	4345 (11.6)	3138 (11.4)	1207 (12.0)
<1 y	23 230 (61.8)	17 956 (65.3)	5274 (52.2)
Psychological factors			
Geriatric Depression Scale ≥5	9790 (26.0)	5671 (20.6)	4119 (40.8)
Physiological factors			
Body mass index^b^			
<18.5	2579 (6.9)	1652 (6.0)	927 (9.2)
18.5 to <25.0	27 305 (72.6)	20 148 (73.2)	7157 (70.9)
25.0 to <30.0	7015 (18.7)	5204 (18.9)	1811 (17.9)
≥30.0	705 (1.9)	506 (1.8)	199 (2.0)
Self-rated health, mean (SD)^c^	3.0 (0.6)	3.0 (0.6)	2.8 (0.6)
Self-reported hypertension	19 674 (52.3)	14 650 (53.3)	5024 (49.8)
Self-reported diabetes	4349 (11.6)	3090 (11.2)	1259 (12.5)
Self-reported dislipidemia	3823 (10.2)	2961 (10.8)	862 (8.5)

^a^ The conversion from Japanese yen to US dollars based on the exchange rate as of May 1, 2024, was $1 = ¥157.36.

^b^ Calculated as weight in kilograms divided by height in meters squared.

^c^ Four-point scale, with 1 indicating poor health and 4 excellent health.

### Social Isolation and All-Cause, CVD, Malignant Neoplasm Mortality

**Figure 1.**
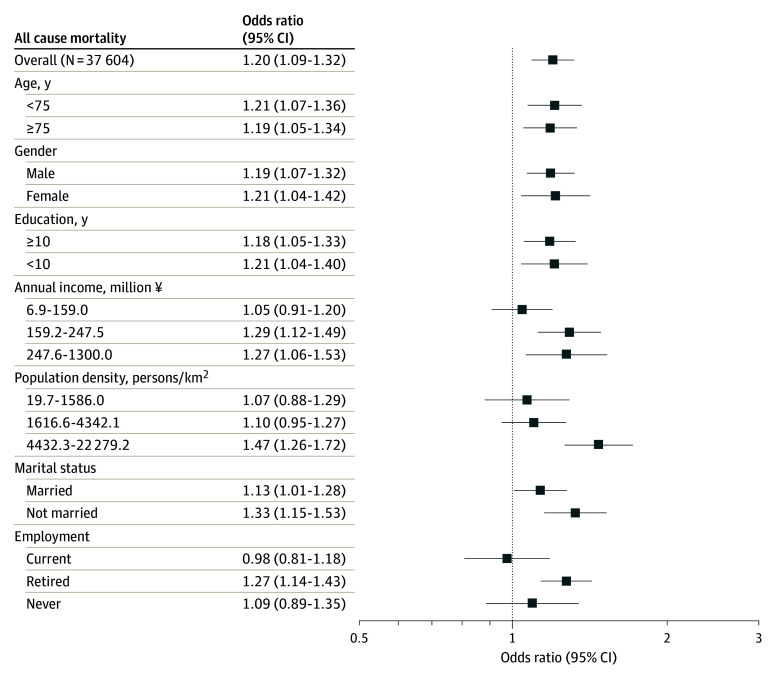
Odds Ratios of Social Isolation for All-Cause Mortality Within All Participants and Subgroups of Moderators The conversion from Japanese yen to US dollars based on the exchange rate as of May 1, 2024, was $1 = ¥157.36.

After adjustment for age and gender, socioeconomic status, marital status, employment, health behaviors, psychological factors, and physiological factors (fully adjusted model), the odds ratios (ORs) of social isolation were 1.20 (95% CI, 1.09-1.32) for all-cause mortality, 1.22 (95% CI, 0.98-1.52) for CVD mortality, and 1.14 (95% CI, 1.01-1.28) for malignant neoplasm mortality (eFigure 1 in Supplement 1). Figure 1 shows associations between social isolation and 6-year all-cause mortality risk stratified by moderators. Social isolation was associated with all-cause mortality consistently across age groups, in both men and women, and in both low and high education groups. Notable associations with all-cause mortality were found in some subgroups: the second and third household income tertiles (¥159.2 million to ¥247.5 million: OR, 1.29 [95% CI, 1.12-1.49]; ¥247.6 million to ¥1300 million: OR, 1.27 [95% CI, 1.06-1.53]); the highest population density tertile (4432.3 to 22 279.2 persons/km^2^: OR, 1.47 [95% CI, 1.26-1.72]); unmarried individuals (OR, 1.33 [95% CI, 1.15-1.53]); and retirees (OR, 1.27 [95% CI, 1.14-1.43]). eTable 3 in Supplement 1 details effect modification measures (RERIs and RORs), with evidence of modification by population density and employment, supporting the previously mentioned associations of subgroups.

**Figure 2.**
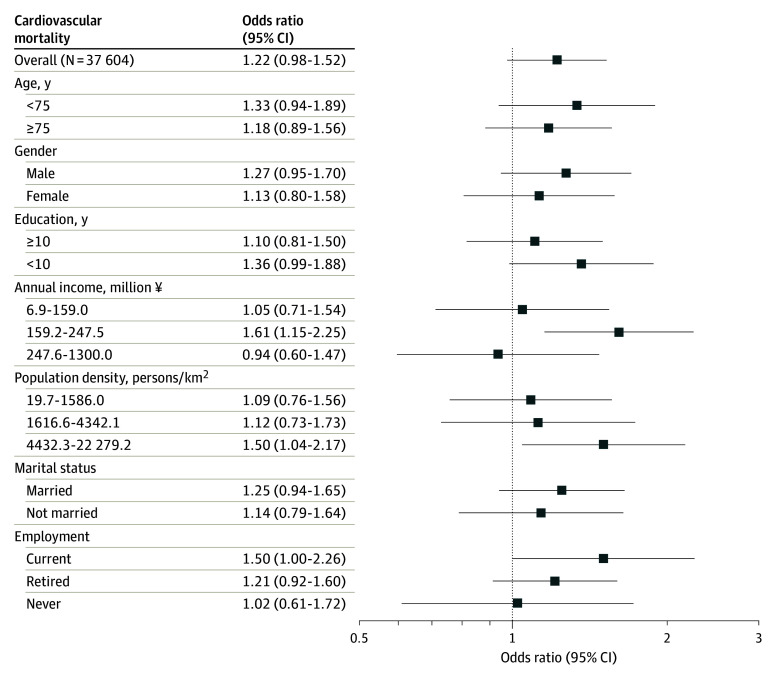
Odds Ratios of Social Isolation for Cardiovascular Diseases Mortality Within All Participants and Subgroups of Moderators The conversion from Japanese yen to US dollars based on the exchange rate as of May 1, 2024, was $1 = ¥157.36.

Figure 2 shows the associations between social isolation and 6-year CVD mortality stratified by moderators. Overall, the 95% CIs of ORs for CVD mortality in each moderator stratum were wide, and the evidence was inconclusive. A notable association was found among people in the second income tertiles of households (OR, 1.61 [95% CI, 1.15-2.25]) and those in the highest population density tertile (OR, 1.50 [95% CI, 1.04-2.17]). However, there was no evidence of effect modification based on estimates of effect modification (eTable 4 in Supplement 1).

**Figure 3.**
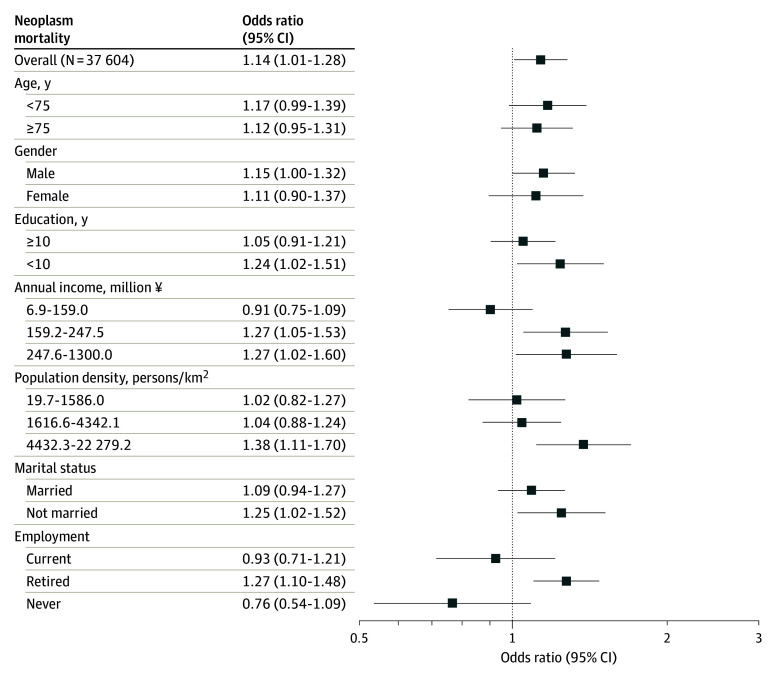
Odds Ratios of Social Isolation for Malignant Neoplasms Mortality Within All Participants and Subgroups of Moderators The conversion from Japanese yen to US dollars based on the exchange rate as of May 1, 2024, was $1 = ¥157.36.

Figure 3 shows the associations between social isolation and 6-year neoplasm mortality, stratified by moderators. Overall trends were similar to those observed in all-cause mortality. Associations between social isolation and malignant neoplasm mortality were consistent across age groups, in men and women, and across low and high-education groups. Notable associations with malignant neoplasm mortality were found in some subgroups: the second and third household income tertiles (second tertile: OR, 1.27 [95% CI, 1.05-1.53]; third tertile: OR, 1.27 [95% CI, 1.02-1.60]); the highest population density tertile (OR, 1.38 [95% CI, 1.11-1.70]); unmarried individuals (OR, 1.25 [95% CI, 1.02-1.52]); and retirees (OR, 1.27 [95% CI, 1.10-1.48]). eTable 5 in Supplement 1 details effect modification measures (RERIs and RORs), with evidence of modification by household income and employment, supporting the above-mentioned associations of subgroups.

We conducted 5 sensitivity analyses. Overall, trends aligned with the main analysis (eTables 4-9 and eFigures 2-19 in Supplement 1).

## Discussion

In our study of older Japanese adults, we found varying associations of social isolation with mortality based on income, population density, and marital and employment status. We observed effect modification by population density and employment for all-cause mortality and by income and employment for malignant neoplasm mortality. The trends on additive and multiplicative scales were similar. Despite no clear heterogeneity in CVD mortality, caution is needed due to the wide confidence interval from fewer CVD mortality cases.

Individuals with middle and high incomes may be more susceptible to mortality from all causes and malignant neoplasms due to social isolation. This contrasts with the lower-income group, often seen as socially vulnerable, who might be less affected by the negative impacts of social isolation. Research in this domain remains sparse.^1^ A potential explanation for this is the concept of relative deprivation, where individuals evaluate their well-being in comparison with their reference group.^22,23^ Our study and prior research indicate that higher-income groups have lower social isolation prevalence. In these groups, the experience of social isolation may feel more acute as individuals compare themselves with more socially connected affluent peers. This contrast, stemming from unmet social expectations, may intensify the psychological and health impacts of isolation.

Our analysis indicates that social isolation could more significantly affect all-cause and neoplasm mortality in high population density areas, a finding supported by effect modification tests for all-cause mortality. Research suggests rural residents are less isolated and more family-reliant than urban ones.^12,24^ Abundant family support in rural areas may buffer the impact of social isolation, highlighting the importance of family or alternative support systems in urban areas. However, this is speculative, highlighting the need for future research to explore these mechanisms.

The analysis suggests that nonmarried individuals could be more impacted by social isolation, which may influence mortality. Our previous research indicates that social engagement, such as social interaction and participation, might mitigate this effect, especially regarding depression after bereavement.^25^ This means that the lack of social connection may exacerbate depressive symptoms following widowhood. Therefore, social isolation might pose a greater risk for psychological stress among the nonmarried, particularly those who are widowed. Providing opportunities for social connection after bereavement may be an effective approach to reducing the risk of mortality.

Our analysis indicates that older adults in Japan who maintain social connections postretirement may experience a lower risk of all-cause and malignant neoplasm mortality, potentially due to reduced psychological stress from the retirement transition. Supporting this, a 2021 meta-analysis found a significant correlation between retirement in Eastern developed countries and depressive symptoms.^26^ Additionally, a Japanese study indicated that older adults engaged in recreational social activities experienced fewer depressive symptoms related to retirement.^27^ Therefore, a lack of social connections (ie, social isolation) after retirement might exacerbate health conditions, probably leading to premature mortality. Support for transitioning to a community network from a workplace network after retirement may be effective to prevent people from becoming isolated. However, it is uncertain whether these findings apply to Western countries. Further research is needed to examine regional variations in the impact of social isolation on mortality among retirees.

We found similar heterogeneity trends in both all-cause and malignant neoplasm mortality, possibly because of high prevalence of malignant neoplasm–related deaths influencing overall mortality results. In contrast, social isolation and CVD mortality associations showed different heterogeneity patterns. Considering potential mechanisms such as health behaviors and psychological and physiological factors^6^ and their different impacts on CVD and malignant neoplasms, heterogeneity is expected. However, our study’s limited CVD mortality cases restrict robust evidence of demographic and social factor influences, underlining future research needs.

### Limitations

This study had several limitations. In interpreting our findings, consider these methodological issues: first, our longitudinal data are observational; hence, reverse causation cannot be entirely ruled out. Second, unmeasured confounding is a possibility; E-values were used to assess the minimum strength of such confounding necessary to negate our observed associations. Third, there is no universally accepted standard for measuring social isolation—our scale, based on UK Biobank data studies,^16^ comprises 3 components. However, due to data limitations, it did not capture some dimensions, such as the interaction with relatives or remote communication included in the Berkman–Syme Social Network Index.^28^ Fourth, our study did not examine multidimensional heterogeneity; age, gender, income, and employment may collectively influence the association between social isolation and mortality. Future research, potentially employing machine learning, could explore this complex heterogeneity.^29^ Fifth, we could not determine the temporal sequence of exposure, moderators, and confounding factors due to simultaneous baseline assessments. Adjustments for potential mediators might lead to underestimated associations. Sixth, our model could not consider exposure-mediator confounding. Seventh, the wide 95% CIs for CVD mortality, likely from few cases, necessitate cautious interpretation. Eighth, our analysis did not include loneliness due to data limitations, highlighting potential for future research to unravel its relationship with social isolation. Lastly, generalizability of the findings should be noted. Differences in multiple factors such as social norms, health care systems, and social support mechanisms across cultures may influence the heterogeneity in the association between social isolation and mortality.

## Conclusions

Our study highlights the heterogeneity of the association between social isolation and increased mortality from all causes, CVD, and malignant neoplasms. Income, population density, marital, and employment status were potential moderators. Effect modification testing corroborated effect modification by population density and employment for all-cause mortality and by income and employment for neoplasm mortality. This research fills a gap in understanding the impact of social isolation across different demographic and socioeconomic groups.

## Notes

Supplement 1. **eAppendix 1.** Supplementary Data **eAppendix 2.** R Scripts and STATA Do File **eFigure 1.** Odds Ratios of Social Isolation for All-Cause Mortality, Cardiovascular Diseases Mortality, and Neoplasms Mortality Among Older People in Japan **eFigure 2.** Odds Ratios of Social Isolation for All-Cause Mortality Over Levels of Age at Baseline **eFigure 3.** Odds Ratios of Social Isolation for Cardiovascular Diseases Mortality Over Levels of Age at Baseline **eFigure 4.** Odds Ratios of Social Isolation for Neoplasms Mortality Over Levels of Age at Baseline **eFigure 5.** Odds Ratios of Social Isolation for All-Cause Mortality Within All Participants and Subgroups of Moderators: Using Population Density That Excluded Nondeveloped Areas (eg, Rivers, Lakes, Forests, and Wasteland) as Well as Nonresidential Land (Such as Farms and Industrial Districts) **eFigure 6.** Odds Ratios of Social Isolation for Cardiovascular Diseases Mortality Within All Participants and Subgroups of Moderators: Using Population Density That Excluded Nondeveloped Areas (eg, Rivers, Lakes, Forests, and Wasteland) as Well as Nonresidential Land (Such as Farms and Industrial Districts) **eFigure 7.** Odds Ratios of Social Isolation for Neoplasm Diseases Mortality Within All Participants and Subgroups of Moderators: Using Population Density That Excluded Nondeveloped Areas (eg, Rivers, Lakes, Forests, and Wasteland) as Well as Nonresidential Land (Such as Farms and Industrial Districts) **eFigure 8.** Odds Ratios of Social Isolation for All-Cause Mortality Within All Participants and Subgroups of Moderators: Clustered at Municipal Levels **eFigure 9.** Odds Ratios of Social Isolation for Cardiovascular Disease Mortality Within All Participants and Subgroups of Moderators: Clustered at Municipal Levels **eFigure 10.** Odds Ratios of Social Isolation for Neoplasm Mortality Within All Participants and Subgroups of Moderators: Clustered at Municipal Levels **eFigure 11.** Hazard Ratios of Social Isolation for All-Cause Mortality, Cardiovascular Diseases Mortality, and Neoplasms Mortality With 6-Year Censoring **eFigure 12.** Hazard Ratios of Social Isolation for All-Cause Mortality Within All Participants and Subgroups of Moderators With 6-Year Censoring **eFigure 13.** Hazard Ratios of Social Isolation for Cardiovascular Diseases Mortality Within All Participants and Subgroups of Moderators With 6-Year Censoring **eFigure 14.** Hazard Ratios of Social Isolation for Neoplasms Mortality Within All Participants and Subgroups of Moderators With 6-Year Censoring **eFigure 15.** Hazard Ratios of Social Isolation for All-Cause Mortality Within All Participants and Subgroups of Moderators Without 6-Year Censoring **eFigure 16.** Hazard Ratios of Social Isolation for Cardiovascular Disease Mortality Within All Participants and Subgroups of Moderators Without 6-Year Censoring **eFigure 17.** Hazard Ratios of Social Isolation for Neoplasm Mortality Within All Participants and Subgroups of Moderators Without 6-Year Censoring **eFigure 18.** Kaplan-Meier Curves for All-Cause Mortality, Cardiovascular Disease Mortality, and Neoplasm Mortality by Social Isolation **eFigure 19.** Cumulative Incidence Functions for Cardiovascular Disease Mortality and Neoplasm Mortality by Social Isolation **eTable 1.** Number (Percentage) of Missing Values **eTable 2.** Baseline Characteristics (Obtained From the 2010 Survey) of Unimputed Data (N = 36 589) **eTable 3.** Additive and Multiplicative Estimates of Effect Modification for the Associations Between Social Isolation and All-Cause Mortality Based on Logistic Regression **eTable 4.** Additive and Multiplicative Estimates of Effect Modification for the Associations Between Social Isolation and Cardiovascular Diseases Mortality Based on Logistic Regression **eTable 5.** Additive and Multiplicative Estimates of Effect Modification for the Associations Between Social Isolation and Neoplasms Mortality Based on Logistic Regression **eTable 6.** Robustness to Unmeasured Confounding (E-Values) of Associations Between Social Isolation and All-Cause Mortality, Cardiovascular Diseases Mortality, and Neoplasms Mortality **eTable 7.** Additive and Multiplicative Estimates of Effect Modification for the Associations Between Social Isolation and All-Cause Mortality Based on Survival Regression **eTable 8.** Additive and Multiplicative Estimates of Effect Modification for the Associations Between Social Isolation and Cardiovascular Diseases Mortality Based on Survival Regression **eTable 9.** Additive and Multiplicative Estimates of Effect Modification for the Associations Between Social Isolation and Neoplasms Mortality Based on Survival Regression

Supplement 2.
Data Sharing Statement
