# Supplementary material for: Sociodemographic Heterogeneity in the Associations of Social Isolation With Mortality
Source: JAMA Netw Open. 2024 May 24;7(5):e2413132. doi: 10.1001/jamanetworkopen.2024.13132 (PMC11127126; doi:10.1001/jamanetworkopen.2024.13132)
Supplement: Supplement 1. — eAppendix 1. Supplementary Data eAppendix 2. R Scripts and STATA Do File eFigure 1. Odds Ratios of Social Isolation for All-Cause Mortality, Cardiovascular Diseases Mortality, and Neoplasms Mortality Among Older People in Japan eFigure 2. Odds Ratios of Social Isolation for All-Cause Mortality Over Levels of Age at Baseline eFigure 3. Odds Ratios of Social Isolation for Cardiovascular Diseases Mortality Over Levels of Age at Baseline eFigure 4. Odds Ratios of Social Isolation for Neoplasms Mortality Over Levels of Age at Baseline eFigure 5. Odds Ratios of Social Isolation for All-Cause Mortality Within All Participants and Subgroups of Moderators: Using Population Density That Excluded Nondeveloped Areas (eg, Rivers, Lakes, Forests, and Wasteland) as Well as Nonresidential Land (Such as Farms and Industrial Districts) eFigure 6. Odds Ratios of Social Isolation for Cardiovascular Diseases Mortality Within All Participants and Subgroups of Moderators: Using Population Density That Excluded Nondeveloped Areas (eg, Rivers, Lakes, Forests, and Wasteland) as Well as Nonresidential Land (Such as Farms and Industrial Districts) eFigure 7. Odds Ratios of Social Isolation for Neoplasm Diseases Mortality Within All Participants and Subgroups of Moderators: Using Population Density That Excluded Nondeveloped Areas (eg, Rivers, Lakes, Forests, and Wasteland) as Well as Nonresidential Land (Such as Farms and Industrial Districts) eFigure 8. Odds Ratios of Social Isolation for All-Cause Mortality Within All Participants and Subgroups of Moderators: Clustered at Municipal Levels eFigure 9. Odds Ratios of Social Isolation for Cardiovascular Disease Mortality Within All Participants and Subgroups of Moderators: Clustered at Municipal Levels eFigure 10. Odds Ratios of Social Isolation for Neoplasm Mortality Within All Participants and Subgroups of Moderators: Clustered at Municipal Levels eFigure 11. Hazard Ratios of Social Isolation for All-Cause Mortality, Cardiovascular Diseases Mo [file jamanetwopen-e2413132-s001.pdf]

# Supplemental Online Content

Nakagomi A, Saito M, Ojima T, Ueno T, Hanazato M, Kondo K. Sociodemographic heterogeneity in the associations of social isolation with mortality. *JAMA Netw Open.* 2024;7(5):e2413132. doi:10.1001/jamanetworkopen.2024.13132

**eAppendix 1.** Supplementary Data

**eAppendix 2.** R Scripts and STATA Do File

**eFigure 1.** Odds Ratios of Social Isolation for All-Cause Mortality, Cardiovascular Diseases Mortality, and Neoplasms Mortality Among Older People in Japan

**eFigure 2.** Odds Ratios of Social Isolation for All-Cause Mortality Over Levels of Age at Baseline

**eFigure 3.** Odds Ratios of Social Isolation for Cardiovascular Diseases Mortality Over Levels of Age at Baseline

**eFigure 4.** Odds Ratios of Social Isolation for Neoplasms Mortality Over Levels of Age at Baseline

**eFigure 5.** Odds Ratios of Social Isolation for All-Cause Mortality Within All Participants and Subgroups of Moderators: Using Population Density That Excluded Nondeveloped Areas (eg, Rivers, Lakes, Forests, and Wasteland) as Well as Nonresidential Land (Such as Farms and Industrial Districts)

**eFigure 6.** Odds Ratios of Social Isolation for Cardiovascular Diseases Mortality Within All Participants and Subgroups of Moderators: Using Population Density That Excluded Nondeveloped Areas (eg, Rivers, Lakes, Forests, and Wasteland) as Well as Nonresidential Land (Such as Farms and Industrial Districts)

**eFigure 7.** Odds Ratios of Social Isolation for Neoplasm Diseases Mortality Within All Participants and Subgroups of Moderators: Using Population Density

© 2024 Nakagomi A et al. *JAMA Network Open.*

That Excluded Nondeveloped Areas (eg, Rivers, Lakes, Forests, and Wasteland) as Well as Nonresidential Land (Such as Farms and Industrial Districts)

**eFigure 8.** Odds Ratios of Social Isolation for All-Cause Mortality Within All Participants and Subgroups of Moderators: Clustered at Municipal Levels

**eFigure 9.** Odds Ratios of Social Isolation for Cardiovascular Disease Mortality Within All Participants and Subgroups of Moderators: Clustered at Municipal Levels

**eFigure 10.** Odds Ratios of Social Isolation for Neoplasm Mortality Within All Participants and Subgroups of Moderators: Clustered at Municipal Levels

**eFigure 11.** Hazard Ratios of Social Isolation for All-Cause Mortality, Cardiovascular Diseases Mortality, and Neoplasms Mortality With 6-Year Censoring

**eFigure 12.** Hazard Ratios of Social Isolation for All-Cause Mortality Within All Participants and Subgroups of Moderators With 6-Year Censoring

**eFigure 13.** Hazard Ratios of Social Isolation for Cardiovascular Diseases Mortality Within All Participants and Subgroups of Moderators With 6-Year Censoring

**eFigure 14.** Hazard Ratios of Social Isolation for Neoplasms Mortality Within All Participants and Subgroups of Moderators With 6-Year Censoring

**eFigure 15.** Hazard Ratios of Social Isolation for All-Cause Mortality Within All Participants and Subgroups of Moderators Without 6-Year Censoring

**eFigure 16.** Hazard Ratios of Social Isolation for Cardiovascular Disease Mortality Within All Participants and Subgroups of Moderators Without 6-Year Censoring

**eFigure 17.** Hazard Ratios of Social Isolation for Neoplasm Mortality Within All Participants and Subgroups of Moderators Without 6-Year Censoring

**eFigure 18.** Kaplan-Meier Curves for All-Cause Mortality, Cardiovascular Disease Mortality, and Neoplasm Mortality by Social Isolation

**eFigure 19.** Cumulative Incidence Functions for Cardiovascular Disease Mortality and Neoplasm Mortality by Social Isolation

**eTable 1.** Number (Percentage) of Missing Values

**eTable 2.** Baseline Characteristics (Obtained From the 2010 Survey) of Unimputed Data (N = 36 589)

**eTable 3.** Additive and Multiplicative Estimates of Effect Modification for the Associations Between Social Isolation and All-Cause Mortality Based on Logistic Regression

© 2024 Nakagomi A et al. *JAMA Network Open.*

**eTable 4.** Additive and Multiplicative Estimates of Effect Modification for the Associations Between Social Isolation and Cardiovascular Diseases Mortality Based on Logistic Regression

**eTable 5.** Additive and Multiplicative Estimates of Effect Modification for the Associations Between Social Isolation and Neoplasms Mortality Based on Logistic Regression

**eTable 6.** Robustness to Unmeasured Confounding (E-Values) of Associations Between Social Isolation and All-Cause Mortality, Cardiovascular Diseases Mortality, and Neoplasms Mortality

**eTable 7.** Additive and Multiplicative Estimates of Effect Modification for the Associations Between Social Isolation and All-Cause Mortality Based on Survival Regression

**eTable 8.** Additive and Multiplicative Estimates of Effect Modification for the Associations Between Social Isolation and Cardiovascular Diseases Mortality Based on Survival Regression

**eTable 9.** Additive and Multiplicative Estimates of Effect Modification for the Associations Between Social Isolation and Neoplasms Mortality Based on Survival Regression

This supplemental material has been provided by the authors to give readers additional information about their work.

© 2024 Nakagomi A et al. *JAMA Network Open.*

**eAppendix 1.** Supplementary Data

**Definitions of covariates**

Socioeconomic status included years of education (9 years or less, 10 years or more), household equivalized income (in JPY), and population density (per $km^2$) based on Japanese school districts. Household equivalized income was calculated by dividing the annual income by the square root of the number of household members. We defined population density based on Japanese school districts using the 2010 census and Land Utilization Tertiary Mesh Data from the Ministry of Land, Infrastructure, Transport, and Tourism in Japan. These calculations excluded non-developed areas (e.g., rivers, lakes, forests, and wasteland).[1] All spatial calculations were performed using ArcGIS 10.3 software.

Marital status was classified into two categories: married or not married (including single, widowed, divorced, and other). Employment status was categorized as currently working, retired, or never worked. As confounders and potential mediators, we included health behaviors psychological and physiological factors.[2]

Behavioral factors included smoking (never, quit, current), drinking alcohol (never, quit, current), walking (<30min., 30-59, 60-89, ≥90min.), eating meat and fish (every day or not), eating fruit and vegetables (every day or not), and taking health checks (never, more than 4 years ago, 2-3 years, within 1 year).

Psychological factors included depressive symptoms assessed with the short version of the Geriatric Depression Scale (GDS-15), defined as scores of ≥ 5 on the GDS-15 indicating the presence of depressive symptoms (from mild to severe depression).[3]

© 2024 Nakagomi A et al. *JAMA Network Open.*

Physiological factors included body mass index (<18·5, 18·5-25, 25-30, ≥30kg/m2), self-rated health (1=poor; 4=excellent), self-reported hypertension, self-reported diabetes, and self-reported dyslipidemia.

**Determining adjusting variables**

We built three models. Model 1 included age and gender. In model 2, we added socioeconomic status (education, equivalized income, population density), marital status, and employment status as confounders and potential moderators. In model 3, we included health behaviors psychological and physiological factors as confounders and potential mediators. We were unable to identify the temporal order of the exposure and potential mediators and unable to acquire their values post-baseline assessment. Thus, the variables added to Model 3 may function as both confounders and mediators.[2] Consequently, the fully adjusted model was employed in subsequent analyses.

**Sensitivy analysis**

We conducted five sensitivity analyses. First, we calculated the E-values for exposure-outcome associations in each moderator stratum (Supplementary Table 6). E-values were used to quantify the minimum strength of association regarding the odds ratio scale that an unmeasured confounder must have with both the exposure and outcome, above and beyond the adjusted covariates, to explain away the observed associations.[4] For example, in the association between social isolation and all-cause mortality among retirees, an unmeasured confounder would need to be associated with both the exposure and outcome by an OR of 1.78-fold each, conditional on the measured covariates, to

© 2024 Nakagomi A et al. *JAMA Network Open.*

fully explain the observed association. An odds ratio of 1.48-fold would be necessary to shift the CI, allowing for the inclusion of the null value. Second, we graphically represented the ORs associated with changes in social isolation across levels of continuous variables (age, household equivalized income, and population density) using restricted cubic splines with three knots (Supplementary Figure 2, 3, 4). Interaction graphs were constructed by using the interactionRCS package of R. However, the CIs of ORs at higher levels of age, household income, and population density were substantially wide. Third, we incorporated a measure of population density that excluded non-developed areas (e.g., rivers, lakes, forests, and wasteland) as well as non-residential land (such as farms and industrial districts) in the logistic model (Supplementary Figure 5, 6, 7). Fourth, we presented the findings with standard errors clustered at the municipality level, reflecting the level at which sampling was conducted and municipal policies were implemented (Supplementary Figure 8, 9, 10). Fifth, we conducted the same analysis applying Cox proportional hazard models for all-cause mortality and competing-risks survival regression based on Fine and Gray's proportional sub-hazards model for CVD mortality and neoplasm mortality with (Supplementary Figure 12, 13, 14) and without (Supplementary Figure 15, 16, 17) 6 year censoring. We also show the Kaplan-Meier Curves and cumulative incidence functions (Supplementary Figure 18, 19).

1. Fujiwara T, Takamoto I, Amemiya A, et al. Is a hilly neighborhood environment associated with diabetes mellitus among older people? Results from the JAGES 2010 study. *Social Science & Medicine*. 2017;182:45-51.
2. Cené CW, Beckie TM, Sims M, et al. Effects of objective and perceived social isolation on cardiovascular and brain health: a scientific statement from the American Heart Association. *Journal of the American Heart Association*. 2022;11(16):e026493.

© 2024 Nakagomi A et al. *JAMA Network Open.*

3. Sheikh JI, Yesavage JA. Geriatric Depression Scale (GDS): recent evidence and development of a shorter version. *Clinical gerontology*. Routledge; 2014:165-173.
4. VanderWeele TJ. Outcome-wide epidemiology. *Epidemiology (Cambridge, Mass)*. 2017;28(3):399.

© 2024 Nakagomi A et al. *JAMA Network Open.*

**eAppendix 2.** R Scripts and STATA Do File

```
# Load necessary libraries
library(missForest)
library(doParallel)
library(foreach)
library(dplyr)

#read csv file
data <- read.csv("mydata.csv", header = TRUE, stringsAsFactors = FALSE)

# Perform the imputation
imputed <- missForest(data, verbose = TRUE)
# Extract the OOB error
oob_error <- imputed$OOBerror
print(oob_error)
# Extract the imputed dataset
imputed_data <- imputed$ximp

# Write the imputed dataset back to a new CSV file
write.csv(imputed_data, "imputed_data.csv", row.names = FALSE)
```

© 2024 Nakagomi A et al. *JAMA Network Open.*

STATA do file

```
import delimited "imputed_data.csv", clear
*exclude participants without scode (79 observations deleted, n = 46,065)
drop if scode==.

*exclude people with diseases and impaired ADL
drop if dgns2st10==1
drop if dgns2hd10==1
drop if dgns2ca10==1
drop if adl_3_10!=1

***********Logistic regression analysis*****************
*All-cause mortality
*model 1
logistic eve_shibou17_6year i.SI_bi age_ysl10 sex_2_10, cluster(scode)
*model 2
logistic eve_shibou17_6year i.SI_bi age_ysl10 sex_2_10 i.edu2gp c.eqincome10
c.popdens i.marital_bi ib2.empl3pt10, cluster(scode)
*model 3
logistic eve_shibou17_6year i.SI_bi age_ysl10 sex_2_10 i.edu2gp c.eqincome10
c.popdens i.marital_bi ib2.empl3pt10 i.smok3_10 i.alcl3_10 i.walk4tm10
i.fq7prt10re_bi i.fq7veg10re_bi ib4.exam4_10re  i.gds5 ib1.bmi4_10 c.srh_4_10re
i.dgns2ht10 i.dgns2dm10 i.dgns2hl10, cluster(scode)



*CVD mortality
*model 1
logistic eve_sibou_IHD_stroke_6year i.SI_bi age_ysl10 sex_2_10, cluster(scode)
*model 2
```

© 2024 Nakagomi A et al. *JAMA Network Open.*

logistic eve_sibou_IHD_stroke_6year i.SI_bi age_ysl10 sex_2_10 i.edu2gp c.eqincome10 c.popdens i.marital_bi ib2.empl3pt10, cluster(scode)

*model 3

logistic eve_sibou_IHD_stroke_6year i.SI_bi age_ysl10 sex_2_10 i.edu2gp c.eqincome10 c.popdens i.marital_bi ib2.empl3pt10 i.smok3_10 i.alcl3_10 i.walk4tm10 i.fq7prt10re_bi i.fq7veg10re_bi ib4.exam4_10re i.gds5 ib1.bmi4_10 c.srh_4_10re i.dgns2ht10 i.dgns2dm10 i.dgns2hl10, cluster(scode)

*Neoplasms

*model 1

logistic eve_sibou_cancer_6year i.SI_bi age_ysl10 sex_2_10, cluster(scode)

*model 2

logistic eve_sibou_cancer_6year i.SI_bi age_ysl10 sex_2_10 i.edu2gp c.eqincome10 c.popdens i.marital_bi ib2.empl3pt10, cluster(scode)

*model 3

logistic eve_sibou_cancer_6year i.SI_bi age_ysl10 sex_2_10 i.edu2gp c.eqincome10 c.popdens i.marital_bi ib2.empl3pt10 i.smok3_10 i.alcl3_10 i.walk4tm10 i.fq7prt10re_bi i.fq7veg10re_bi ib4.exam4_10re i.gds5 ib1.bmi4_10 c.srh_4_10re i.dgns2ht10 i.dgns2dm10 i.dgns2hl10, cluster(scode)

**Modification_logistic

*All-cause mortality

*effect modification

logistic eve_shibou17_6year i.SI_bi##i.age75 age_ysl10 sex_2_10 i.edu2gp c.eqincome10 c.popdens i.marital_bi ib2.empl3pt10 i.smok3_10 i.alcl3_10 i.walk4tm10 i.fq7prt10re_bi i.fq7veg10re_bi ib4.exam4_10re i.gds5 ib1.bmi4_10 c.srh_4_10re i.dgns2ht10 i.dgns2dm10 i.dgns2hl10, cluster(scode)

logistic eve_shibou17_6year i.SI_bi##i.sex_2_10 age_ysl10 sex_2_10 i.edu2gp c.eqincome10 c.popdens i.marital_bi ib2.empl3pt10 i.smok3_10 i.alcl3_10 i.walk4tm10 i.fq7prt10re_bi i.fq7veg10re_bi ib4.exam4_10re i.gds5 ib1.bmi4_10 c.srh_4_10re i.dgns2ht10 i.dgns2dm10 i.dgns2hl10, cluster(scode)

© 2024 Nakagomi A et al. *JAMA Network Open.*

```
logistic eve_shibou17_6year i.SI_bi##i.edu2gp age_ysl10 sex_2_10 i.edu2gp c.eqincome10 c.popdens i.marital_bi ib2.empl3pt10 i.smok3_10 i.alcl3_10 i.walk4tm10 i.fq7prt10re_bi i.fq7veg10re_bi ib4.exam4_10re  i.gds5 ib1.bmi4_10 c.srh_4_10re i.dgns2ht10 i.dgns2dm10 i.dgns2hl10, cluster(scode)
```

```
logistic eve_shibou17_6year i.SI_bi##i.income_tertile age_ysl10 sex_2_10 i.edu2gp c.eqincome10 c.popdens i.marital_bi ib2.empl3pt10 i.smok3_10 i.alcl3_10 i.walk4tm10 i.fq7prt10re_bi i.fq7veg10re_bi ib4.exam4_10re  i.gds5 ib1.bmi4_10 c.srh_4_10re i.dgns2ht10 i.dgns2dm10 i.dgns2hl10, cluster(scode)
```

```
logistic eve_shibou17_6year i.SI_bi##i.popdens_tertile age_ysl10 sex_2_10 i.edu2gp c.eqincome10 c.popdens i.marital_bi ib2.empl3pt10 i.smok3_10 i.alcl3_10 i.walk4tm10 i.fq7prt10re_bi i.fq7veg10re_bi ib4.exam4_10re  i.gds5 ib1.bmi4_10 c.srh_4_10re i.dgns2ht10 i.dgns2dm10 i.dgns2hl10, cluster(scode)
```

```
logistic eve_shibou17_6year i.SI_bi##i.marital_bi age_ysl10 sex_2_10 i.edu2gp c.eqincome10 c.popdens i.marital_bi ib2.empl3pt10 i.smok3_10 i.alcl3_10 i.walk4tm10 i.fq7prt10re_bi i.fq7veg10re_bi ib4.exam4_10re  i.gds5 ib1.bmi4_10 c.srh_4_10re i.dgns2ht10 i.dgns2dm10 i.dgns2hl10, cluster(scode)
```

```
logistic eve_shibou17_6year i.SI_bi##ib2.empl3pt10 age_ysl10 sex_2_10 i.edu2gp c.eqincome10 c.popdens i.marital_bi ib2.empl3pt10 i.smok3_10 i.alcl3_10 i.walk4tm10 i.fq7prt10re_bi i.fq7veg10re_bi ib4.exam4_10re  i.gds5 ib1.bmi4_10 c.srh_4_10re i.dgns2ht10 i.dgns2dm10 i.dgns2hl10, cluster(scode)
```

```
reri logistic eve_shibou17_6year SI_bi age75 age_ysl10 sex_2_10 i.edu2gp c.eqincome10 c.popdens i.marital_bi ib2.empl3pt10 i.smok3_10 i.alcl3_10 i.walk4tm10 i.fq7prt10re_bi i.fq7veg10re_bi ib4.exam4_10re  i.gds5 ib1.bmi4_10 c.srh_4_10re i.dgns2ht10 i.dgns2dm10 i.dgns2hl10, cluster(scode)
```

```
reri logistic eve_shibou17_6year SI_bi sex_2_10 age_ysl10 i.edu2gp c.eqincome10 c.popdens i.marital_bi ib2.empl3pt10 i.smok3_10 i.alcl3_10 i.walk4tm10 i.fq7prt10re_bi i.fq7veg10re_bi ib4.exam4_10re  i.gds5 ib1.bmi4_10 c.srh_4_10re i.dgns2ht10 i.dgns2dm10 i.dgns2hl10, cluster(scode)
```

```
reri logistic eve_shibou17_6year SI_bi edu2gp age_ysl10 sex_2_10  c.eqincome10 c.popdens i.marital_bi ib2.empl3pt10 i.smok3_10 i.alcl3_10 i.walk4tm10 i.fq7prt10re_bi i.fq7veg10re_bi ib4.exam4_10re  i.gds5 ib1.bmi4_10 c.srh_4_10re i.dgns2ht10 i.dgns2dm10 i.dgns2hl10, cluster(scode)
```

© 2024 Nakagomi A et al. *JAMA Network Open.*

reri logistic eve_shibou17_6year SI_bi income_tertile_d2 age_ysl10 sex_2_10 i.edu2gp c.eqincome10 c.popdens i.marital_bi ib2.empl3pt10 i.smok3_10 i.alcl3_10 i.walk4tm10 i.fq7prt10re_bi i.fq7veg10re_bi ib4.exam4_10re i.gds5 ib1.bmi4_10 c.srh_4_10re i.dgns2ht10 i.dgns2dm10 i.dgns2hl10 if income_tertile_d3==0, cluster(scode)

reri logistic eve_shibou17_6year SI_bi income_tertile_d3 age_ysl10 sex_2_10 i.edu2gp c.eqincome10 c.popdens i.marital_bi ib2.empl3pt10 i.smok3_10 i.alcl3_10 i.walk4tm10 i.fq7prt10re_bi i.fq7veg10re_bi ib4.exam4_10re i.gds5 ib1.bmi4_10 c.srh_4_10re i.dgns2ht10 i.dgns2dm10 i.dgns2hl10 if income_tertile_d2==0, cluster(scode)

reri logistic eve_shibou17_6year SI_bi popdens_tertile_d2 age_ysl10 sex_2_10 i.edu2gp c.eqincome10 c.popdens i.marital_bi ib2.empl3pt10 i.smok3_10 i.alcl3_10 i.walk4tm10 i.fq7prt10re_bi i.fq7veg10re_bi ib4.exam4_10re i.gds5 ib1.bmi4_10 c.srh_4_10re i.dgns2ht10 i.dgns2dm10 i.dgns2hl10 if popdens_tertile_d3==0, cluster(scode)

reri logistic eve_shibou17_6year SI_bi popdens_tertile_d3 age_ysl10 sex_2_10 i.edu2gp c.eqincome10 c.popdens i.marital_bi ib2.empl3pt10 i.smok3_10 i.alcl3_10 i.walk4tm10 i.fq7prt10re_bi i.fq7veg10re_bi ib4.exam4_10re i.gds5 ib1.bmi4_10 c.srh_4_10re i.dgns2ht10 i.dgns2dm10 i.dgns2hl10 if popdens_tertile_d2==0, cluster(scode)

reri logistic eve_shibou17_6year SI_bi marital_bi age_ysl10 sex_2_10 i.edu2gp c.eqincome10 c.popdens ib2.empl3pt10 i.smok3_10 i.alcl3_10 i.walk4tm10 i.fq7prt10re_bi i.fq7veg10re_bi ib4.exam4_10re i.gds5 ib1.bmi4_10 c.srh_4_10re i.dgns2ht10 i.dgns2dm10 i.dgns2hl10, cluster(scode)

reri logistic eve_shibou17_6year SI_bi empl3pt10_d1 age_ysl10 sex_2_10 i.edu2gp c.eqincome10 c.popdens i.marital_bi i.smok3_10 i.alcl3_10 i.walk4tm10 i.fq7prt10re_bi i.fq7veg10re_bi ib4.exam4_10re i.gds5 ib1.bmi4_10 c.srh_4_10re i.dgns2ht10 i.dgns2dm10 i.dgns2hl10 if empl3pt10_d3==0, cluster(scode)

reri logistic eve_shibou17_6year SI_bi empl3pt10_d3 age_ysl10 sex_2_10 i.edu2gp c.eqincome10 c.popdens i.marital_bi i.smok3_10 i.alcl3_10 i.walk4tm10 i.fq7prt10re_bi i.fq7veg10re_bi ib4.exam4_10re i.gds5 ib1.bmi4_10 c.srh_4_10re i.dgns2ht10 i.dgns2dm10 i.dgns2hl10 if empl3pt10_d1==0, cluster(scode)

*subgroup

bysort age75: logistic eve_shibou17_6year i.SI_bi age_ysl10 sex_2_10 i.edu2gp c.eqincome10 c.popdens i.marital_bi ib2.empl3pt10 i.smok3_10 i.alcl3_10 i.walk4tm10 i.fq7prt10re_bi i.fq7veg10re_bi ib4.exam4_10re i.gds5 ib1.bmi4_10 c.srh_4_10re i.dgns2ht10 i.dgns2dm10 i.dgns2hl10, cluster(scode)

© 2024 Nakagomi A et al. *JAMA Network Open.*

bysort sex_2_10: logistic eve_shibou17_6year i.SI_bi age_ysl10 sex_2_10 i.edu2gp c.eqincome10 c.popdens i.marital_bi ib2.empl3pt10 i.smok3_10 i.alcl3_10 i.walk4tm10 i.fq7prt10re_bi i.fq7veg10re_bi ib4.exam4_10re i.gds5 ib1.bmi4_10 c.srh_4_10re i.dgns2ht10 i.dgns2dm10 i.dgns2hl10, cluster(scode)

bysort edu2gp: logistic eve_shibou17_6year i.SI_bi age_ysl10 sex_2_10 i.edu2gp c.eqincome10 c.popdens i.marital_bi ib2.empl3pt10 i.smok3_10 i.alcl3_10 i.walk4tm10 i.fq7prt10re_bi i.fq7veg10re_bi ib4.exam4_10re i.gds5 ib1.bmi4_10 c.srh_4_10re i.dgns2ht10 i.dgns2dm10 i.dgns2hl10, cluster(scode)

bysort income_tertile: logistic eve_shibou17_6year i.SI_bi age_ysl10 sex_2_10 i.edu2gp c.eqincome10 c.popdens i.marital_bi ib2.empl3pt10 i.smok3_10 i.alcl3_10 i.walk4tm10 i.fq7prt10re_bi i.fq7veg10re_bi ib4.exam4_10re i.gds5 ib1.bmi4_10 c.srh_4_10re i.dgns2ht10 i.dgns2dm10 i.dgns2hl10, cluster(scode)

bysort popdens_tertile: logistic eve_shibou17_6year i.SI_bi age_ysl10 sex_2_10 i.edu2gp c.eqincome10 c.popdens i.marital_bi ib2.empl3pt10 i.smok3_10 i.alcl3_10 i.walk4tm10 i.fq7prt10re_bi i.fq7veg10re_bi ib4.exam4_10re i.gds5 ib1.bmi4_10 c.srh_4_10re i.dgns2ht10 i.dgns2dm10 i.dgns2hl10, cluster(scode)

bysort marital_bi: logistic eve_shibou17_6year i.SI_bi age_ysl10 sex_2_10 i.edu2gp c.eqincome10 c.popdens i.marital_bi ib2.empl3pt10 i.smok3_10 i.alcl3_10 i.walk4tm10 i.fq7prt10re_bi i.fq7veg10re_bi ib4.exam4_10re i.gds5 ib1.bmi4_10 c.srh_4_10re i.dgns2ht10 i.dgns2dm10 i.dgns2hl10, cluster(scode)

bysort empl3pt10: logistic eve_shibou17_6year i.SI_bi age_ysl10 sex_2_10 i.edu2gp c.eqincome10 c.popdens i.marital_bi ib2.empl3pt10 i.smok3_10 i.alcl3_10 i.walk4tm10 i.fq7prt10re_bi i.fq7veg10re_bi ib4.exam4_10re i.gds5 ib1.bmi4_10 c.srh_4_10re i.dgns2ht10 i.dgns2dm10 i.dgns2hl10, cluster(scode)

*CVD mortality

*effect modification

logistic eve_sibou_IHD_stroke_6year i.SI_bi##i.age75 age_ysl10 sex_2_10 i.edu2gp c.eqincome10 c.popdens i.marital_bi ib2.empl3pt10 i.smok3_10 i.alcl3_10 i.walk4tm10 i.fq7prt10re_bi i.fq7veg10re_bi ib4.exam4_10re i.gds5 ib1.bmi4_10 c.srh_4_10re i.dgns2ht10 i.dgns2dm10 i.dgns2hl10, cluster(scode)

© 2024 Nakagomi A et al. *JAMA Network Open.*

logistic eve_sibou_IHD_stroke_6year i.SI_bi##i.sex_2_10 age_ysl10 sex_2_10 i.edu2gp c.eqincome10 c.popdens i.marital_bi ib2.empl3pt10 i.smok3_10 i.alcl3_10 i.walk4tm10 i.fq7prt10re_bi i.fq7veg10re_bi ib4.exam4_10re i.gds5 ib1.bmi4_10 c.srh_4_10re i.dgns2ht10 i.dgns2dm10 i.dgns2hl10, cluster(scode)

logistic eve_sibou_IHD_stroke_6year i.SI_bi##i.edu2gp age_ysl10 sex_2_10 i.edu2gp c.eqincome10 c.popdens i.marital_bi ib2.empl3pt10 i.smok3_10 i.alcl3_10 i.walk4tm10 i.fq7prt10re_bi i.fq7veg10re_bi ib4.exam4_10re i.gds5 ib1.bmi4_10 c.srh_4_10re i.dgns2ht10 i.dgns2dm10 i.dgns2hl10, cluster(scode)

logistic eve_sibou_IHD_stroke_6year i.SI_bi##i.income_tertile age_ysl10 sex_2_10 i.edu2gp c.eqincome10 c.popdens i.marital_bi ib2.empl3pt10 i.smok3_10 i.alcl3_10 i.walk4tm10 i.fq7prt10re_bi i.fq7veg10re_bi ib4.exam4_10re i.gds5 ib1.bmi4_10 c.srh_4_10re i.dgns2ht10 i.dgns2dm10 i.dgns2hl10, cluster(scode)

logistic eve_sibou_IHD_stroke_6year i.SI_bi##i.popdens_tertile age_ysl10 sex_2_10 i.edu2gp c.eqincome10 c.popdens i.marital_bi ib2.empl3pt10 i.smok3_10 i.alcl3_10 i.walk4tm10 i.fq7prt10re_bi i.fq7veg10re_bi ib4.exam4_10re i.gds5 ib1.bmi4_10 c.srh_4_10re i.dgns2ht10 i.dgns2dm10 i.dgns2hl10, cluster(scode)

logistic eve_sibou_IHD_stroke_6year i.SI_bi##i.marital_bi age_ysl10 sex_2_10 i.edu2gp c.eqincome10 c.popdens i.marital_bi ib2.empl3pt10 i.smok3_10 i.alcl3_10 i.walk4tm10 i.fq7prt10re_bi i.fq7veg10re_bi ib4.exam4_10re i.gds5 ib1.bmi4_10 c.srh_4_10re i.dgns2ht10 i.dgns2dm10 i.dgns2hl10, cluster(scode)

logistic eve_sibou_IHD_stroke_6year i.SI_bi##ib2.empl3pt10 age_ysl10 sex_2_10 i.edu2gp c.eqincome10 c.popdens i.marital_bi ib2.empl3pt10 i.smok3_10 i.alcl3_10 i.walk4tm10 i.fq7prt10re_bi i.fq7veg10re_bi ib4.exam4_10re i.gds5 ib1.bmi4_10 c.srh_4_10re i.dgns2ht10 i.dgns2dm10 i.dgns2hl10, cluster(scode)

*RERI

reri logistic eve_sibou_IHD_stroke_6year SI_bi age75 age_ysl10 sex_2_10 i.edu2gp c.eqincome10 c.popdens i.marital_bi ib2.empl3pt10 i.smok3_10 i.alcl3_10 i.walk4tm10 i.fq7prt10re_bi i.fq7veg10re_bi ib4.exam4_10re i.gds5 ib1.bmi4_10 c.srh_4_10re i.dgns2ht10 i.dgns2dm10 i.dgns2hl10, cluster(scode)

© 2024 Nakagomi A et al. *JAMA Network Open.*

```
reri logistic eve_sibou_IHD_stroke_6year SI_bi sex_2_10 age_ysl10 i.edu2gp c.eqincome10 c.popdens i.marital_bi ib2.empl3pt10 i.smok3_10 i.alcl3_10 i.walk4tm10 i.fq7prt10re_bi i.fq7veg10re_bi ib4.exam4_10re  i.gds5 ib1.bmi4_10 c.srh_4_10re i.dgns2ht10 i.dgns2dm10 i.dgns2hl10, cluster(scode)

reri logistic eve_sibou_IHD_stroke_6year SI_bi edu2gp age_ysl10 sex_2_10 c.eqincome10 c.popdens i.marital_bi ib2.empl3pt10 i.smok3_10 i.alcl3_10 i.walk4tm10 i.fq7prt10re_bi i.fq7veg10re_bi ib4.exam4_10re  i.gds5 ib1.bmi4_10 c.srh_4_10re i.dgns2ht10 i.dgns2dm10 i.dgns2hl10, cluster(scode)

reri logistic eve_sibou_IHD_stroke_6year SI_bi income_tertile_d2  age_ysl10 sex_2_10 i.edu2gp c.eqincome10 c.popdens i.marital_bi ib2.empl3pt10 i.smok3_10 i.alcl3_10 i.walk4tm10 i.fq7prt10re_bi i.fq7veg10re_bi ib4.exam4_10re  i.gds5 ib1.bmi4_10 c.srh_4_10re i.dgns2ht10 i.dgns2dm10 i.dgns2hl10 if income_tertile_d3==0, cluster(scode)

reri logistic eve_sibou_IHD_stroke_6year SI_bi income_tertile_d3  age_ysl10 sex_2_10 i.edu2gp c.eqincome10 c.popdens i.marital_bi ib2.empl3pt10 i.smok3_10 i.alcl3_10 i.walk4tm10 i.fq7prt10re_bi i.fq7veg10re_bi ib4.exam4_10re  i.gds5 ib1.bmi4_10 c.srh_4_10re i.dgns2ht10 i.dgns2dm10 i.dgns2hl10 if income_tertile_d2==0, cluster(scode)

reri logistic eve_sibou_IHD_stroke_6year SI_bi popdens_tertile_d2  age_ysl10 sex_2_10 i.edu2gp c.eqincome10 c.popdens i.marital_bi ib2.empl3pt10 i.smok3_10 i.alcl3_10 i.walk4tm10 i.fq7prt10re_bi i.fq7veg10re_bi ib4.exam4_10re  i.gds5 ib1.bmi4_10 c.srh_4_10re i.dgns2ht10 i.dgns2dm10 i.dgns2hl10 if popdens_tertile_d3==0, cluster(scode)

reri logistic eve_sibou_IHD_stroke_6year SI_bi popdens_tertile_d3  age_ysl10 sex_2_10 i.edu2gp c.eqincome10 c.popdens i.marital_bi ib2.empl3pt10 i.smok3_10 i.alcl3_10 i.walk4tm10 i.fq7prt10re_bi i.fq7veg10re_bi ib4.exam4_10re  i.gds5 ib1.bmi4_10 c.srh_4_10re i.dgns2ht10 i.dgns2dm10 i.dgns2hl10 if popdens_tertile_d2==0, cluster(scode)

reri logistic eve_sibou_IHD_stroke_6year SI_bi marital_bi age_ysl10 sex_2_10 i.edu2gp c.eqincome10 c.popdens ib2.empl3pt10 i.smok3_10 i.alcl3_10 i.walk4tm10 i.fq7prt10re_bi i.fq7veg10re_bi ib4.exam4_10re  i.gds5 ib1.bmi4_10 c.srh_4_10re i.dgns2ht10 i.dgns2dm10 i.dgns2hl10, cluster(scode)

reri logistic eve_sibou_IHD_stroke_6year SI_bi empl3pt10_d1  age_ysl10 sex_2_10 i.edu2gp c.eqincome10 c.popdens i.marital_bi i.smok3_10 i.alcl3_10 i.walk4tm10 i.fq7prt10re_bi i.fq7veg10re_bi ib4.exam4_10re  i.gds5 ib1.bmi4_10 c.srh_4_10re i.dgns2ht10 i.dgns2dm10 i.dgns2hl10 if empl3pt10_d3==0, cluster(scode)

reri logistic eve_sibou_IHD_stroke_6year SI_bi empl3pt10_d3  age_ysl10 sex_2_10 i.edu2gp c.eqincome10 c.popdens i.marital_bi i.smok3_10 i.alcl3_10 i.walk4tm10
```

© 2024 Nakagomi A et al. *JAMA Network Open.*

i.fq7prt10re_bi i.fq7veg10re_bi ib4.exam4_10re i.gds5 ib1.bmi4_10 c.srh_4_10re i.dgns2ht10 i.dgns2dm10 i.dgns2hl10 if empl3pt10_d1==0, cluster(scode)

*subgroup

bysort age75: logistic eve_sibou_IHD_stroke_6year i.SI_bi age_ysl10 sex_2_10 i.edu2gp c.eqincome10 c.popdens i.marital_bi ib2.empl3pt10 i.smok3_10 i.alcl3_10 i.walk4tm10 i.fq7prt10re_bi i.fq7veg10re_bi ib4.exam4_10re i.gds5 ib1.bmi4_10 c.srh_4_10re i.dgns2ht10 i.dgns2dm10 i.dgns2hl10, cluster(scode)

bysort sex_2_10: logistic eve_sibou_IHD_stroke_6year i.SI_bi age_ysl10 sex_2_10 i.edu2gp c.eqincome10 c.popdens i.marital_bi ib2.empl3pt10 i.smok3_10 i.alcl3_10 i.walk4tm10 i.fq7prt10re_bi i.fq7veg10re_bi ib4.exam4_10re i.gds5 ib1.bmi4_10 c.srh_4_10re i.dgns2ht10 i.dgns2dm10 i.dgns2hl10, cluster(scode)

bysort edu2gp: logistic eve_sibou_IHD_stroke_6year i.SI_bi age_ysl10 sex_2_10 i.edu2gp c.eqincome10 c.popdens i.marital_bi ib2.empl3pt10 i.smok3_10 i.alcl3_10 i.walk4tm10 i.fq7prt10re_bi i.fq7veg10re_bi ib4.exam4_10re i.gds5 ib1.bmi4_10 c.srh_4_10re i.dgns2ht10 i.dgns2dm10 i.dgns2hl10, cluster(scode)

bysort income_tertile: logistic eve_sibou_IHD_stroke_6year i.SI_bi age_ysl10 sex_2_10 i.edu2gp c.eqincome10 c.popdens i.marital_bi ib2.empl3pt10 i.smok3_10 i.alcl3_10 i.walk4tm10 i.fq7prt10re_bi i.fq7veg10re_bi ib4.exam4_10re i.gds5 ib1.bmi4_10 c.srh_4_10re i.dgns2ht10 i.dgns2dm10 i.dgns2hl10, cluster(scode)

bysort popdens_tertile: logistic eve_sibou_IHD_stroke_6year i.SI_bi age_ysl10 sex_2_10 i.edu2gp c.eqincome10 c.popdens i.marital_bi ib2.empl3pt10 i.smok3_10 i.alcl3_10 i.walk4tm10 i.fq7prt10re_bi i.fq7veg10re_bi ib4.exam4_10re i.gds5 ib1.bmi4_10 c.srh_4_10re i.dgns2ht10 i.dgns2dm10 i.dgns2hl10, cluster(scode)

bysort marital_bi: logistic eve_sibou_IHD_stroke_6year i.SI_bi age_ysl10 sex_2_10 i.edu2gp c.eqincome10 c.popdens i.marital_bi ib2.empl3pt10 i.smok3_10 i.alcl3_10 i.walk4tm10 i.fq7prt10re_bi i.fq7veg10re_bi ib4.exam4_10re i.gds5 ib1.bmi4_10 c.srh_4_10re i.dgns2ht10 i.dgns2dm10 i.dgns2hl10, cluster(scode)

bysort empl3pt10: logistic eve_sibou_IHD_stroke_6year i.SI_bi age_ysl10 sex_2_10 i.edu2gp c.eqincome10 c.popdens i.marital_bi ib2.empl3pt10 i.smok3_10 i.alcl3_10

© 2024 Nakagomi A et al. *JAMA Network Open.*

i.walk4tm10 i.fq7prt10re_bi i.fq7veg10re_bi ib4.exam4_10re i.gds5 ib1.bmi4_10 c.srh_4_10re i.dgns2ht10 i.dgns2dm10 i.dgns2hl10, cluster(scode)

*Cancer mortality

*effect modification

logistic eve_sibou_cancer_6year i.SI_bi##i.age75 age_ysl10 sex_2_10 i.edu2gp c.eqincome10 c.popdens i.marital_bi ib2.empl3pt10 i.smok3_10 i.alcl3_10 i.walk4tm10 i.fq7prt10re_bi i.fq7veg10re_bi ib4.exam4_10re i.gds5 ib1.bmi4_10 c.srh_4_10re i.dgns2ht10 i.dgns2dm10 i.dgns2hl10, cluster(scode)

logistic eve_sibou_cancer_6year i.SI_bi##i.sex_2_10 age_ysl10 sex_2_10 i.edu2gp c.eqincome10 c.popdens i.marital_bi ib2.empl3pt10 i.smok3_10 i.alcl3_10 i.walk4tm10 i.fq7prt10re_bi i.fq7veg10re_bi ib4.exam4_10re i.gds5 ib1.bmi4_10 c.srh_4_10re i.dgns2ht10 i.dgns2dm10 i.dgns2hl10, cluster(scode)

logistic eve_sibou_cancer_6year i.SI_bi##i.edu2gp age_ysl10 sex_2_10 i.edu2gp c.eqincome10 c.popdens i.marital_bi ib2.empl3pt10 i.smok3_10 i.alcl3_10 i.walk4tm10 i.fq7prt10re_bi i.fq7veg10re_bi ib4.exam4_10re i.gds5 ib1.bmi4_10 c.srh_4_10re i.dgns2ht10 i.dgns2dm10 i.dgns2hl10, cluster(scode)

logistic eve_sibou_cancer_6year i.SI_bi##i.income_tertile age_ysl10 sex_2_10 i.edu2gp c.eqincome10 c.popdens i.marital_bi ib2.empl3pt10 i.smok3_10 i.alcl3_10 i.walk4tm10 i.fq7prt10re_bi i.fq7veg10re_bi ib4.exam4_10re i.gds5 ib1.bmi4_10 c.srh_4_10re i.dgns2ht10 i.dgns2dm10 i.dgns2hl10, cluster(scode)

logistic eve_sibou_cancer_6year i.SI_bi##i.popdens_tertile age_ysl10 sex_2_10 i.edu2gp c.eqincome10 c.popdens i.marital_bi ib2.empl3pt10 i.smok3_10 i.alcl3_10 i.walk4tm10 i.fq7prt10re_bi i.fq7veg10re_bi ib4.exam4_10re i.gds5 ib1.bmi4_10 c.srh_4_10re i.dgns2ht10 i.dgns2dm10 i.dgns2hl10, cluster(scode)

logistic eve_sibou_cancer_6year i.SI_bi##i.marital_bi age_ysl10 sex_2_10 i.edu2gp c.eqincome10 c.popdens i.marital_bi ib2.empl3pt10 i.smok3_10 i.alcl3_10 i.walk4tm10

© 2024 Nakagomi A et al. *JAMA Network Open.*

i.fq7prt10re_bi i.fq7veg10re_bi ib4.exam4_10re  i.gds5 ib1.bmi4_10 c.srh_4_10re i.dgns2ht10 i.dgns2dm10 i.dgns2hl10, cluster(scode)

logistic eve_sibou_cancer_6year i.SI_bi##ib2.empl3pt10 age_ysl10 sex_2_10 i.edu2gp c.eqincome10 c.popdens i.marital_bi ib2.empl3pt10 i.smok3_10 i.alcl3_10 i.walk4tm10 i.fq7prt10re_bi i.fq7veg10re_bi ib4.exam4_10re  i.gds5 ib1.bmi4_10 c.srh_4_10re i.dgns2ht10 i.dgns2dm10 i.dgns2hl10, cluster(scode)

*RERI

reri logistic eve_sibou_cancer_6year SI_bi age75 age_ysl10 sex_2_10 i.edu2gp c.eqincome10 c.popdens i.marital_bi ib2.empl3pt10 i.smok3_10 i.alcl3_10 i.walk4tm10 i.fq7prt10re_bi i.fq7veg10re_bi ib4.exam4_10re  i.gds5 ib1.bmi4_10 c.srh_4_10re i.dgns2ht10 i.dgns2dm10 i.dgns2hl10, cluster(scode)

reri logistic eve_sibou_cancer_6year SI_bi sex_2_10 age_ysl10 i.edu2gp c.eqincome10 c.popdens i.marital_bi ib2.empl3pt10 i.smok3_10 i.alcl3_10 i.walk4tm10 i.fq7prt10re_bi i.fq7veg10re_bi ib4.exam4_10re  i.gds5 ib1.bmi4_10 c.srh_4_10re i.dgns2ht10 i.dgns2dm10 i.dgns2hl10, cluster(scode)

reri logistic eve_sibou_cancer_6year SI_bi edu2gp age_ysl10 sex_2_10  c.eqincome10 c.popdens i.marital_bi ib2.empl3pt10 i.smok3_10 i.alcl3_10 i.walk4tm10 i.fq7prt10re_bi i.fq7veg10re_bi ib4.exam4_10re  i.gds5 ib1.bmi4_10 c.srh_4_10re i.dgns2ht10 i.dgns2dm10 i.dgns2hl10, cluster(scode)

reri logistic eve_sibou_cancer_6year SI_bi income_tertile_d2  age_ysl10 sex_2_10 i.edu2gp c.eqincome10 c.popdens i.marital_bi ib2.empl3pt10 i.smok3_10 i.alcl3_10 i.walk4tm10 i.fq7prt10re_bi i.fq7veg10re_bi ib4.exam4_10re  i.gds5 ib1.bmi4_10 c.srh_4_10re i.dgns2ht10 i.dgns2dm10 i.dgns2hl10 if income_tertile_d3==0, cluster(scode)

reri logistic eve_sibou_cancer_6year SI_bi income_tertile_d3  age_ysl10 sex_2_10 i.edu2gp c.eqincome10 c.popdens i.marital_bi ib2.empl3pt10 i.smok3_10 i.alcl3_10 i.walk4tm10 i.fq7prt10re_bi i.fq7veg10re_bi ib4.exam4_10re  i.gds5 ib1.bmi4_10 c.srh_4_10re i.dgns2ht10 i.dgns2dm10 i.dgns2hl10 if income_tertile_d2==0, cluster(scode)

reri logistic eve_sibou_cancer_6year SI_bi popdens_tertile_d2  age_ysl10 sex_2_10 i.edu2gp c.eqincome10 c.popdens i.marital_bi ib2.empl3pt10 i.smok3_10 i.alcl3_10 i.walk4tm10 i.fq7prt10re_bi i.fq7veg10re_bi ib4.exam4_10re  i.gds5 ib1.bmi4_10 c.srh_4_10re i.dgns2ht10 i.dgns2dm10 i.dgns2hl10 if popdens_tertile_d3==0, cluster(scode)

reri logistic eve_sibou_cancer_6year SI_bi popdens_tertile_d3  age_ysl10 sex_2_10 i.edu2gp c.eqincome10 c.popdens i.marital_bi ib2.empl3pt10 i.smok3_10 i.alcl3_10

© 2024 Nakagomi A et al. *JAMA Network Open.*

i.walk4tm10 i.fq7prt10re_bi i.fq7veg10re_bi ib4.exam4_10re  i.gds5 ib1.bmi4_10 c.srh_4_10re i.dgns2ht10 i.dgns2dm10 i.dgns2hl10 if popdens_tertile_d2==0, cluster(scode)

reri logistic eve_sibou_cancer_6year SI_bi marital_bi age_ysl10 sex_2_10 i.edu2gp c.eqincome10 c.popdens ib2.empl3pt10 i.smok3_10 i.alcl3_10 i.walk4tm10 i.fq7prt10re_bi i.fq7veg10re_bi ib4.exam4_10re  i.gds5 ib1.bmi4_10 c.srh_4_10re i.dgns2ht10 i.dgns2dm10 i.dgns2hl10, cluster(scode)

reri logistic eve_sibou_cancer_6year SI_bi empl3pt10_d1  age_ysl10 sex_2_10 i.edu2gp c.eqincome10 c.popdens i.marital_bi i.smok3_10 i.alcl3_10 i.walk4tm10 i.fq7prt10re_bi i.fq7veg10re_bi ib4.exam4_10re  i.gds5 ib1.bmi4_10 c.srh_4_10re i.dgns2ht10 i.dgns2dm10 i.dgns2hl10 if empl3pt10_d3==0, cluster(scode)

reri logistic eve_sibou_cancer_6year SI_bi empl3pt10_d3  age_ysl10 sex_2_10 i.edu2gp c.eqincome10 c.popdens i.marital_bi i.smok3_10 i.alcl3_10 i.walk4tm10 i.fq7prt10re_bi i.fq7veg10re_bi ib4.exam4_10re  i.gds5 ib1.bmi4_10 c.srh_4_10re i.dgns2ht10 i.dgns2dm10 i.dgns2hl10 if empl3pt10_d1==0, cluster(scode)

*subgroup

bysort age75: logistic eve_sibou_cancer_6year i.SI_bi age_ysl10 sex_2_10 i.edu2gp c.eqincome10 c.popdens i.marital_bi ib2.empl3pt10 i.smok3_10 i.alcl3_10 i.walk4tm10 i.fq7prt10re_bi i.fq7veg10re_bi ib4.exam4_10re  i.gds5 ib1.bmi4_10 c.srh_4_10re i.dgns2ht10 i.dgns2dm10 i.dgns2hl10, cluster(scode)

bysort sex_2_10: logistic eve_sibou_cancer_6year i.SI_bi age_ysl10 sex_2_10 i.edu2gp c.eqincome10 c.popdens i.marital_bi ib2.empl3pt10 i.smok3_10 i.alcl3_10 i.walk4tm10 i.fq7prt10re_bi i.fq7veg10re_bi ib4.exam4_10re  i.gds5 ib1.bmi4_10 c.srh_4_10re i.dgns2ht10 i.dgns2dm10 i.dgns2hl10, cluster(scode)

bysort edu2gp: logistic eve_sibou_cancer_6year i.SI_bi age_ysl10 sex_2_10 i.edu2gp c.eqincome10 c.popdens i.marital_bi ib2.empl3pt10 i.smok3_10 i.alcl3_10 i.walk4tm10 i.fq7prt10re_bi i.fq7veg10re_bi ib4.exam4_10re  i.gds5 ib1.bmi4_10 c.srh_4_10re i.dgns2ht10 i.dgns2dm10 i.dgns2hl10, cluster(scode)

bysort income_tertile: logistic eve_sibou_cancer_6year i.SI_bi age_ysl10 sex_2_10 i.edu2gp c.eqincome10 c.popdens i.marital_bi ib2.empl3pt10 i.smok3_10 i.alcl3_10 i.walk4tm10 i.fq7prt10re_bi i.fq7veg10re_bi ib4.exam4_10re  i.gds5 ib1.bmi4_10 c.srh_4_10re i.dgns2ht10 i.dgns2dm10 i.dgns2hl10, cluster(scode)

© 2024 Nakagomi A et al. *JAMA Network Open.*

bysort popdens_tertile: logistic eve_sibou_cancer_6year i.SI_bi age_ysl10 sex_2_10 i.edu2gp c.eqincome10 c.popdens i.marital_bi ib2.empl3pt10 i.smok3_10 i.alcl3_10 i.walk4tm10 i.fq7prt10re_bi i.fq7veg10re_bi ib4.exam4_10re i.gds5 ib1.bmi4_10 c.srh_4_10re i.dgns2ht10 i.dgns2dm10 i.dgns2hl10, cluster(scode)

bysort marital_bi: logistic eve_sibou_cancer_6year i.SI_bi age_ysl10 sex_2_10 i.edu2gp c.eqincome10 c.popdens i.marital_bi ib2.empl3pt10 i.smok3_10 i.alcl3_10 i.walk4tm10 i.fq7prt10re_bi i.fq7veg10re_bi ib4.exam4_10re i.gds5 ib1.bmi4_10 c.srh_4_10re i.dgns2ht10 i.dgns2dm10 i.dgns2hl10, cluster(scode)

bysort empl3pt10: logistic eve_sibou_cancer_6year i.SI_bi age_ysl10 sex_2_10 i.edu2gp c.eqincome10 c.popdens i.marital_bi ib2.empl3pt10 i.smok3_10 i.alcl3_10 i.walk4tm10 i.fq7prt10re_bi i.fq7veg10re_bi ib4.exam4_10re i.gds5 ib1.bmi4_10 c.srh_4_10re i.dgns2ht10 i.dgns2dm10 i.dgns2hl10, cluster(scode)

***********Cox regression analysis*****************

*All-cause mortality

stset days_sibou_lost2_6year, failure(eve_shibou17_6year ==1) id(samplenumber) scale(365.25)

*model 1

stcox i.SI_bi age_ysl10 sex_2_10, cluster(scode)

*model 2

stcox i.SI_bi age_ysl10 sex_2_10 i.edu2gp c.eqincome10 c.popdens i.marital_bi ib2.empl3pt10, cluster(scode)

*model 3

stcox i.SI_bi age_ysl10 sex_2_10 i.edu2gp c.eqincome10 c.popdens i.marital_bi ib2.empl3pt10 i.smok3_10 i.alcl3_10 i.walk4tm10 i.fq7prt10re_bi i.fq7veg10re_bi ib4.exam4_10re i.gds5 ib1.bmi4_10 c.srh_4_10re i.dgns2ht10 i.dgns2dm10 i.dgns2hl10, cluster(scode)

© 2024 Nakagomi A et al. *JAMA Network Open.*

*CVD mortality

*competing risk model

stset days_sibou_lost2_6year, failure(eve_sibou_IHD_stroke_6year==1) id(samplenumber) scale(365.25)

*model 1

stcrreg i.SI_bi age_ysl10 sex_2_10, compete(eve_sibou_IHD_stroke_6year_comp==2) cluster(scode)

*model 2

stcrreg i.SI_bi age_ysl10 sex_2_10 i.edu2gp c.eqincome10 c.popdens i.marital_bi ib2.empl3pt10, compete(eve_sibou_IHD_stroke_6year_comp==2) cluster(scode)

*model 3

stcrreg i.SI_bi age_ysl10 sex_2_10 i.edu2gp c.eqincome10 c.popdens i.marital_bi ib2.empl3pt10 i.smok3_10 i.alcl3_10 i.walk4tm10 i.fq7prt10re_bi i.fq7veg10re_bi ib4.exam4_10re  i.gds5 ib1.bmi4_10 c.srh_4_10re i.dgns2ht10 i.dgns2dm10 i.dgns2hl10, compete(eve_sibou_IHD_stroke_6year_comp==2) cluster(scode)

*cancer

*competing risk model

stset days_sibou_lost2_6year, failure(eve_sibou_cancer_6year==1) id(samplenumber) scale(365.25)

*model 1

stcrreg i.SI_bi age_ysl10 sex_2_10, compete(eve_sibou_cancer_6year_comp==2) cluster(scode)

*model 2

stcrreg i.SI_bi age_ysl10 sex_2_10 i.edu2gp c.eqincome10 c.popdens i.marital_bi ib2.empl3pt10, compete(eve_sibou_cancer_6year_comp==2) cluster(scode)

*model 3

stcrreg i.SI_bi age_ysl10 sex_2_10 i.edu2gp c.eqincome10 c.popdens i.marital_bi ib2.empl3pt10 i.smok3_10 i.alcl3_10 i.walk4tm10 i.fq7prt10re_bi i.fq7veg10re_bi ib4.exam4_10re  i.gds5 ib1.bmi4_10 c.srh_4_10re i.dgns2ht10 i.dgns2dm10 i.dgns2hl10, compete(eve_sibou_cancer_6year_comp==2) cluster(scode)

© 2024 Nakagomi A et al. *JAMA Network Open.*

*effect modification

*All-cause mortality

stset days_sibou_lost2_6year, failure(eve_shibou17_6year ==1) id(samplenumber) scale(365.25)

stcox i.SI_bi##i.age75 c.age_ysl10 sex_2_10 i.edu2gp c.eqincome10 c.popdens i.marital_bi ib2.empl3pt10 i.smok3_10 i.alcl3_10 i.walk4tm10 i.fq7prt10re_bi i.fq7veg10re_bi ib4.exam4_10re i.gds5 ib1.bmi4_10 c.srh_4_10re i.dgns2ht10 i.dgns2dm10 i.dgns2hl10, cluster(scode)

stcox i.SI_bi##i.sex_2_10 c.age_ysl10 sex_2_10 i.edu2gp c.eqincome10 c.popdens i.marital_bi ib2.empl3pt10 i.smok3_10 i.alcl3_10 i.walk4tm10 i.fq7prt10re_bi i.fq7veg10re_bi ib4.exam4_10re i.gds5 ib1.bmi4_10 c.srh_4_10re i.dgns2ht10 i.dgns2dm10 i.dgns2hl10, cluster(scode)

stcox i.SI_bi##i.edu2gp c.age_ysl10 sex_2_10 i.edu2gp c.eqincome10 c.popdens i.marital_bi ib2.empl3pt10 i.smok3_10 i.alcl3_10 i.walk4tm10 i.fq7prt10re_bi i.fq7veg10re_bi ib4.exam4_10re i.gds5 ib1.bmi4_10 c.srh_4_10re i.dgns2ht10 i.dgns2dm10 i.dgns2hl10, cluster(scode)

stcox i.SI_bi##i.income_tertile c.age_ysl10 sex_2_10 i.edu2gp c.eqincome10 c.popdens i.marital_bi ib2.empl3pt10 i.smok3_10 i.alcl3_10 i.walk4tm10 i.fq7prt10re_bi i.fq7veg10re_bi ib4.exam4_10re i.gds5 ib1.bmi4_10 c.srh_4_10re i.dgns2ht10 i.dgns2dm10 i.dgns2hl10, cluster(scode)

stcox i.SI_bi##i.popdens_tertile c.age_ysl10 sex_2_10 i.edu2gp c.eqincome10 c.popdens i.marital_bi ib2.empl3pt10 i.smok3_10 i.alcl3_10 i.walk4tm10 i.fq7prt10re_bi i.fq7veg10re_bi ib4.exam4_10re i.gds5 ib1.bmi4_10 c.srh_4_10re i.dgns2ht10 i.dgns2dm10 i.dgns2hl10, cluster(scode)

stcox i.SI_bi##i.marital_bi c.age_ysl10 sex_2_10 i.edu2gp c.eqincome10 c.popdens i.marital_bi ib2.empl3pt10 i.smok3_10 i.alcl3_10 i.walk4tm10 i.fq7prt10re_bi

© 2024 Nakagomi A et al. *JAMA Network Open.*

i.fq7veg10re_bi ib4.exam4_10re  i.gds5 ib1.bmi4_10 c.srh_4_10re i.dgns2ht10 i.dgns2dm10 i.dgns2hl10, cluster(scode)

stcox i.SI_bi##ib2.empl3pt10 c.age_ysl10 sex_2_10 i.edu2gp c.eqincome10 c.popdens i.marital_bi ib2.empl3pt10 i.smok3_10 i.alcl3_10 i.walk4tm10 i.fq7prt10re_bi i.fq7veg10re_bi ib4.exam4_10re  i.gds5 ib1.bmi4_10 c.srh_4_10re i.dgns2ht10 i.dgns2dm10 i.dgns2hl10, cluster(scode)

*RERI

reri stcox SI_bi age75 age_ysl10 sex_2_10 i.edu2gp c.eqincome10 c.popdens i.marital_bi ib2.empl3pt10 i.smok3_10 i.alcl3_10 i.walk4tm10 i.fq7prt10re_bi i.fq7veg10re_bi ib4.exam4_10re  i.gds5 ib1.bmi4_10 c.srh_4_10re i.dgns2ht10 i.dgns2dm10 i.dgns2hl10, cluster(scode)

reri stcox SI_bi sex_2_10 age_ysl10 i.edu2gp c.eqincome10 c.popdens i.marital_bi ib2.empl3pt10 i.smok3_10 i.alcl3_10 i.walk4tm10 i.fq7prt10re_bi i.fq7veg10re_bi ib4.exam4_10re  i.gds5 ib1.bmi4_10 c.srh_4_10re i.dgns2ht10 i.dgns2dm10 i.dgns2hl10, cluster(scode)

reri stcox SI_bi edu2gp age_ysl10 sex_2_10  c.eqincome10 c.popdens i.marital_bi ib2.empl3pt10 i.smok3_10 i.alcl3_10 i.walk4tm10 i.fq7prt10re_bi i.fq7veg10re_bi ib4.exam4_10re  i.gds5 ib1.bmi4_10 c.srh_4_10re i.dgns2ht10 i.dgns2dm10 i.dgns2hl10, cluster(scode)

reri stcox SI_bi income_tertile_d2  age_ysl10 sex_2_10 i.edu2gp c.eqincome10 c.popdens i.marital_bi ib2.empl3pt10 i.smok3_10 i.alcl3_10 i.walk4tm10 i.fq7prt10re_bi i.fq7veg10re_bi ib4.exam4_10re  i.gds5 ib1.bmi4_10 c.srh_4_10re i.dgns2ht10 i.dgns2dm10 i.dgns2hl10 if income_tertile_d3==0, cluster(scode)

reri stcox SI_bi income_tertile_d3  age_ysl10 sex_2_10 i.edu2gp c.eqincome10 c.popdens i.marital_bi ib2.empl3pt10 i.smok3_10 i.alcl3_10 i.walk4tm10 i.fq7prt10re_bi i.fq7veg10re_bi ib4.exam4_10re  i.gds5 ib1.bmi4_10 c.srh_4_10re i.dgns2ht10 i.dgns2dm10 i.dgns2hl10 if income_tertile_d2==0, cluster(scode)

reri stcox SI_bi popdens_tertile_d2  age_ysl10 sex_2_10 i.edu2gp c.eqincome10 c.popdens i.marital_bi ib2.empl3pt10 i.smok3_10 i.alcl3_10 i.walk4tm10 i.fq7prt10re_bi i.fq7veg10re_bi ib4.exam4_10re  i.gds5 ib1.bmi4_10 c.srh_4_10re i.dgns2ht10 i.dgns2dm10 i.dgns2hl10 if popdens_tertile_d3==0, cluster(scode)

reri stcox SI_bi popdens_tertile_d3  age_ysl10 sex_2_10 i.edu2gp c.eqincome10 c.popdens i.marital_bi ib2.empl3pt10 i.smok3_10 i.alcl3_10 i.walk4tm10 i.fq7prt10re_bi i.fq7veg10re_bi ib4.exam4_10re  i.gds5 ib1.bmi4_10 c.srh_4_10re i.dgns2ht10 i.dgns2dm10 i.dgns2hl10 if popdens_tertile_d2==0, cluster(scode)

© 2024 Nakagomi A et al. *JAMA Network Open.*

```
reri stcox SI_bi marital_bi age_ysl10 sex_2_10 i.edu2gp c.eqincome10 c.popdens ib2.empl3pt10 i.smok3_10 i.alcl3_10 i.walk4tm10 i.fq7prt10re_bi i.fq7veg10re_bi ib4.exam4_10re  i.gds5 ib1.bmi4_10 c.srh_4_10re i.dgns2ht10 i.dgns2dm10 i.dgns2hl10, cluster(scode)

reri stcox SI_bi empl3pt10_d1  age_ysl10 sex_2_10 i.edu2gp c.eqincome10 c.popdens i.marital_bi i.smok3_10 i.alcl3_10 i.walk4tm10 i.fq7prt10re_bi i.fq7veg10re_bi ib4.exam4_10re  i.gds5 ib1.bmi4_10 c.srh_4_10re i.dgns2ht10 i.dgns2dm10 i.dgns2hl10 if empl3pt10_d3==0, cluster(scode)

reri stcox SI_bi empl3pt10_d3  age_ysl10 sex_2_10 i.edu2gp c.eqincome10 c.popdens i.marital_bi i.smok3_10 i.alcl3_10 i.walk4tm10 i.fq7prt10re_bi i.fq7veg10re_bi ib4.exam4_10re  i.gds5 ib1.bmi4_10 c.srh_4_10re i.dgns2ht10 i.dgns2dm10 i.dgns2hl10 if empl3pt10_d1==0, cluster(scode)

*subgroup

bysort age75: stcox i.SI_bi age_ysl10 sex_2_10 i.edu2gp c.eqincome10 c.popdens i.marital_bi ib2.empl3pt10 i.smok3_10 i.alcl3_10 i.walk4tm10 i.fq7prt10re_bi i.fq7veg10re_bi ib4.exam4_10re  i.gds5 ib1.bmi4_10 c.srh_4_10re i.dgns2ht10 i.dgns2dm10 i.dgns2hl10, cluster(scode)

bysort sex_2_10: stcox i.SI_bi age_ysl10 sex_2_10 i.edu2gp c.eqincome10 c.popdens i.marital_bi ib2.empl3pt10 i.smok3_10 i.alcl3_10 i.walk4tm10 i.fq7prt10re_bi i.fq7veg10re_bi ib4.exam4_10re  i.gds5 ib1.bmi4_10 c.srh_4_10re i.dgns2ht10 i.dgns2dm10 i.dgns2hl10, cluster(scode)

bysort edu2gp: stcox i.SI_bi age_ysl10 sex_2_10 i.edu2gp c.eqincome10 c.popdens i.marital_bi ib2.empl3pt10 i.smok3_10 i.alcl3_10 i.walk4tm10 i.fq7prt10re_bi i.fq7veg10re_bi ib4.exam4_10re  i.gds5 ib1.bmi4_10 c.srh_4_10re i.dgns2ht10 i.dgns2dm10 i.dgns2hl10, cluster(scode)

bysort income_tertile: stcox i.SI_bi age_ysl10 sex_2_10 i.edu2gp c.eqincome10 c.popdens i.marital_bi ib2.empl3pt10 i.smok3_10 i.alcl3_10 i.walk4tm10 i.fq7prt10re_bi i.fq7veg10re_bi ib4.exam4_10re  i.gds5 ib1.bmi4_10 c.srh_4_10re i.dgns2ht10 i.dgns2dm10 i.dgns2hl10, cluster(scode)
```

© 2024 Nakagomi A et al. *JAMA Network Open.*

```
bysort popdens_tertile: stcox i.SI_bi age_ysl10 sex_2_10 i.edu2gp c.eqincome10
c.popdens i.marital_bi ib2.empl3pt10 i.smok3_10 i.alcl3_10 i.walk4tm10
i.fq7prt10re_bi i.fq7veg10re_bi ib4.exam4_10re  i.gds5 ib1.bmi4_10 c.srh_4_10re
i.dgns2ht10 i.dgns2dm10 i.dgns2hl10, cluster(scode)

bysort marital_bi: stcox i.SI_bi age_ysl10 sex_2_10 i.edu2gp c.eqincome10 c.popdens
i.marital_bi ib2.empl3pt10 i.smok3_10 i.alcl3_10 i.walk4tm10 i.fq7prt10re_bi
i.fq7veg10re_bi ib4.exam4_10re  i.gds5 ib1.bmi4_10 c.srh_4_10re i.dgns2ht10
i.dgns2dm10 i.dgns2hl10, cluster(scode)

bysort empl3pt10: stcox i.SI_bi age_ysl10 sex_2_10 i.edu2gp c.eqincome10 c.popdens
i.marital_bi ib2.empl3pt10 i.smok3_10 i.alcl3_10 i.walk4tm10 i.fq7prt10re_bi
i.fq7veg10re_bi ib4.exam4_10re  i.gds5 ib1.bmi4_10 c.srh_4_10re i.dgns2ht10
i.dgns2dm10 i.dgns2hl10, cluster(scode)

*CVD

stset days_sibou_lost2_6year, failure(eve_sibou_IHD_stroke_6year==1)
id(samplenumber) scale(365.25)

*effect modification (additive and multiplicative)

*age

gen g = SI_bi

gen e = age75

gen Ige = g*e

stcrreg g e Ige age_ysl10 sex_2_10 i.edu2gp c.eqincome10 c.popdens i.marital_bi
ib2.empl3pt10 i.smok3_10 i.alcl3_10 i.walk4tm10 i.fq7prt10re_bi i.fq7veg10re_bi
ib4.exam4_10re  i.gds5 ib1.bmi4_10 c.srh_4_10re i.dgns2ht10 i.dgns2dm10
i.dgns2hl10, compete(eve_sibou_IHD_stroke_6year_comp==2) cluster(scode)

nlcom exp(_b[g]+_b[e]+_b[Ige])-exp(_b[g])-exp(_b[e])+1

*gender

drop g e Ige
```

© 2024 Nakagomi A et al. *JAMA Network Open.*

```
gen g = SI_bi

gen e = sex_2_10

gen Ige = g*e

stcrreg g e Ige age_ysl10 i.edu2gp c.eqincome10 c.popdens i.marital_bi ib2.empl3pt10
i.smok3_10 i.alcl3_10 i.walk4tm10 i.fq7prt10re_bi i.fq7veg10re_bi ib4.exam4_10re
i.gds5 ib1.bmi4_10 c.srh_4_10re i.dgns2ht10 i.dgns2dm10 i.dgns2hl10,
compete(eve_sibou_IHD_stroke_6year_comp==2) cluster(scode)

nlcom exp(_b[g]+_b[e]+_b[Ige])-exp(_b[g])-exp(_b[e])+1


*education

drop g e Ige

gen g = SI_bi

gen e = edu2gp

gen Ige = g*e

stcrreg g e Ige age_ysl10 i.sex_2_10 c.eqincome10 c.popdens i.marital_bi
ib2.empl3pt10 i.smok3_10 i.alcl3_10 i.walk4tm10 i.fq7prt10re_bi i.fq7veg10re_bi
ib4.exam4_10re  i.gds5 ib1.bmi4_10 c.srh_4_10re i.dgns2ht10 i.dgns2dm10
i.dgns2hl10, compete(eve_sibou_IHD_stroke_6year_comp==2) cluster(scode)

nlcom exp(_b[g]+_b[e]+_b[Ige])-exp(_b[g])-exp(_b[e])+1


*income

drop g e Ige

gen g = SI_bi

gen e = income_tertile_d2

gen Ige = g*e

stcrreg g e Ige age_ysl10 i.sex_2_10 i.edu2gp c.eqincome10 c.popdens i.marital_bi
ib2.empl3pt10 i.smok3_10 i.alcl3_10 i.walk4tm10 i.fq7prt10re_bi i.fq7veg10re_bi
ib4.exam4_10re  i.gds5 ib1.bmi4_10 c.srh_4_10re i.dgns2ht10 i.dgns2dm10
i.dgns2hl10 if income_tertile_d3==0,
compete(eve_sibou_IHD_stroke_6year_comp==2) cluster(scode)

nlcom exp(_b[g]+_b[e]+_b[Ige])-exp(_b[g])-exp(_b[e])+1
```

© 2024 Nakagomi A et al. *JAMA Network Open.*

```
drop g e Ige

gen g = SI_bi

gen e = income_tertile_d3

gen Ige = g*e

stcrreg g e Ige age_ysl10 i.sex_2_10 i.edu2gp c.eqincome10 c.popdens i.marital_bi
ib2.empl3pt10 i.smok3_10 i.alcl3_10 i.walk4tm10 i.fq7prt10re_bi i.fq7veg10re_bi
ib4.exam4_10re  i.gds5 ib1.bmi4_10 c.srh_4_10re i.dgns2ht10 i.dgns2dm10
i.dgns2hl10 if income_tertile_d2==0,
compete(eve_sibou_IHD_stroke_6year_comp==2) cluster(scode)

nlcom exp(_b[g]+_b[e]+_b[Ige])-exp(_b[g])-exp(_b[e])+1



*popdens

drop g e Ige

gen g = SI_bi

gen e = popdens_tertile_d2

gen Ige = g*e

stcrreg g e Ige age_ysl10 i.sex_2_10 i.edu2gp c.eqincome10 c.popdens i.marital_bi
ib2.empl3pt10 i.smok3_10 i.alcl3_10 i.walk4tm10 i.fq7prt10re_bi i.fq7veg10re_bi
ib4.exam4_10re  i.gds5 ib1.bmi4_10 c.srh_4_10re i.dgns2ht10 i.dgns2dm10
i.dgns2hl10 if popdens_tertile_d3==0,
compete(eve_sibou_IHD_stroke_6year_comp==2) cluster(scode)

nlcom exp(_b[g]+_b[e]+_b[Ige])-exp(_b[g])-exp(_b[e])+1


drop g e Ige

gen g = SI_bi

gen e = popdens_tertile_d3

gen Ige = g*e

stcrreg g e Ige age_ysl10 i.sex_2_10 i.edu2gp c.eqincome10 c.popdens i.marital_bi
ib2.empl3pt10 i.smok3_10 i.alcl3_10 i.walk4tm10 i.fq7prt10re_bi i.fq7veg10re_bi
ib4.exam4_10re  i.gds5 ib1.bmi4_10 c.srh_4_10re i.dgns2ht10 i.dgns2dm10
i.dgns2hl10 if popdens_tertile_d2==0, compete(eve_sibou_IHD_stroke_competing==2)
cluster(scode)
```

© 2024 Nakagomi A et al. *JAMA Network Open.*

```
nlcom exp(_b[g]+_b[e]+_b[Ige])-exp(_b[g])-exp(_b[e])+1


*marital status

drop g e Ige

gen g = SI_bi

gen e = marital_bi

gen Ige = g*e

stcrreg g e Ige age_ysl10 i.sex_2_10 i.edu2gp c.eqincome10 c.popdens ib2.empl3pt10 i.smok3_10 i.alcl3_10 i.walk4tm10 i.fq7prt10re_bi i.fq7veg10re_bi ib4.exam4_10re i.gds5 ib1.bmi4_10 c.srh_4_10re i.dgns2ht10 i.dgns2dm10 i.dgns2hl10, compete(eve_sibou_IHD_stroke_6year_comp==2) cluster(scode)

nlcom exp(_b[g]+_b[e]+_b[Ige])-exp(_b[g])-exp(_b[e])+1


*employment

drop g e Ige

gen g = SI_bi

gen e = empl3pt10_d1

gen Ige = g*e

stcrreg g e Ige age_ysl10 i.sex_2_10 i.edu2gp c.eqincome10 c.popdens i.marital_bi ib2.empl3pt10 i.smok3_10 i.alcl3_10 i.walk4tm10 i.fq7prt10re_bi i.fq7veg10re_bi ib4.exam4_10re  i.gds5 ib1.bmi4_10 c.srh_4_10re i.dgns2ht10 i.dgns2dm10 i.dgns2hl10 if empl3pt10_d3==0, compete(eve_sibou_IHD_stroke_6year_comp==2) cluster(scode)

nlcom exp(_b[g]+_b[e]+_b[Ige])-exp(_b[g])-exp(_b[e])+1


drop g e Ige

gen g = SI_bi

gen e = empl3pt10_d3

gen Ige = g*e
```

© 2024 Nakagomi A et al. *JAMA Network Open.*

```
stcrreg g e Ige age_ysl10 i.sex_2_10 i.edu2gp c.eqincome10 c.popdens i.marital_bi ib2.empl3pt10 i.smok3_10 i.alcl3_10 i.walk4tm10 i.fq7prt10re_bi i.fq7veg10re_bi ib4.exam4_10re  i.gds5 ib1.bmi4_10 c.srh_4_10re i.dgns2ht10 i.dgns2dm10 i.dgns2hl10 if empl3pt10_d1==0, compete(eve_sibou_IHD_stroke_competing==2) cluster(scode)

nlcom exp(_b[g]+_b[e]+_b[Ige])-exp(_b[g])-exp(_b[e])+1

*subgroup

bysort age75: stcrreg i.SI_bi age_ysl10 sex_2_10 i.edu2gp c.eqincome10 c.popdens i.marital_bi ib2.empl3pt10 i.smok3_10 i.alcl3_10 i.walk4tm10 i.fq7prt10re_bi i.fq7veg10re_bi ib4.exam4_10re  i.gds5 ib1.bmi4_10 c.srh_4_10re i.dgns2ht10 i.dgns2dm10 i.dgns2hl10, compete(eve_sibou_IHD_stroke_6year_comp==2) cluster(scode)

bysort sex_2_10: stcrreg i.SI_bi age_ysl10 sex_2_10 i.edu2gp c.eqincome10 c.popdens i.marital_bi ib2.empl3pt10 i.smok3_10 i.alcl3_10 i.walk4tm10 i.fq7prt10re_bi i.fq7veg10re_bi ib4.exam4_10re  i.gds5 ib1.bmi4_10 c.srh_4_10re i.dgns2ht10 i.dgns2dm10 i.dgns2hl10, compete(eve_sibou_IHD_stroke_6year_comp==2) cluster(scode)

bysort edu2gp: stcrreg i.SI_bi age_ysl10 sex_2_10 i.edu2gp c.eqincome10 c.popdens i.marital_bi ib2.empl3pt10 i.smok3_10 i.alcl3_10 i.walk4tm10 i.fq7prt10re_bi i.fq7veg10re_bi ib4.exam4_10re  i.gds5 ib1.bmi4_10 c.srh_4_10re i.dgns2ht10 i.dgns2dm10 i.dgns2hl10, compete(eve_sibou_IHD_stroke_6year_comp==2) cluster(scode)

bysort income_tertile: stcrreg i.SI_bi age_ysl10 sex_2_10 i.edu2gp c.eqincome10 c.popdens i.marital_bi ib2.empl3pt10 i.smok3_10 i.alcl3_10 i.walk4tm10 i.fq7prt10re_bi i.fq7veg10re_bi ib4.exam4_10re  i.gds5 ib1.bmi4_10 c.srh_4_10re i.dgns2ht10 i.dgns2dm10 i.dgns2hl10, compete(eve_sibou_IHD_stroke_6year_comp==2) cluster(scode)

bysort popdens_tertile: stcrreg i.SI_bi age_ysl10 sex_2_10 i.edu2gp c.eqincome10 c.popdens i.marital_bi ib2.empl3pt10 i.smok3_10 i.alcl3_10 i.walk4tm10 i.fq7prt10re_bi i.fq7veg10re_bi ib4.exam4_10re  i.gds5 ib1.bmi4_10 c.srh_4_10re
```

i.dgns2ht10 i.dgns2dm10 i.dgns2hl10, compete(eve_sibou_IHD_stroke_6year_comp==2) cluster(scode)

bysort marital_bi: stcrreg i.SI_bi age_ysl10 sex_2_10 i.edu2gp c.eqincome10 c.popdens i.marital_bi ib2.empl3pt10 i.smok3_10 i.alcl3_10 i.walk4tm10 i.fq7prt10re_bi i.fq7veg10re_bi ib4.exam4_10re i.gds5 ib1.bmi4_10 c.srh_4_10re i.dgns2ht10 i.dgns2dm10 i.dgns2hl10, compete(eve_sibou_IHD_stroke_6year_comp==2) cluster(scode)

bysort empl3pt10: stcrreg i.SI_bi age_ysl10 sex_2_10 i.edu2gp c.eqincome10 c.popdens i.marital_bi ib2.empl3pt10 i.smok3_10 i.alcl3_10 i.walk4tm10 i.fq7prt10re_bi i.fq7veg10re_bi ib4.exam4_10re i.gds5 ib1.bmi4_10 c.srh_4_10re i.dgns2ht10 i.dgns2dm10 i.dgns2hl10, compete(eve_sibou_IHD_stroke_6year_comp==2) cluster(scode)

*cancer

stset days_sibou_lost2_6year, failure(eve_sibou_cancer_6year==1) id(samplenumber) scale(365.25)

*effect modification (additive and multiplicative)

*age

drop g e Ige

gen g = SI_bi

gen e = age75

gen Ige = g*e

stcrreg g e Ige age_ysl10 sex_2_10 i.edu2gp c.eqincome10 c.popdens i.marital_bi ib2.empl3pt10 i.smok3_10 i.alcl3_10 i.walk4tm10 i.fq7prt10re_bi i.fq7veg10re_bi ib4.exam4_10re i.gds5 ib1.bmi4_10 c.srh_4_10re i.dgns2ht10 i.dgns2dm10 i.dgns2hl10, compete(eve_sibou_cancer_6year_comp==2) cluster(scode)

nlcom exp(_b[g]+_b[e]+_b[Ige])-exp(_b[g])-exp(_b[e])+1

© 2024 Nakagomi A et al. *JAMA Network Open.*

```
*gender

drop g e Ige

gen g = SI_bi

gen e = sex_2_10

gen Ige = g*e

stcrreg g e Ige age_ysl10 i.edu2gp c.eqincome10 c.popdens i.marital_bi ib2.empl3pt10
i.smok3_10 i.alcl3_10 i.walk4tm10 i.fq7prt10re_bi i.fq7veg10re_bi ib4.exam4_10re
i.gds5 ib1.bmi4_10 c.srh_4_10re i.dgns2ht10 i.dgns2dm10 i.dgns2hl10,
compete(eve_sibou_cancer_6year_comp==2) cluster(scode)

nlcom exp(_b[g]+_b[e]+_b[Ige])-exp(_b[g])-exp(_b[e])+1


*education

drop g e Ige

gen g = SI_bi

gen e = edu2gp

gen Ige = g*e

stcrreg g e Ige age_ysl10 i.sex_2_10 c.eqincome10 c.popdens i.marital_bi
ib2.empl3pt10 i.smok3_10 i.alcl3_10 i.walk4tm10 i.fq7prt10re_bi i.fq7veg10re_bi
ib4.exam4_10re  i.gds5 ib1.bmi4_10 c.srh_4_10re i.dgns2ht10 i.dgns2dm10
i.dgns2hl10, compete(eve_sibou_cancer_6year_comp==2) cluster(scode)

nlcom exp(_b[g]+_b[e]+_b[Ige])-exp(_b[g])-exp(_b[e])+1


*income

drop g e Ige

gen g = SI_bi

gen e = income_tertile_d2

gen Ige = g*e

stcrreg g e Ige age_ysl10 i.sex_2_10 i.edu2gp c.eqincome10 c.popdens i.marital_bi
ib2.empl3pt10 i.smok3_10 i.alcl3_10 i.walk4tm10 i.fq7prt10re_bi i.fq7veg10re_bi
ib4.exam4_10re  i.gds5 ib1.bmi4_10 c.srh_4_10re i.dgns2ht10 i.dgns2dm10
i.dgns2hl10 if income_tertile_d3==0, compete(eve_sibou_cancer_6year_comp==2)
cluster(scode)
```

© 2024 Nakagomi A et al. *JAMA Network Open.*

```
nlcom exp(_b[g]+_b[e]+_b[Ige])-exp(_b[g])-exp(_b[e])+1


drop g e Ige

gen g = SI_bi

gen e = income_tertile_d3

gen Ige = g*e

stcrreg g e Ige age_ysl10 i.sex_2_10 i.edu2gp c.eqincome10 c.popdens i.marital_bi ib2.empl3pt10 i.smok3_10 i.alcl3_10 i.walk4tm10 i.fq7prt10re_bi i.fq7veg10re_bi ib4.exam4_10re  i.gds5 ib1.bmi4_10 c.srh_4_10re i.dgns2ht10 i.dgns2dm10 i.dgns2hl10 if income_tertile_d2==0, compete(eve_sibou_cancer_6year_comp==2) cluster(scode)

nlcom exp(_b[g]+_b[e]+_b[Ige])-exp(_b[g])-exp(_b[e])+1



*popdens

drop g e Ige

gen g = SI_bi

gen e = popdens_tertile_d2

gen Ige = g*e

stcrreg g e Ige age_ysl10 i.sex_2_10 i.edu2gp c.eqincome10 c.popdens i.marital_bi ib2.empl3pt10 i.smok3_10 i.alcl3_10 i.walk4tm10 i.fq7prt10re_bi i.fq7veg10re_bi ib4.exam4_10re  i.gds5 ib1.bmi4_10 c.srh_4_10re i.dgns2ht10 i.dgns2dm10 i.dgns2hl10 if popdens_tertile_d3==0, compete(eve_sibou_cancer_6year_comp==2) cluster(scode)

nlcom exp(_b[g]+_b[e]+_b[Ige])-exp(_b[g])-exp(_b[e])+1


drop g e Ige

gen g = SI_bi

gen e = popdens_tertile_d3

gen Ige = g*e

stcrreg g e Ige age_ysl10 i.sex_2_10 i.edu2gp c.eqincome10 c.popdens i.marital_bi ib2.empl3pt10 i.smok3_10 i.alcl3_10 i.walk4tm10 i.fq7prt10re_bi i.fq7veg10re_bi
```

© 2024 Nakagomi A et al. *JAMA Network Open.*

```
ib4.exam4_10re  i.gds5 ib1.bmi4_10 c.srh_4_10re i.dgns2ht10 i.dgns2dm10
i.dgns2hl10 if popdens_tertile_d2==0, compete(eve_sibou_cancer_6year_comp==2)
cluster(scode)

nlcom exp(_b[g]+_b[e]+_b[Ige])-exp(_b[g])-exp(_b[e])+1


*marital status

drop g e Ige

gen g = SI_bi

gen e = marital_bi

gen Ige = g*e

stcrreg g e Ige age_ysl10 i.sex_2_10 i.edu2gp c.eqincome10 c.popdens ib2.empl3pt10
i.smok3_10 i.alcl3_10 i.walk4tm10 i.fq7prt10re_bi i.fq7veg10re_bi ib4.exam4_10re
i.gds5 ib1.bmi4_10 c.srh_4_10re i.dgns2ht10 i.dgns2dm10 i.dgns2hl10,
compete(eve_sibou_cancer_6year_comp==2) cluster(scode)

nlcom exp(_b[g]+_b[e]+_b[Ige])-exp(_b[g])-exp(_b[e])+1


*employment

drop g e Ige

gen g = SI_bi

gen e = empl3pt10_d1

gen Ige = g*e

stcrreg g e Ige age_ysl10 i.sex_2_10 i.edu2gp c.eqincome10 c.popdens i.marital_bi
ib2.empl3pt10 i.smok3_10 i.alcl3_10 i.walk4tm10 i.fq7prt10re_bi i.fq7veg10re_bi
ib4.exam4_10re  i.gds5 ib1.bmi4_10 c.srh_4_10re i.dgns2ht10 i.dgns2dm10
i.dgns2hl10 if empl3pt10_d3==0, compete(eve_sibou_cancer_6year_comp==2)
cluster(scode)

nlcom exp(_b[g]+_b[e]+_b[Ige])-exp(_b[g])-exp(_b[e])+1


drop g e Ige

gen g = SI_bi

gen e = empl3pt10_d3
```

© 2024 Nakagomi A et al. *JAMA Network Open.*

```
gen Ige = g*e

stcrreg g e Ige age_ysl10 i.sex_2_10 i.edu2gp c.eqincome10 c.popdens i.marital_bi ib2.empl3pt10 i.smok3_10 i.alcl3_10 i.walk4tm10 i.fq7prt10re_bi i.fq7veg10re_bi ib4.exam4_10re  i.gds5 ib1.bmi4_10 c.srh_4_10re i.dgns2ht10 i.dgns2dm10 i.dgns2hl10 if empl3pt10_d1==0, compete(eve_sibou_cancer_6year_comp==2) cluster(scode)

nlcom exp(_b[g]+_b[e]+_b[Ige])-exp(_b[g])-exp(_b[e])+1


*subgroup

bysort age75: stcrreg i.SI_bi age_ysl10 sex_2_10 i.edu2gp c.eqincome10 c.popdens i.marital_bi ib2.empl3pt10 i.smok3_10 i.alcl3_10 i.walk4tm10 i.fq7prt10re_bi i.fq7veg10re_bi ib4.exam4_10re  i.gds5 ib1.bmi4_10 c.srh_4_10re i.dgns2ht10 i.dgns2dm10 i.dgns2hl10, compete(eve_sibou_cancer_6year_comp==2) cluster(scode)

bysort sex_2_10: stcrreg i.SI_bi age_ysl10 sex_2_10 i.edu2gp c.eqincome10 c.popdens i.marital_bi ib2.empl3pt10 i.smok3_10 i.alcl3_10 i.walk4tm10 i.fq7prt10re_bi i.fq7veg10re_bi ib4.exam4_10re  i.gds5 ib1.bmi4_10 c.srh_4_10re i.dgns2ht10 i.dgns2dm10 i.dgns2hl10, compete(eve_sibou_cancer_6year_comp==2) cluster(scode)

bysort edu2gp: stcrreg i.SI_bi age_ysl10 sex_2_10 i.edu2gp c.eqincome10 c.popdens i.marital_bi ib2.empl3pt10 i.smok3_10 i.alcl3_10 i.walk4tm10 i.fq7prt10re_bi i.fq7veg10re_bi ib4.exam4_10re  i.gds5 ib1.bmi4_10 c.srh_4_10re i.dgns2ht10 i.dgns2dm10 i.dgns2hl10, compete(eve_sibou_cancer_6year_comp==2) cluster(scode)

bysort income_tertile: stcrreg i.SI_bi age_ysl10 sex_2_10 i.edu2gp c.eqincome10 c.popdens i.marital_bi ib2.empl3pt10 i.smok3_10 i.alcl3_10 i.walk4tm10 i.fq7prt10re_bi i.fq7veg10re_bi ib4.exam4_10re  i.gds5 ib1.bmi4_10 c.srh_4_10re i.dgns2ht10 i.dgns2dm10 i.dgns2hl10, compete(eve_sibou_cancer_6year_comp==2) cluster(scode)

bysort popdens_tertile: stcrreg i.SI_bi age_ysl10 sex_2_10 i.edu2gp c.eqincome10 c.popdens i.marital_bi ib2.empl3pt10 i.smok3_10 i.alcl3_10 i.walk4tm10 i.fq7prt10re_bi i.fq7veg10re_bi ib4.exam4_10re  i.gds5 ib1.bmi4_10 c.srh_4_10re i.dgns2ht10 i.dgns2dm10 i.dgns2hl10, compete(eve_sibou_cancer_6year_comp==2) cluster(scode)
```

© 2024 Nakagomi A et al. *JAMA Network Open.*

```
bysort marital_bi: stcrreg i.SI_bi age_ysl10 sex_2_10 i.edu2gp c.eqincome10 c.popdens
i.marital_bi ib2.empl3pt10 i.smok3_10 i.alcl3_10 i.walk4tm10 i.fq7prt10re_bi
i.fq7veg10re_bi ib4.exam4_10re  i.gds5 ib1.bmi4_10 c.srh_4_10re i.dgns2ht10
i.dgns2dm10 i.dgns2hl10, compete(eve_sibou_cancer_6year_comp==2) cluster(scode)
```

```
bysort empl3pt10: stcrreg i.SI_bi age_ysl10 sex_2_10 i.edu2gp c.eqincome10
c.popdens i.marital_bi ib2.empl3pt10 i.smok3_10 i.alcl3_10 i.walk4tm10
i.fq7prt10re_bi i.fq7veg10re_bi ib4.exam4_10re  i.gds5 ib1.bmi4_10 c.srh_4_10re
i.dgns2ht10 i.dgns2dm10 i.dgns2hl10, compete(eve_sibou_cancer_6year_comp==2)
cluster(scode)
```

© 2024 Nakagomi A et al. *JAMA Network Open.*

**eFigure 1.** Odds Ratios of Social Isolation for All-Cause Mortality, Cardiovascular Diseases Mortality, and Neoplasms Mortality Among Older People in Japan

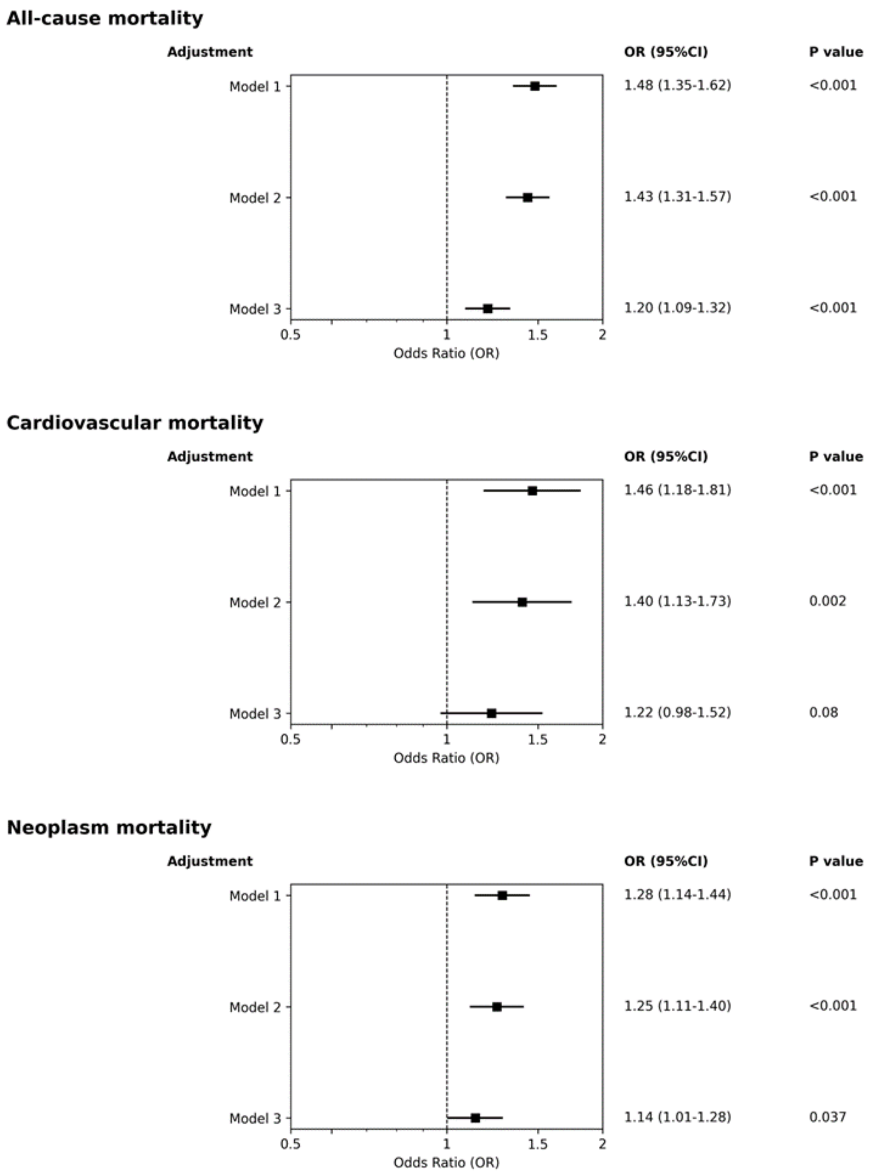


In model 1, age and gender were included.

© 2024 Nakagomi A et al. *JAMA Network Open.*

In model 2, socioeconomic status (education, equivalized income, population density), marital status, and employment status were included.

In model 3, health behaviours (smoking, drinking alcohol, walking, frequency of eating meat and fish, frequency of eating fruits and vegetables, taking health check-ups), psychological factors (depressive symptoms), and physiological factors (body mass index, self-rated health, hypertension, diabetes, dyslidemia) were included.

OR: odds ratio; CI: confidence interval.

© 2024 Nakagomi A et al. *JAMA Network Open.*

**eFigure 2.** Odds Ratios of Social Isolation for All-Cause Mortality Over Levels of Age at Baseline

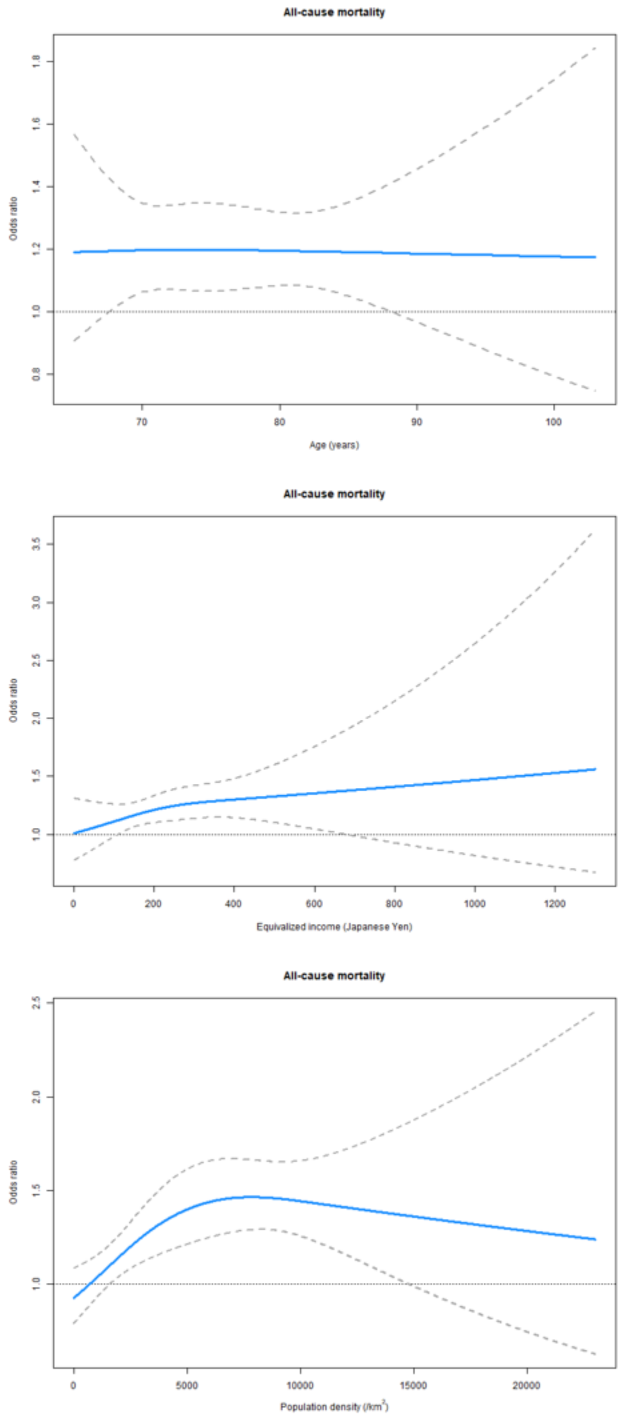


© 2024 Nakagomi A et al. *JAMA Network Open.*

**eFigure 3.** Odds Ratios of Social Isolation for Cardiovascular Diseases Mortality Over Levels of Age at Baseline

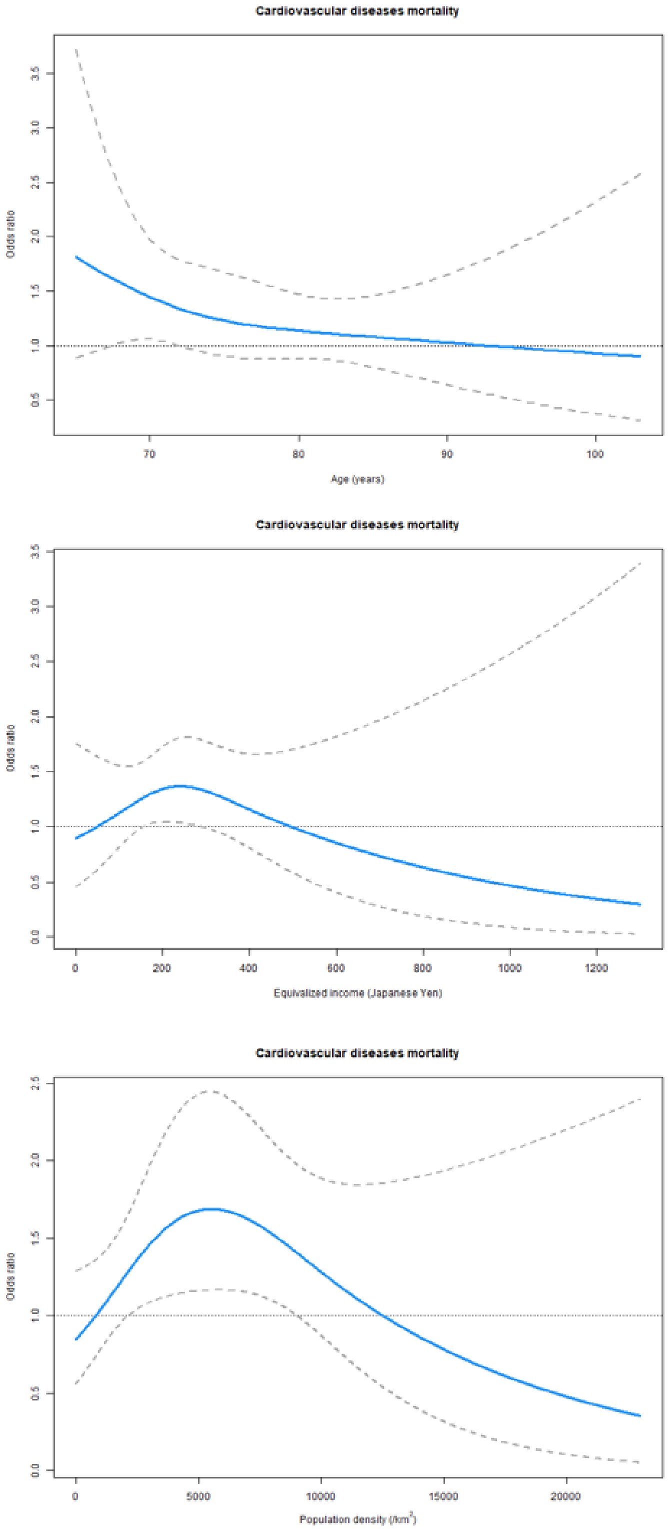


© 2024 Nakagomi A et al. *JAMA Network Open.*

**eFigure 4.** Odds Ratios of Social Isolation for Neoplasms Mortality Over Levels of Age at Baseline

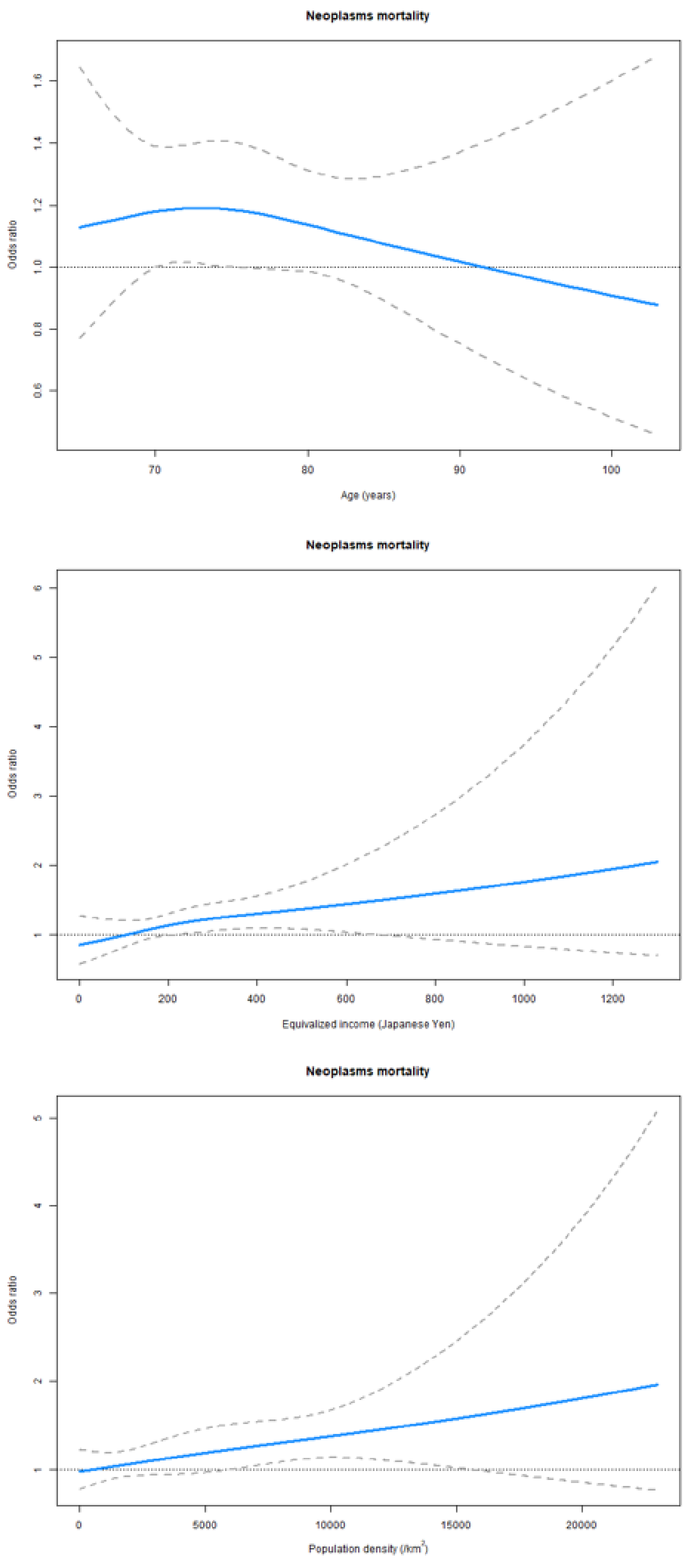


© 2024 Nakagomi A et al. *JAMA Network Open.*

**eFigure 5.** Odds Ratios of Social Isolation for All-Cause Mortality Within All Participants and Subgroups of Moderators: Using Population Density That Excluded Nondeveloped Areas (eg, Rivers, Lakes, Forests, and Wasteland) as Well as Nonresidential Land (Such as Farms and Industrial Districts)

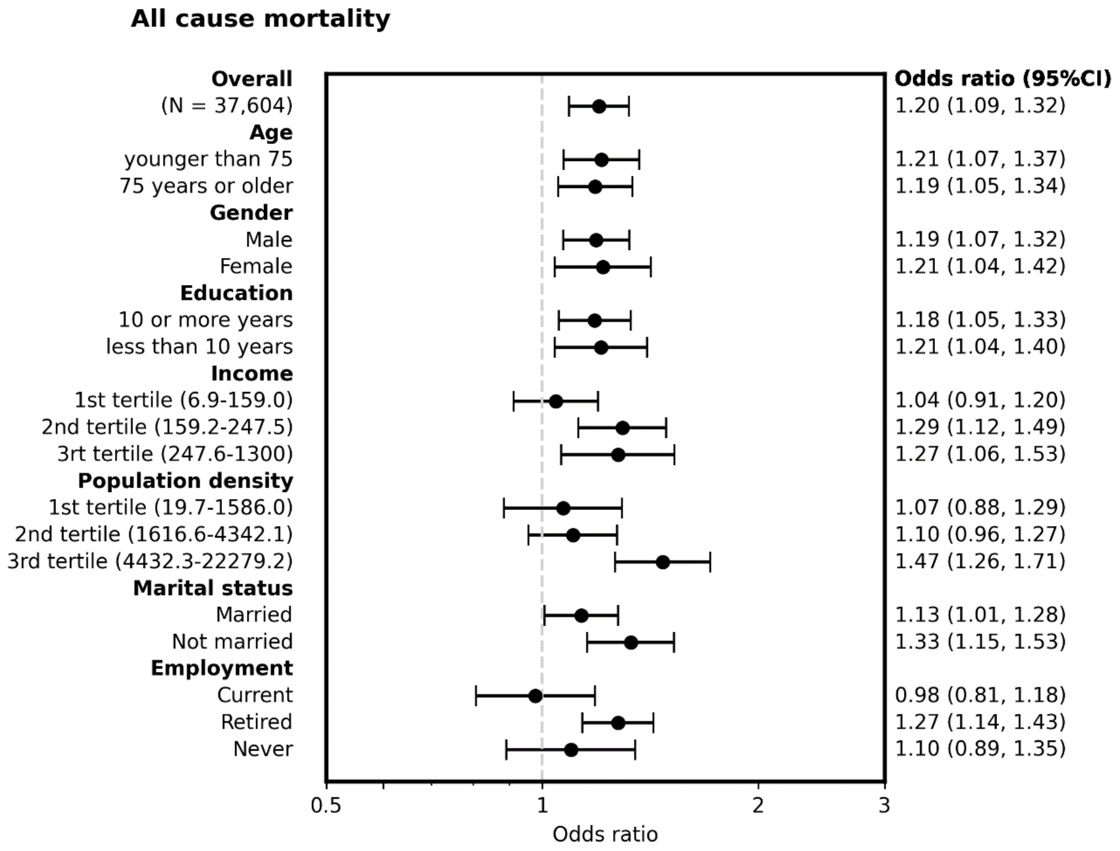


© 2024 Nakagomi A et al. *JAMA Network Open.*

**eFigure 6.** Odds Ratios of Social Isolation for Cardiovascular Diseases Mortality Within All Participants and Subgroups of Moderators: Using Population Density That Excluded Nondeveloped Areas (eg, Rivers, Lakes, Forests, and Wasteland) as Well as Nonresidential Land (Such as Farms and Industrial Districts)

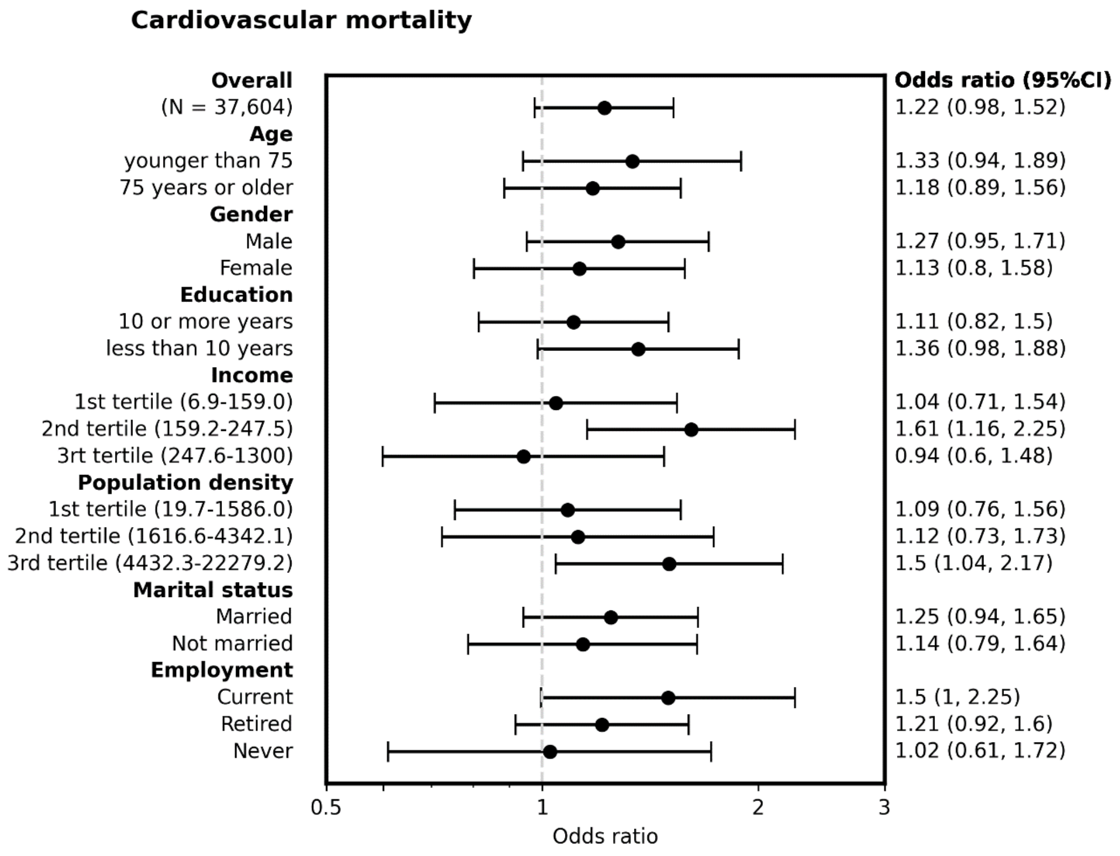


© 2024 Nakagomi A et al. *JAMA Network Open.*

**eFigure 7.** Odds Ratios of Social Isolation for Neoplasm Diseases Mortality Within All Participants and Subgroups of Moderators: Using Population Density That Excluded Nondeveloped Areas (eg, Rivers, Lakes, Forests, and Wasteland) as Well as Nonresidential Land (Such as Farms and Industrial Districts)

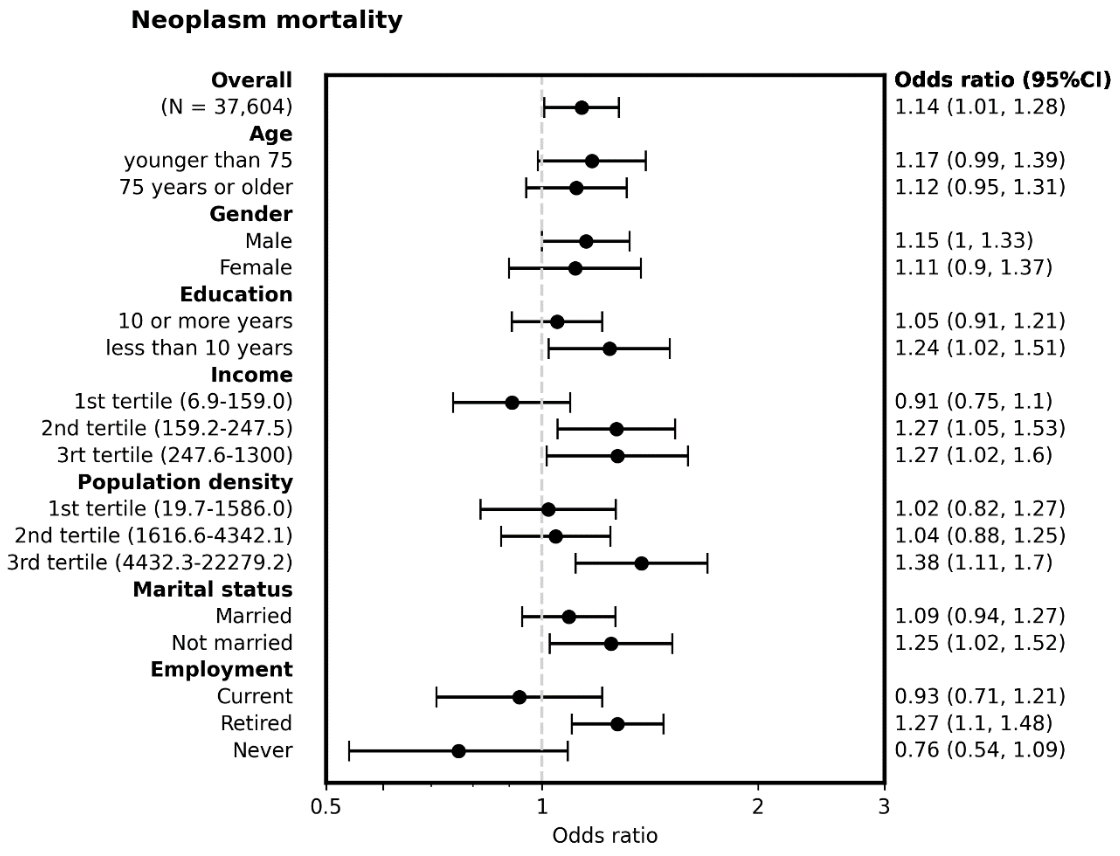


© 2024 Nakagomi A et al. *JAMA Network Open.*

**eFigure 8.** Odds Ratios of Social Isolation for All-Cause Mortality Within All Participants and Subgroups of Moderators: Clustered at Municipal Levels

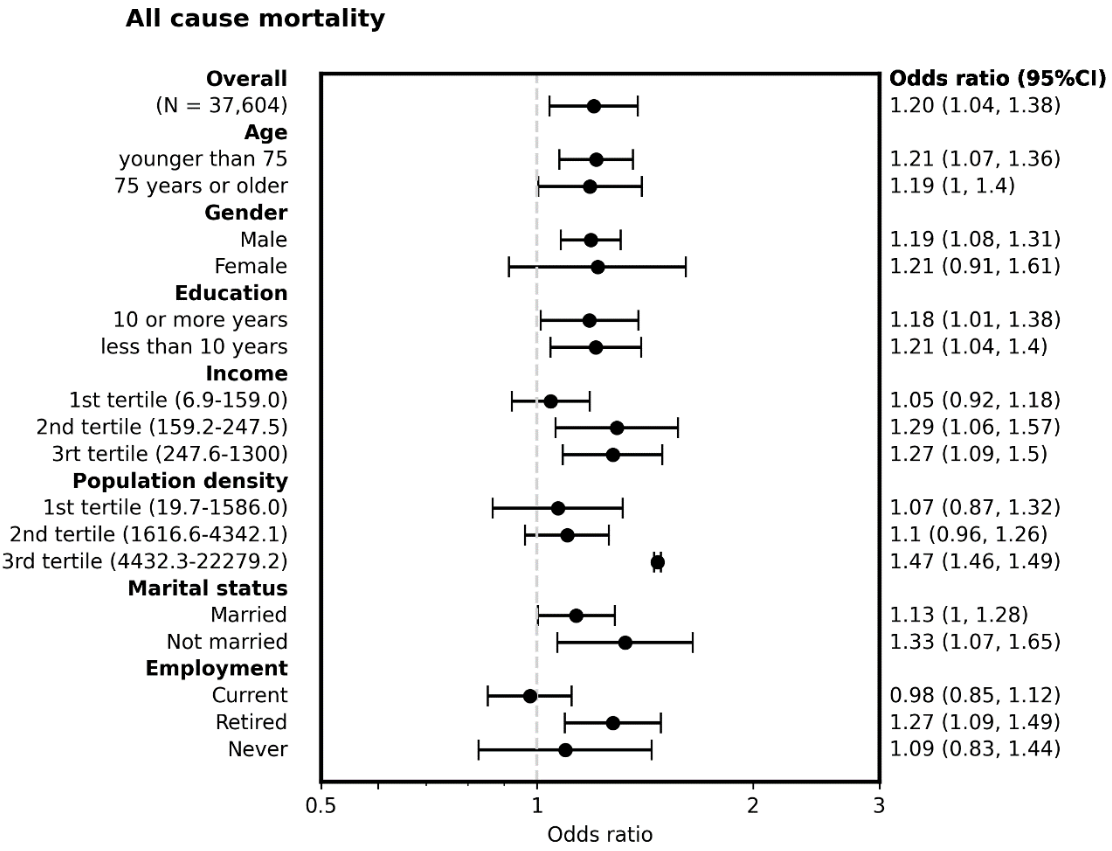


© 2024 Nakagomi A et al. *JAMA Network Open.*

**eFigure 9.** Odds Ratios of Social Isolation for Cardiovascular Disease Mortality Within All Participants and Subgroups of Moderators: Clustered at Municipal Levels

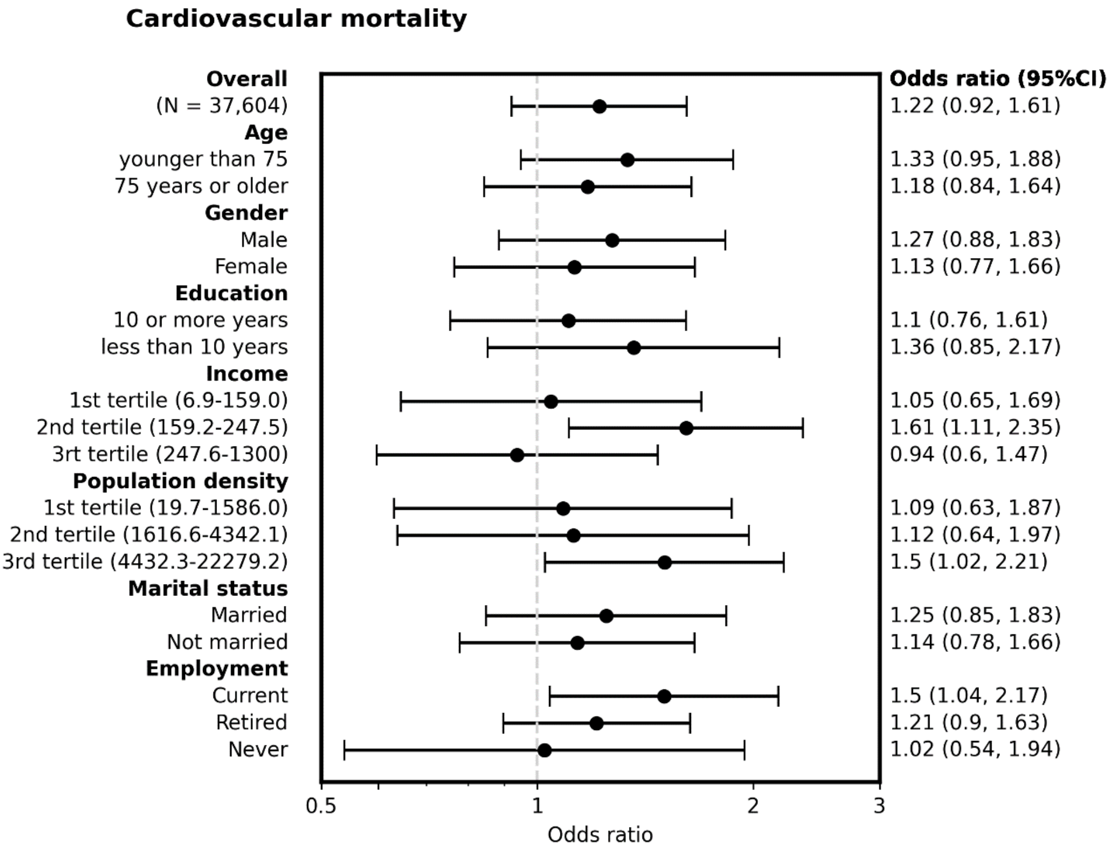


© 2024 Nakagomi A et al. *JAMA Network Open.*

**eFigure 10.** Odds Ratios of Social Isolation for Neoplasm Mortality Within All Participants and Subgroups of Moderators: Clustered at Municipal Levels

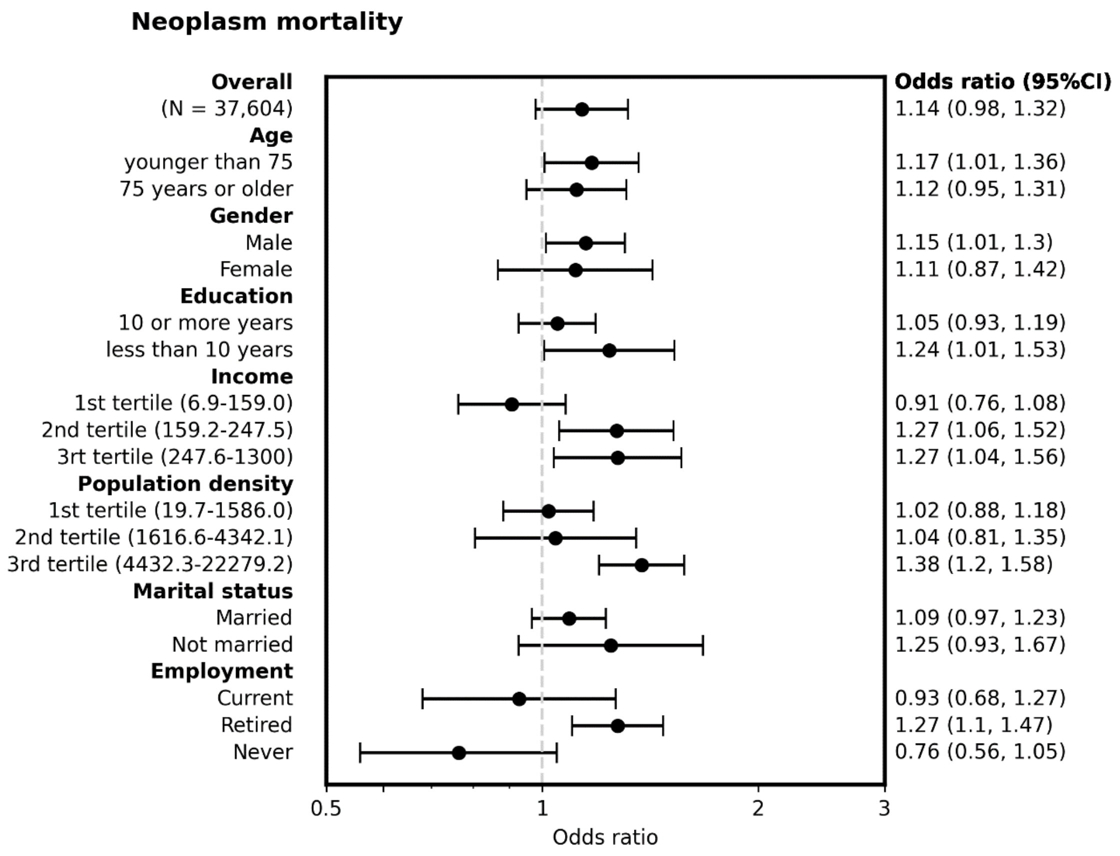


© 2024 Nakagomi A et al. *JAMA Network Open.*

**eFigure 11.** Hazard Ratios of Social Isolation for All-Cause Mortality, Cardiovascular Diseases Mortality, and Neoplasms Mortality With 6-Year Censoring

**All-cause mortality**

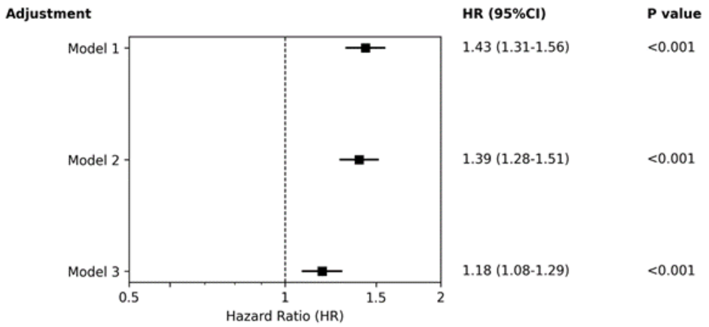


**Cardiovascular mortality**

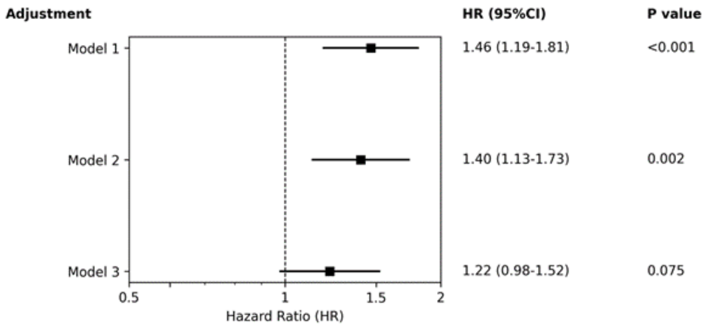


**Neoplasm mortality**

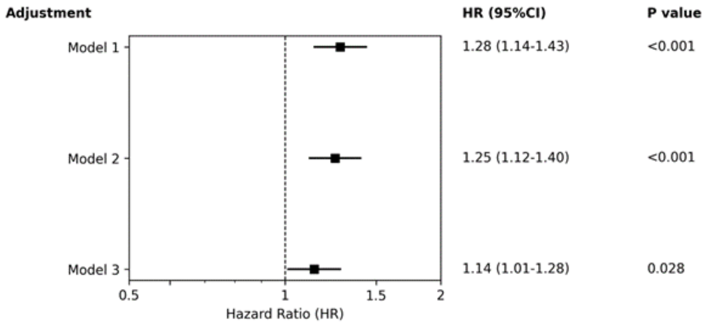


© 2024 Nakagomi A et al. *JAMA Network Open.*

In model 1, age and gender were included.

In model 2, socioeconomic status (education, equivalized income, population density), marital status, and employment status were included.

In model 3, health behaviours (smoking, drinking alcohol, walking, frequency of eating meat and fish, frequency of eating fruits and vegetables, taking health check-ups), psychological factors (depressive symptoms), and physiological factors (body mass index, self-rated health, hypertension, diabetes, dyslidemia) were included.

HR: hazard ratio; CI: confidence interval.

© 2024 Nakagomi A et al. *JAMA Network Open.*

**eFigure 12.** Hazard Ratios of Social Isolation for All-Cause Mortality Within All Participants and Subgroups of Moderators With 6-Year Censoring

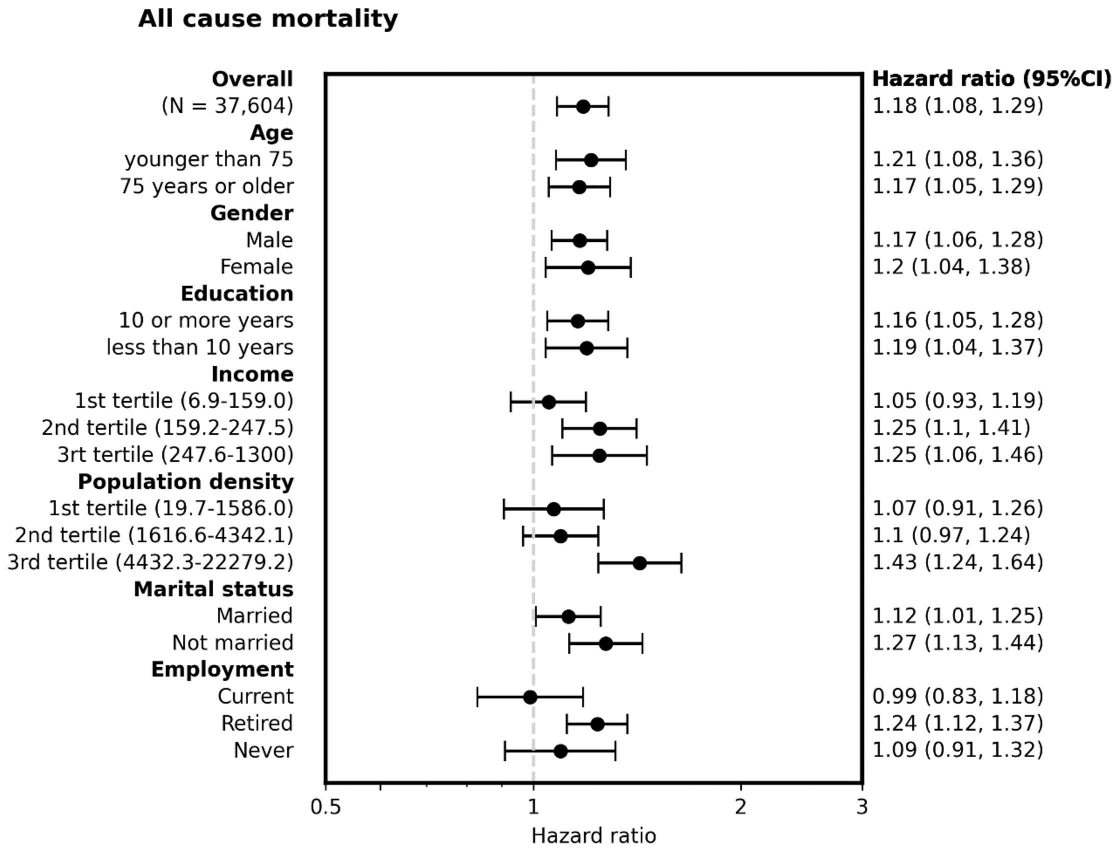


© 2024 Nakagomi A et al. *JAMA Network Open.*

**eFigure 13.** Hazard Ratios of Social Isolation for Cardiovascular Diseases Mortality Within All Participants and Subgroups of Moderators With 6-Year Censoring

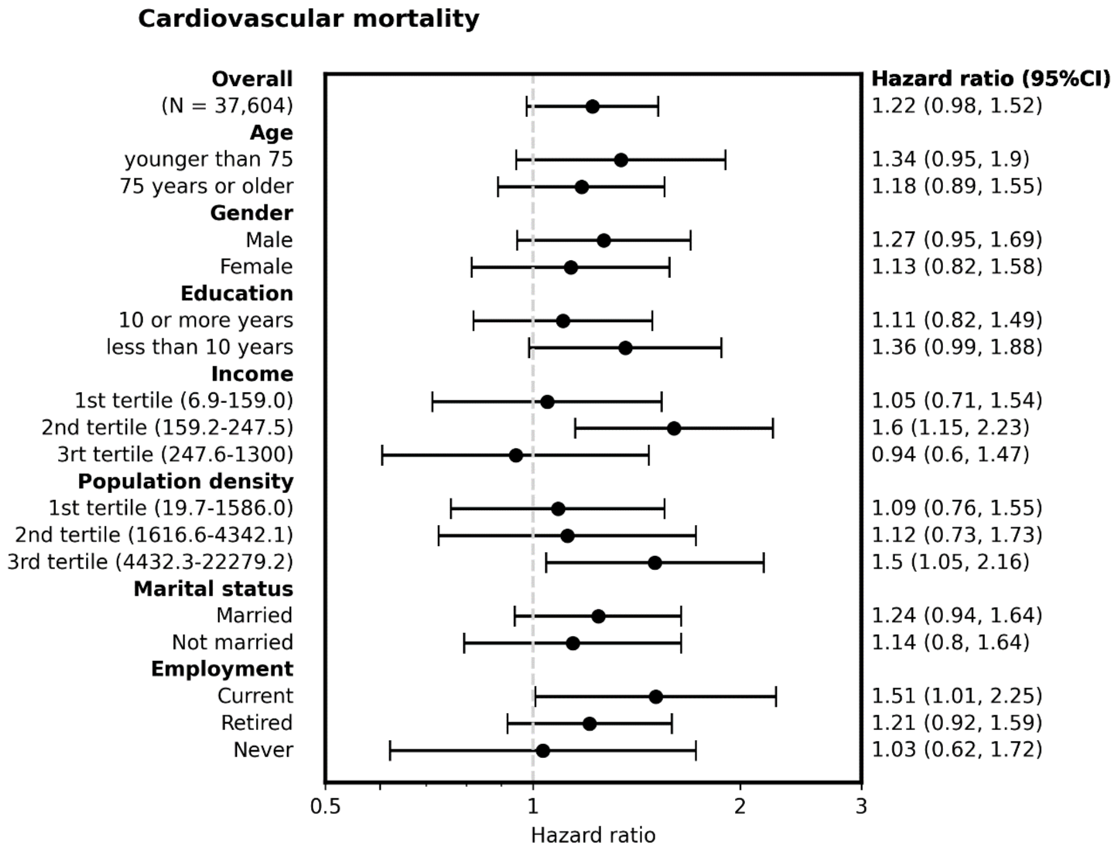


© 2024 Nakagomi A et al. *JAMA Network Open.*

**eFigure 14.** Hazard Ratios of Social Isolation for Neoplasms Mortality Within All Participants and Subgroups of Moderators With 6-Year Censoring

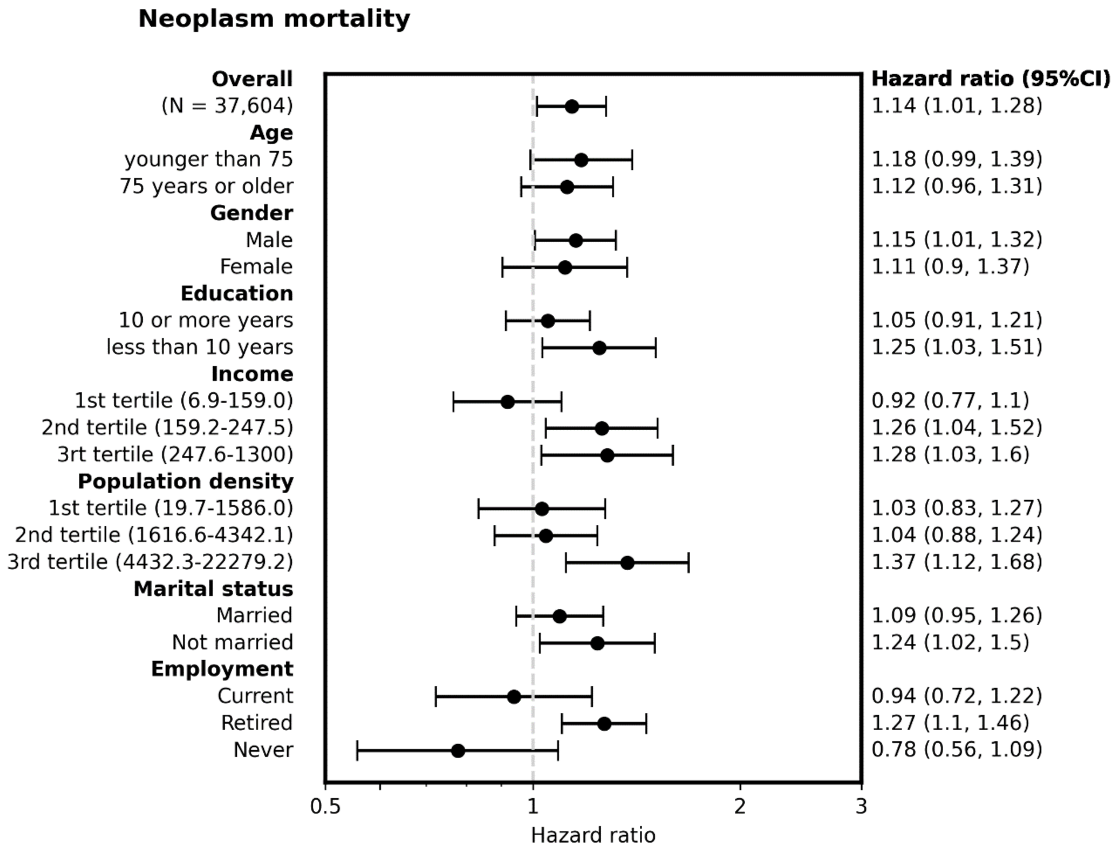


© 2024 Nakagomi A et al. *JAMA Network Open.*

**eFigure 15.** Hazard Ratios of Social Isolation for All-Cause Mortality Within All Participants and Subgroups of Moderators Without 6-Year Censoring

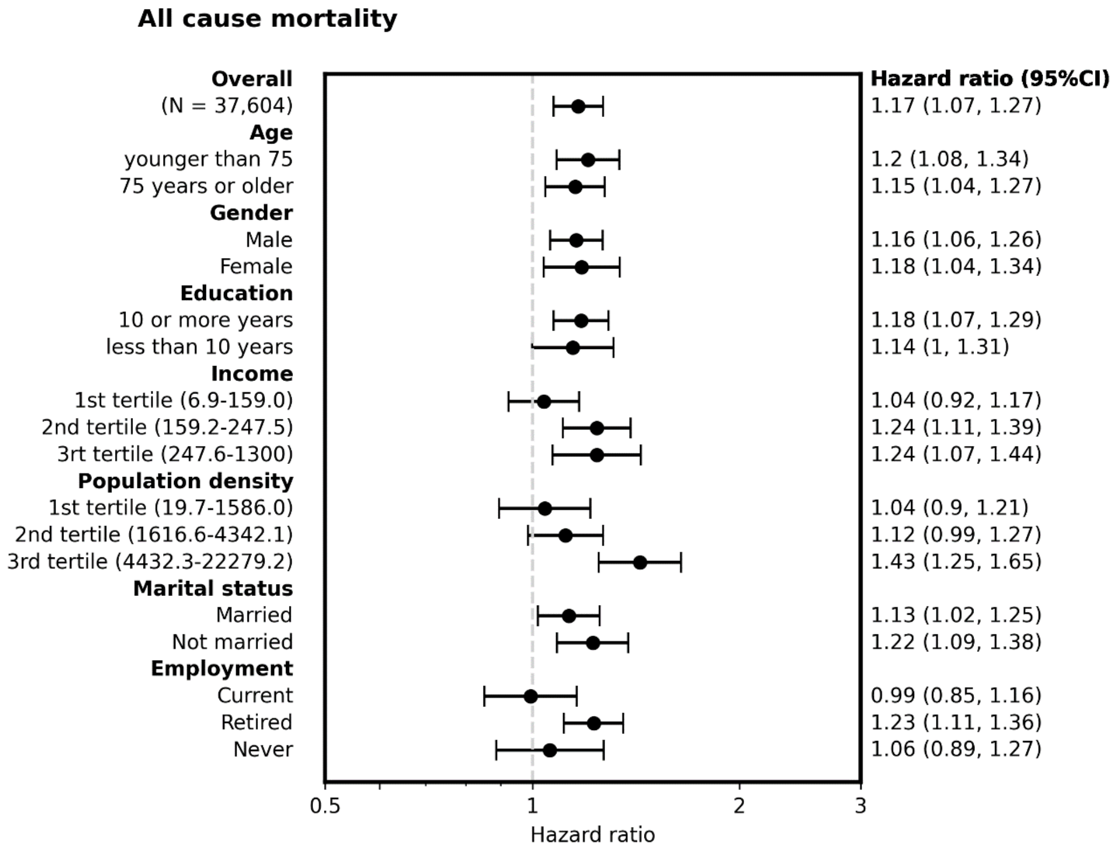


© 2024 Nakagomi A et al. *JAMA Network Open.*

**eFigure 16.** Hazard Ratios of Social Isolation for Cardiovascular Disease Mortality Within All Participants and Subgroups of Moderators Without 6-Year Censoring

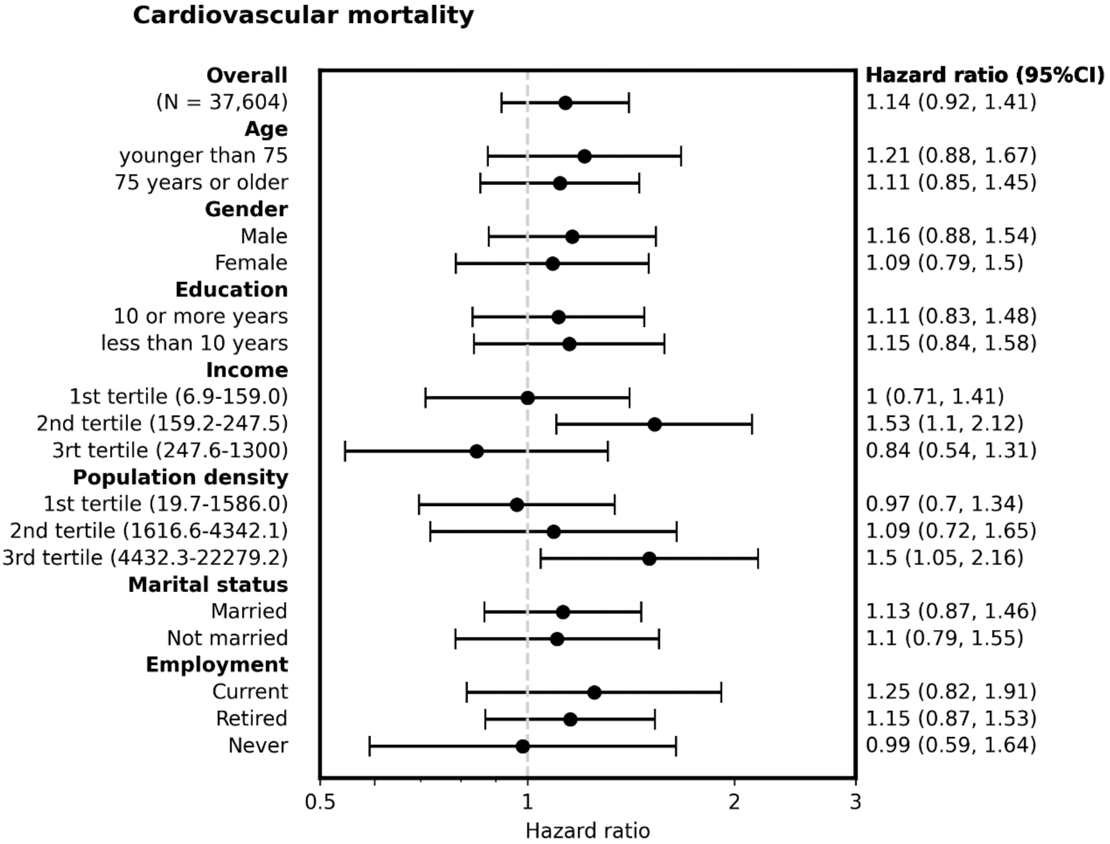


© 2024 Nakagomi A et al. *JAMA Network Open.*

**eFigure 17.** Hazard Ratios of Social Isolation for Neoplasm Mortality Within All Participants and Subgroups of Moderators Without 6-Year Censoring

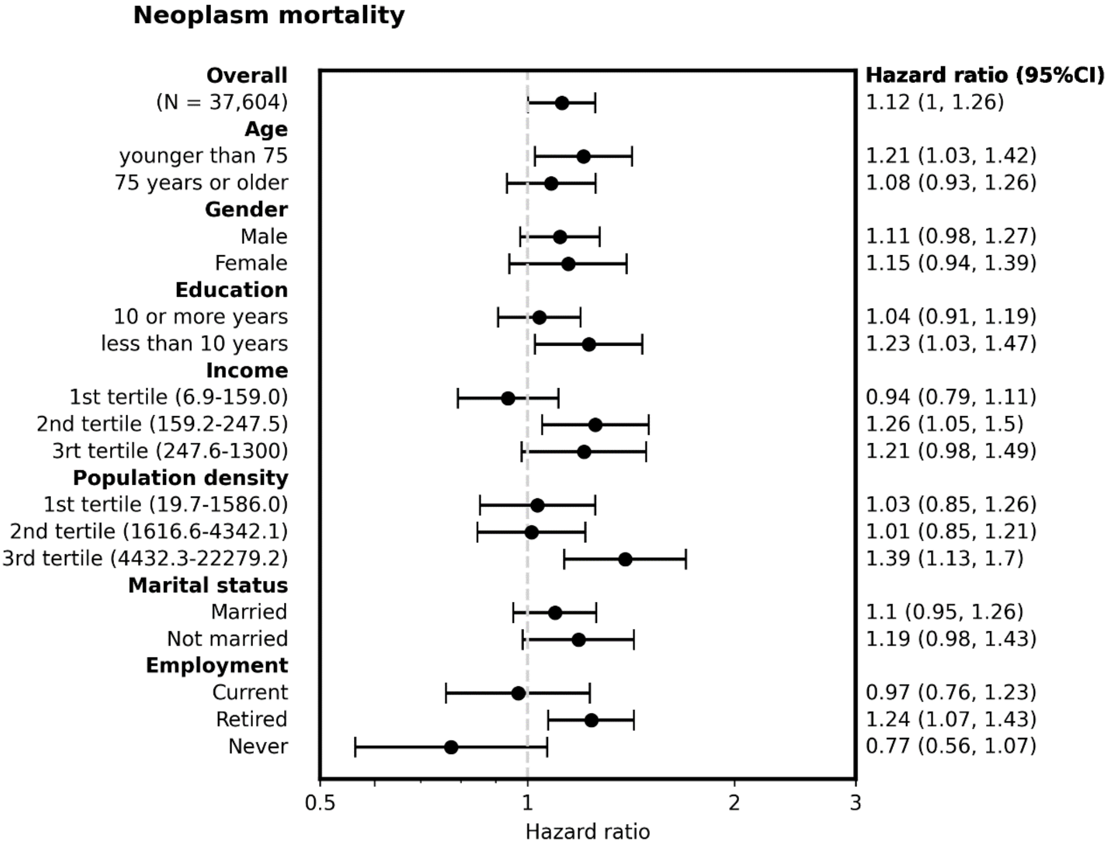


© 2024 Nakagomi A et al. *JAMA Network Open.*

**eFigure 18.** Kaplan-Meier Curves for All-Cause Mortality, Cardiovascular Disease Mortality, and Neoplasm Mortality by Social Isolation

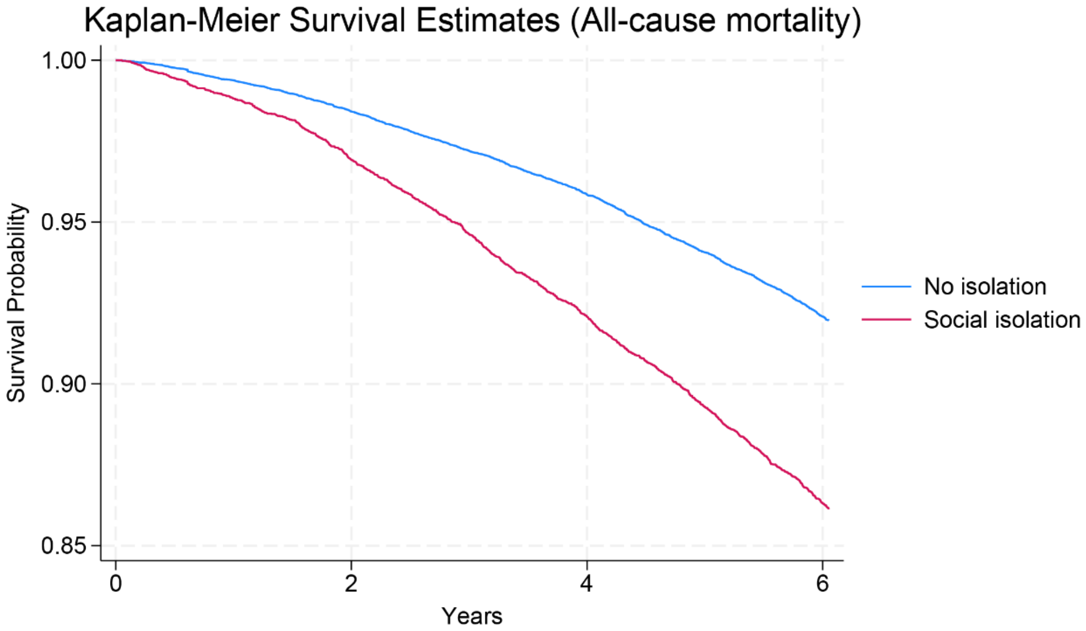


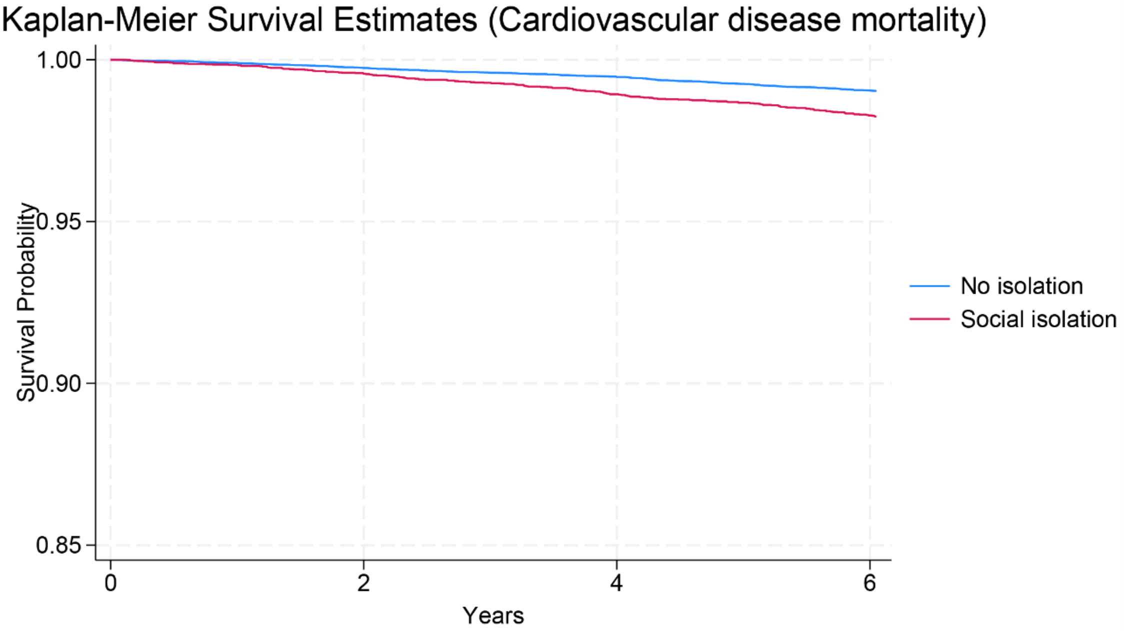


© 2024 Nakagomi A et al. *JAMA Network Open.*

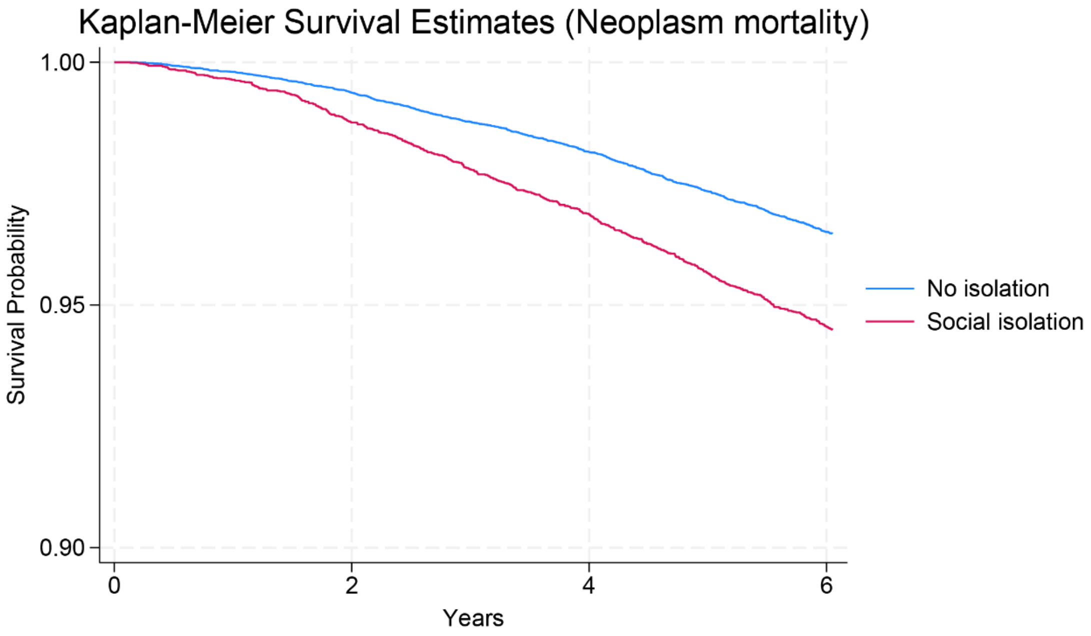


© 2024 Nakagomi A et al. *JAMA Network Open.*

**eFigure 19.** Cumulative Incidence Functions for Cardiovascular Disease Mortality and Neoplasm Mortality by Social Isolation

Cardiovascular disease mortality

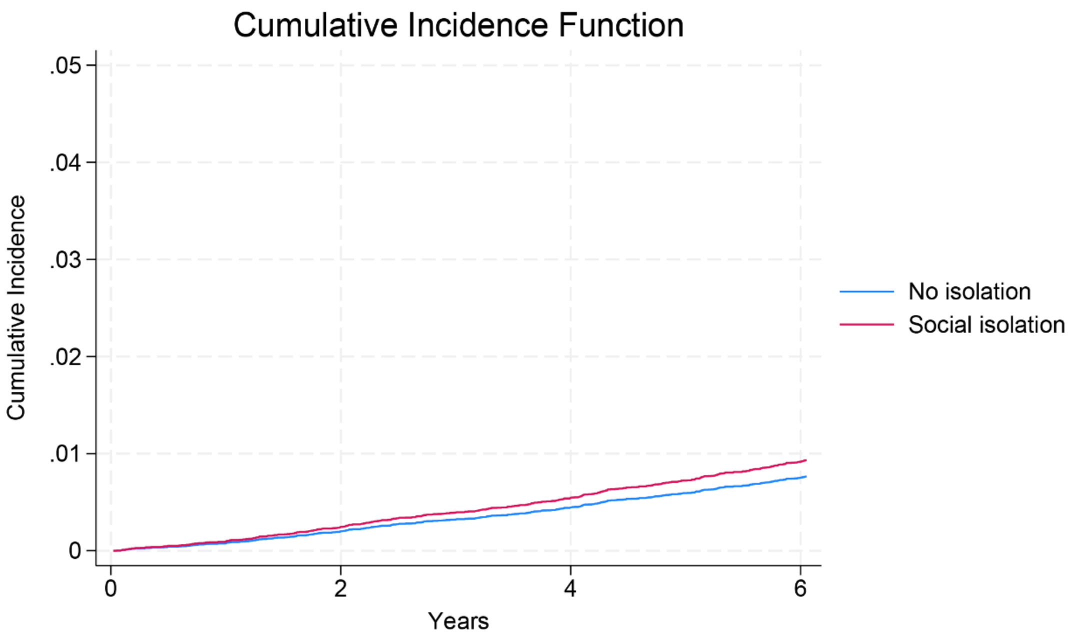


Neoplasm mortality

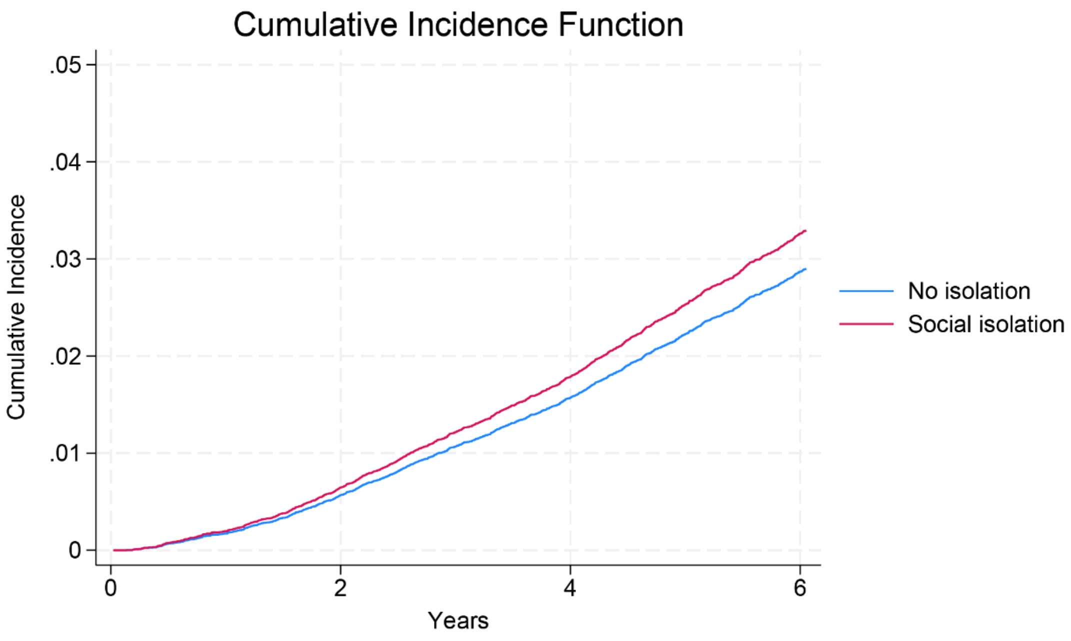


© 2024 Nakagomi A et al. *JAMA Network Open.*

**eTable 1.** Number (Percentage) of Missing Values

| **Varibles** | N (%) |
|---|---|
| Age | 0 (0%) |
| Gender | 0 (0%) |
| Education | 620 (1.7%) |
| Household equivalized income | 6457 (17.6%) |
| Population density | 0 (0%) |
| Marital status | 594 (1.6%) |
| Employment | 4392 (12%) |
| Smoking | 3456 (9.4%) |
| Drinking alcohol | 2091 (5.7%) |
| Walking | 2152 (5.9%) |
| Eating meat and fish every day | 2131 (5.8%) |
| Eating fruits and vegetables every day | 1895 (5.2%) |
| Taking health checks | 1473 (4%) |
| Geriatric Depression Scale | 6015 (16.4%) |
| Body mass index | 1419 (3.9%) |
| Self-rated health | 374 (1%) |
| Self reported hypertension | 10367 (28.3%) |
| Self reported diabetes | 10367 (28.3%) |
| Self reported dislipidemia | 10367 (28.3%) |
| Living alone | 569 (1.6%) |
| Meeting with friends | 2450 (6.7%) |
| Participation in sports groups | 7545 (20.6%) |
| Participation in hobby groups | 6444 (17.6%) |
| Participation in volunteer groups | 8988 (24.6%) |

© 2024 Nakagomi A et al. *JAMA Network Open.*

**eTable 2.** Baseline Characteristics (Obtained From the 2010 Survey) of Unimputed Data (N = 36 589)

| Baseline characteristics | Total | Social isolation | | |
|---|---|---|---|---|
| | | No | Yes | Missing |
| **Number of participants, n (%)** | 36,589 | 25,934 (70.9%) | 7,742 (21.2%) | 2,913 (8.0%) |
| Age, mean (SD) | 73.4 (5.9) | 73.2 (5.7) | 73.6 (6.3) | 75.5 (6.4) |
| Gender (Female), n (%) | 20382 (55.7%) | 15055 (58.1%) | 3614 (46.7%) | 1713 (58.8%) |
| Socioeconomic status | | | | |
| Education (10 years or more), n (%) | 19542 (54.3%) | 14244 (55.6%) | 4067 (53.1%) | 1231 (45.8%) |
| Household equivalized income (million JPY), mean (SD) | 239.1 (158.1) | 245.2 (159.7) | 223 (151) | 223.3 (158.4) |
| Population density (/km2), mean (SD) | 4213.2 (3932.9) | 4152.4 (3897.4) | 4381 (4015.4) | 4308.2 (4012.1) |
| Marital status (not married) | 10041 (27.9%) | 6118 (23.9%) | 3044 (39.7%) | 879 (32.5%) |
| Employment | | | | |
| Current | 7829 (24.3%) | 5560 (24.2%) | 1750 (24.6%) | 519 (24.7%) |
| Retired | 20250 (62.9%) | 14440 (62.8%) | 4513 (63.5%) | 1297 (61.6%) |
| Never | 4118 (12.8%) | 2983 (13%) | 846 (11.9%) | 289 (13.7%) |
| Behavioural factors | | | | |
| Smoking | | | | |
| Never, n (%) | 20253 (61.1%) | 14938 (63.4%) | 3803 (53.2%) | 1512 (63%) |
| Quit n (%) | 9002 (27.2%) | 6169 (26.2%) | 2211 (30.9%) | 622 (25.9%) |
| Current, n (%) | 3878 (11.7%) | 2471 (10.5%) | 1140 (15.9%) | 267 (11.1%) |

© 2024 Nakagomi A et al. *JAMA Network Open.*

| | | | | |
|---|---|---|---|---|
| Drinking alcohol | | | | |
| Never, n (%) | 12633 (36.6%) | 9079 (37%) | 2730 (37%) | 824 (31.6%) |
| Quit n (%) | 962 (2.8%) | 614 (2.5%) | 273 (3.7%) | 75 (2.9%) |
| Current, n (%) | 20903 (60.6%) | 14821 (60.5%) | 4372 (59.3%) | 1710 (65.5%) |
| Walking | | | | |
| <30min. , n (%) | 11012 (32%) | 7454 (29.7%) | 2847 (37.9%) | 711 (39.4%) |
| 30-59 min. , n (%) | 12227 (35.5%) | 9116 (36.3%) | 2552 (34%) | 559 (31%) |
| 60-89 min. , n (%) | 5576 (16.2%) | 4269 (17%) | 1045 (13.9%) | 262 (14.5%) |
| ≥90min. , n (%) | 5622 (16.3%) | 4293 (17.1%) | 1058 (14.1%) | 271 (15%) |
| Eating meat and fish every day | 14659 (42.5%) | 10802 (44.1%) | 2788 (38%) | 1069 (40.9%) |
| Eating fruits and vegetables every day | 27802 (80.1%) | 20234 (82.1%) | 5509 (74.6%) | 2059 (77.3%) |
| Taking health checks | | | | |
| Never, n (%) | 5640 (16.1%) | 3482 (13.9%) | 1581 (21.1%) | 577 (21.9%) |
| More than 4 years ago, n (%) | 3615 (10.3%) | 2447 (9.8%) | 917 (12.3%) | 251 (9.5%) |
| 2-3 years ago, n (%) | 4227 (12%) | 2983 (11.9%) | 934 (12.5%) | 310 (11.8%) |
| Within 1 year, n (%) | 21634 (61.6%) | 16090 (64.4%) | 4044 (54.1%) | 1500 (56.9%) |
| Psychological factors | | | | |
| Geriatric Depression Scale ≥ 5 | 7944 (26%) | 4892 (22.3%) | 2473 (37.1%) | 579 (28.7%) |
| Physiological factors | | | | |
| Body mass index | | | | |

© 2024 Nakagomi A et al. *JAMA Network Open.*

| | | | | |
|---|---|---|---|---|
| <18.5kg/m$^2$, n (%) | 2495 (6.8%) | 1584 (6.1%) | 685 (8.8%) | 226 (7.8%) |
| 18.5-25kg/m$^2$, n (%) | 25198 (68.9%) | 18126 (69.9%) | 5213 (67.3%) | 1859 (63.8%) |
| 25-30kg/m$^2$, n (%) | 6799 (18.6%) | 4936 (19%) | 1403 (18.1%) | 460 (15.8%) |
| ≥30kg/m$^2$, n (%) | 678 (1.9%) | 460 (1.8%) | 149 (1.9%) | 69 (2.4%) |
| Self-rated health (1=poor, 4=excellent), mean (SD) | 3 (0.6) | 3 (0.6) | 2.9 (0.6) | 2.9 (0.6) |
| Self reported hypertension, n (%) | 14105 (53.8%) | 10105 (54.4%) | 2886 (52.3%) | 1114 (51.9%) |
| Self reported diabetes, n (%) | 4230 (16.1%) | 2925 (15.8%) | 970 (17.6%) | 335 (15.6%) |
| Self reported dislipidemia, n (%) | 3591 (13.7%) | 2626 (14.1%) | 719 (13%) | 246 (11.5%) |

© 2024 Nakagomi A et al. *JAMA Network Open.*

**eTable 3.** Additive and Multiplicative Estimates of Effect Modification for the Associations Between Social Isolation and All-Cause Mortality Based on Logistic Regression

| | Odds ratio | Additive (RERI) | | Multiplicative (ROR) | |
|---|---|---|---|---|---|
| Moderator level | Estimate (95%CI) | Estimate (95%CI) | p value[a] | Estimate (95%CI) | p value[a] |
| Age | | | | | |
| Younger than 75 | 1.21 (1.07, 1.36) | Ref. | | Ref. | |
| 75 years or older | 1.19 (1.05, 1.34) | -0.1 (-0.28, 0.07) | 0.31 | 0.92 (0.79, 1.06) | 0.34 |
| Gender | | | | | |
| Male | 1.19 (1.07, 1.32) | Ref. | | Ref. | |
| Female | 1.21 (1.04, 1.42) | -0.09 (-0.22, 0.05) | 0.31 | 1.01 (0.86, 1.19) | 0.87 |
| Education | | | | | |
| 10 or more years | 1.18 (1.05, 1.33) | Ref. | | Ref. | |
| less than 10 years | 1.21 (1.04, 1.4) | 0.07 (-0.11, 0.24) | 0.44 | 1.08 (0.91, 1.27) | 0.48 |
| Income (10 thousand Japanese yen) | | | | | |
| Tertile 1 (6.9-159.0) | 1.05 (0.91, 1.2) | Ref. | | Ref. | |
| Tertile 2 (159.2-247.5) | 1.29 (1.12, 1.49) | 0.22 (0.03, 0.42) | 0.07 | 1.19 (1, 1.43) | 0.13 |
| Tertile 3 (247.6-1300) | 1.27 (1.06, 1.53) | 0.26 (0.04, 0.49) | 0.07 | 1.25 (1.01, 1.53) | 0.12 |
| Population density (/km2) | | | | | |
| Tertile 1 (19.7-1586.0) | 1.07 (0.88, 1.29) | Ref. | | Ref. | |
| Tertile 2 (1616.6-4342.1) | 1.1 (0.95, 1.27) | 0.11 (-0.15, 0.37) | 0.44 | 1.1 (0.87, 1.39) | 0.48 |
| Tertile 3 (4432.3-22279.2) | 1.47 (1.26, 1.72) | 0.34 (0.1, 0.58) | 0.03 | 1.45 (1.16, 1.82) | 0.01 |
| Marital status | | | | | |
| Married | 1.13 (1.01, 1.28) | Ref. | | Ref. | |
| Not married | 1.33 (1.15, 1.53) | 0.21 (0.01, 0.4) | 0.08 | 1.15 (0.98, 1.35) | 0.16 |
| Employment | | | | | |
| Current | 0.98 (0.81, 1.18) | -0.32 (-0.54, -0.11) | 0.03 | 0.75 (0.62, 0.92) | 0.03 |
| Retired | 1.27 (1.14, 1.43) | Ref. | | Ref. | |
| Never | 1.09 (0.89, 1.35) | -0.15 (-0.4, 0.1) | 0.31 | 0.87 (0.71, 1.07) | 0.30 |

© 2024 Nakagomi A et al. *JAMA Network Open.*

CI: confidence interval; RERI: relative excess risk due to interaction (additive interaction estimates); ROR: ratio of odds ratio (multiplicative interaction estimates).

[a]P values are corrected with Benjamini–Hochberg procedure.

© 2024 Nakagomi A et al. *JAMA Network Open.*

**eTable 4.** Additive and Multiplicative Estimates of Effect Modification for the Associations Between Social Isolation and Cardiovascular Diseases Mortality Based on Logistic Regression

| Moderator level | Odds ratio | Additive (RERI) | | Multiplicative (ROR) | |
|---|---|---|---|---|---|
| | Estimate (95%CI) | Estimate (95%CI) | p value[a] | Estimate (95%CI) | p value[a] |
| Age | | | | | |
| Younger than 75 | 1.33 (0.94, 1.89) | Ref. | | Ref. | |
| 75 years or older | 1.18 (0.89, 1.56) | -0.52 (-1.15, 0.11) | 0.51 | 0.66 (0.44, 0.99) | 0.23 |
| Gender | | | | | |
| Male | 1.27 (0.95, 1.7) | Ref. | | Ref. | |
| Female | 1.13 (0.8, 1.58) | -0.08 (-0.51, 0.35) | 0.89 | 0.98 (0.64, 1.49) | 0.92 |
| Education | | | | | |
| 10 or more years | 1.1 (0.81, 1.5) | Ref. | | Ref. | |
| less than 10 years | 1.36 (0.99, 1.88) | 0.3 (-0.11, 0.72) | 0.51 | 1.39 (0.9, 2.12) | 0.34 |
| Income (10 thousand Japanese yen) | | | | | |
| Tertile 1 (6.9-159.0) | 1.05 (0.71, 1.54) | Ref. | | Ref. | |
| Tertile 2 (159.2-247.5) | 1.61 (1.15, 2.25) | 0.56 (0.09, 1.03) | 0.19 | 1.71 (1.09, 2.69) | 0.20 |
| Tertile 3 (247.6-1300) | 0.94 (0.6, 1.47) | 0.07 (-0.45, 0.58) | 0.89 | 1.1 (0.61, 2) | 0.84 |
| Population density (/km2) | | | | | |
| Tertile 1 (19.7-1586.0) | 1.09 (0.76, 1.56) | Ref. | | Ref. | |
| Tertile 2 (1616.6-4342.1) | 1.12 (0.73, 1.73) | 0.17 (-0.47, 0.81) | 0.87 | 1.12 (0.67, 1.87) | 0.84 |
| Tertile 3 (4432.3-22279.2) | 1.5 (1.04, 2.17) | 0.38 (-0.23, 1) | 0.56 | 1.47 (0.92, 2.35) | 0.34 |
| Marital status | | | | | |
| Married | 1.25 (0.94, 1.65) | Ref. | | Ref. | |
| Not married | 1.14 (0.79, 1.64) | -0.04 (-0.61, 0.53) | 0.89 | 0.93 (0.61, 1.42) | 0.84 |
| Employment | | | | | |
| Current | 1.5 (1, 2.26) | 0.2 (-0.41, 0.8) | 0.87 | 1.15 (0.71, 1.87) | 0.84 |
| Retired | 1.21 (0.92, 1.6) | Ref. | | Ref. | |
| Never | 1.02 (0.61, 1.72) | -0.29 (-0.96, 0.39) | 0.81 | 0.78 (0.45, 1.33) | 0.71 |

© 2024 Nakagomi A et al. *JAMA Network Open.*

CI: confidence interval; RERI: relative excess risk due to interaction (additive interaction estimates); ROR: ratio of odds ratio (multiplicative interaction estimates).

[a]P values are corrected with Benjamini–Hochberg procedure.

© 2024 Nakagomi A et al. *JAMA Network Open.*

**eTable 5.** Additive and Multiplicative Estimates of Effect Modification for the Associations Between Social Isolation and Neoplasms Mortality Based on Logistic Regression

| Moderator level | Odds ratio | Additive (RERI) | | Multiplicative (ROR) | |
|---|---|---|---|---|---|
| | Estimate (95%CI) | Estimate (95%CI) | p value[a] | Estimate (95%CI) | p value[a] |
| Age | | | | | |
| Younger than 75 | 1.17 (0.99, 1.39) | Ref. | | Ref. | |
| 75 years or older | 1.12 (0.95, 1.31) | -0.06 (-0.33, 0.2) | 0.64 | 0.93 (0.75, 1.16) | 0.67 |
| Gender | | | | | |
| Male | 1.15 (1, 1.32) | Ref. | | Ref. | |
| Female | 1.11 (0.9, 1.37) | -0.08 (-0.26, 0.1) | 0.53 | 0.98 (0.78, 1.22) | 0.83 |
| Education | | | | | |
| 10 or more years | 1.05 (0.91, 1.21) | Ref. | | Ref. | |
| less than 10 years | 1.24 (1.02, 1.51) | 0.17 (-0.05, 0.4) | 0.22 | 1.2 (0.95, 1.52) | 0.21 |
| Income (10 thousands Japanese yen) | | | | | |
| Tertile 1 (6.9-159.0) | 0.91 (0.75, 1.09) | Ref. | | Ref. | |
| Tertile 2 (159.2-247.5) | 1.27 (1.05, 1.53) | 0.31 (0.08, 0.54) | 0.04 | 1.37 (1.07, 1.76) | 0.06 |
| Tertile 3 (247.6-1300) | 1.27 (1.02, 1.6) | 0.35 (0.1, 0.61) | 0.04 | 1.44 (1.09, 1.9) | 0.06 |
| Population density (/km2) | | | | | |
| Tertile 1 (19.7-1586.0) | 1.02 (0.82, 1.27) | Ref. | | Ref. | |
| Tertile 2 (1616.6-4342.1) | 1.04 (0.88, 1.24) | 0.07 (-0.23, 0.38) | 0.64 | 1.06 (0.8, 1.4) | 0.78 |
| Tertile 3 (4432.3-22279.2) | 1.38 (1.11, 1.7) | 0.22 (-0.06, 0.49) | 0.22 | 1.35 (1, 1.81) | 0.10 |
| Marital status | | | | | |
| Married | 1.09 (0.94, 1.27) | Ref. | | Ref. | |
| Not married | 1.25 (1.02, 1.52) | 0.11 (-0.16, 0.39) | 0.53 | 1.08 (0.86, 1.37) | 0.67 |
| Employment | | | | | |
| Current | 0.93 (0.71, 1.21) | -0.37 (-0.68, -0.07) | 0.05 | 0.72 (0.55, 0.95) | 0.06 |
| Retired | 1.27 (1.1, 1.48) | Ref. | | Ref. | |
| Never | 0.76 (0.54, 1.09) | -0.41 (-0.76, -0.06) | 0.05 | 0.67 (0.48, 0.95) | 0.06 |

© 2024 Nakagomi A et al. *JAMA Network Open.*

CI: confidence interval; RERI: relative excess risk due to interaction (additive interaction estimates); ROR: ratio of odds ratio (multiplicative interaction estimates).

[a]P values are corrected with Benjamini–Hochberg procedure.

© 2024 Nakagomi A et al. *JAMA Network Open.*

**eTable 6.** Robustness to Unmeasured Confounding (E-Values) of Associations Between Social Isolation and All-Cause Mortality, Cardiovascular Diseases Mortality, and Neoplasms Mortality

| | All-cause mortality | | CVD mortality | | Neoplasms mortality | |
|---|---|---|---|---|---|---|
| Moderator level | Effect estimate | CI limit | Effect estimate | CI limit | Effect estimate | CI limit |
| Age | | | | | | |
| Younger than 75 | 1.71 | 1.35 | 2.00 | 1.00 | 1.62 | 1.00 |
| 75 years or older | 1.65 | 1.29 | 1.63 | 1.00 | 1.48 | 1.00 |
| Gender | | | | | | |
| Male | 1.66 | 1.34 | 1.86 | 1.00 | 1.56 | 1.00 |
| Female | 1.72 | 1.25 | 1.50 | 1.00 | 1.46 | 1.00 |
| Education | | | | | | |
| 10 or more years | 1.65 | 1.29 | 1.44 | 1.00 | 1.28 | 1.00 |
| less than 10 years | 1.71 | 1.25 | 2.06 | 1.00 | 1.79 | 1.17 |
| Income (10 thousands Japanese yen) | | | | | | |
| Tertile 1 (6.9-159.0) | 1.26 | 1.00 | 1.26 | 1.00 | 1.44 | 1.00 |
| Tertile 2 (159.2-247.5) | 1.91 | 1.49 | 2.60 | 1.58 | 1.85 | 1.28 |
| Tertile 3 (247.6-1300) | 1.87 | 1.32 | 1.33 | 1.00 | 1.86 | 1.14 |
| Population density (/km2) | | | | | | |
| Tertile 1 (19.7-1586.0) | 1.34 | 1.00 | 1.39 | 1.00 | 1.16 | 1.00 |
| Tertile 2 (1616.6-4342.1) | 1.44 | 1.00 | 1.49 | 1.00 | 1.26 | 1.00 |
| Tertile 3 (4432.3-22279.2) | 2.31 | 1.84 | 2.37 | 1.26 | 2.10 | 1.47 |
| Marital status | | | | | | |
| Married | 1.52 | 1.10 | 1.80 | 1.00 | 1.40 | 1.00 |
| Not married | 1.98 | 1.58 | 1.53 | 1.00 | 1.80 | 1.18 |
| Employment | | | | | | |
| Current | 1.18 | 1.00 | 2.37 | 1.00 | 1.37 | 1.00 |
| Retired | 1.87 | 1.53 | 1.71 | 1.00 | 1.86 | 1.43 |

© 2024 Nakagomi A et al. *JAMA Network Open.*

| | | | | | | |
|---|---|---|---|---|---|---|
| Never | 1.42 | 1.00 | 1.18 | 1.00 | 1.94 | 1.00 |

Abbreviations: CI, confidence interval; CVD, cardiovascular diseases.

© 2024 Nakagomi A et al. *JAMA Network Open.*

**eTable 7.** Additive and Multiplicative Estimates of Effect Modification for the Associations Between Social Isolation and All-Cause Mortality Based on Survival Regression

| | Hazard ratio | Additive (RERI) | | Multiplicative (RHR) | |
|---|---|---|---|---|---|
| Moderator level | Estimate (95%CI) | Estimate (95%CI) | p value[a] | Estimate (95%CI) | p value[a] |
| Age | | | | | |
| Younger than 75 | 1.21 (1.08, 1.36) | Ref. | | Ref. | |
| 75 years or older | 1.17 (1.05, 1.29) | -0.15 (-0.32, 0.01) | 0.12 | 0.87 (0.77, 1) | 0.11 |
| Gender | | | | | |
| Male | 1.17 (1.06, 1.28) | Ref. | | Ref. | |
| Female | 1.2 (1.04, 1.38) | -0.04 (-0.16, 0.07) | 0.47 | 1.05 (0.91, 1.22) | 0.51 |
| Education | | | | | |
| 10 or more years | 1.16 (1.05, 1.28) | Ref. | | Ref. | |
| less than 10 years | 1.19 (1.04, 1.37) | 0.08 (-0.07, 0.24) | 0.40 | 1.09 (0.94, 1.26) | 0.35 |
| Income (10 thousands Japanese yen) | | | | | |
| Tertile 1 (6.9-159.0) | 1.05 (0.93, 1.19) | Ref. | | Ref. | |
| Tertile 2 (159.2-247.5) | 1.25 (1.1, 1.41) | 0.17 (0, 0.35) | 0.12 | 1.15 (0.98, 1.35) | 0.20 |
| Tertile 3 (247.6-1300) | 1.25 (1.06, 1.46) | 0.23 (0.04, 0.43) | 0.07 | 1.21 (1.01, 1.45) | 0.11 |
| Population density (/km2) | | | | | |
| Tertile 1 (19.7-1586.0) | 1.07 (0.91, 1.26) | Ref. | | Ref. | |
| Tertile 2 (1616.6-4342.1) | 1.1 (0.97, 1.24) | 0.09 (-0.14, 0.32) | 0.47 | 1.07 (0.87, 1.32) | 0.51 |
| Tertile 3 (4432.3-22279.2) | 1.43 (1.24, 1.64) | 0.32 (0.1, 0.53) | 0.03 | 1.4 (1.14, 1.71) | 0.01 |
| Marital status | | | | | |
| Married | 1.12 (1.01, 1.25) | Ref. | | Ref. | |
| Not married | 1.27 (1.13, 1.44) | 0.15 (-0.01, 0.32) | 0.12 | 1.11 (0.97, 1.28) | 0.24 |
| Employment | | | | | |
| Current | 0.99 (0.83, 1.18) | -0.27 (-0.46, -0.08) | 0.03 | 0.79 (0.66, 0.94) | 0.05 |
| Retired | 1.24 (1.12, 1.37) | Ref. | | Ref. | |
| Never | 1.09 (0.91, 1.32) | -0.09 (-0.31, 0.12) | 0.47 | 0.91 (0.76, 1.08) | 0.35 |

© 2024 Nakagomi A et al. *JAMA Network Open.*

CI: confidence interval; RERI: relative excess risk due to interaction (additive interaction estimates); RHR: ratio of hazard ratio (multiplicative interaction estimates).

[a]P values are corrected with Benjamini–Hochberg procedure.

© 2024 Nakagomi A et al. *JAMA Network Open.*

**eTable 8.** Additive and Multiplicative Estimates of Effect Modification for the Associations Between Social Isolation and Cardiovascular Diseases Mortality Based on Survival Regression

| Moderator level | Hazard ratio | Additive (RERI) | | Multiplicative (RHR) | |
|---|---|---|---|---|---|
| | Estimate (95%CI) | Estimate (95%CI) | p value[a] | Estimate (95%CI) | p value[a] |
| Age | | | | | |
| Younger than 75 | 1.34 (0.95, 1.9) | Ref. | | Ref. | |
| 75 years or older | 1.18 (0.89, 1.55) | -0.53 (-1.16, 0.09) | 0.47 | 0.65 (0.44, 0.98) | 0.19 |
| Gender | | | | | |
| Male | 1.27 (0.95, 1.69) | Ref. | | Ref. | |
| Female | 1.13 (0.82, 1.58) | -0.07 (-0.62, 0.49) | 0.91 | 0.99 (0.65, 1.5) | 0.96 |
| Education | | | | | |
| 10 or more years | 1.11 (0.82, 1.49) | Ref. | | Ref. | |
| less than 10 years | 1.36 (0.99, 1.88) | 0.3 (-0.11, 0.71) | 0.50 | 1.38 (0.91, 2.1) | 0.33 |
| Income (10 thousands Japanese yen) | | | | | |
| Tertile 1 (6.9-159.0) | 1.05 (0.71, 1.54) | Ref. | | Ref. | |
| Tertile 2 (159.2-247.5) | 1.6 (1.15, 2.23) | 0.55 (0.09, 1.02) | 0.20 | 1.71 (1.09, 2.67) | 0.19 |
| Tertile 3 (247.6-1300) | 0.94 (0.6, 1.47) | 0.06 (-0.45, 0.57) | 0.91 | 1.08 (0.6, 1.93) | 0.89 |
| Population density (/km2) | | | | | |
| Tertile 1 (19.7-1586.0) | 1.09 (0.76, 1.55) | Ref. | | Ref. | |
| Tertile 2 (1616.6-4342.1) | 1.12 (0.73, 1.73) | 0.16 (-0.47, 0.8) | 0.88 | 1.11 (0.67, 1.85) | 0.89 |
| Tertile 3 (4432.3-22279.2) | 1.5 (1.05, 2.16) | 0.39 (-0.23, 1.01) | 0.54 | 1.47 (0.92, 2.34) | 0.33 |
| Marital status | | | | | |
| Married | 1.24 (0.94, 1.64) | Ref. | | Ref. | |
| Not married | 1.14 (0.8, 1.64) | -0.03 (-0.59, 0.53) | 0.91 | 0.93 (0.61, 1.42) | 0.89 |
| Employment | | | | | |
| Current | 1.51 (1.01, 2.25) | 0.2 (-0.4, 0.79) | 0.87 | 1.17 (0.72, 1.88) | 0.88 |
| Retired | 1.21 (0.92, 1.59) | Ref. | | Ref. | |
| Never | 1.03 (0.62, 1.72) | -0.27 (-0.93, 0.39) | 0.85 | 0.79 (0.46, 1.33) | 0.75 |

© 2024 Nakagomi A et al. *JAMA Network Open.*

CI: confidence interval; RERI: relative excess risk due to interaction (additive interaction estimates); RHR: ratio of hazard ratio (multiplicative interaction estimates).

[a]P values are corrected with Benjamini–Hochberg procedure.

© 2024 Nakagomi A et al. *JAMA Network Open.*

**eTable 9.** Additive and Multiplicative Estimates of Effect Modification for the Associations Between Social Isolation and Neoplasms Mortality Based on Survival Regression

| Moderator level | Hazard ratio | Additive (RERI) | | Multiplicative (RHR) | |
|---|---|---|---|---|---|
| | Estimate (95%CI) | Estimate (95%CI) | p value[a] | Estimate (95%CI) | p value[a] |
| Age | | | | | |
| Younger than 75 | 1.18 (0.99, 1.39) | Ref. | | Ref. | |
| 75 years or older | 1.12 (0.96, 1.31) | -0.07 (-0.33, 0.19) | 0.66 | 0.93 (0.75, 1.15) | 0.67 |
| Gender | | | | | |
| Male | 1.15 (1.01, 1.32) | Ref. | | Ref. | |
| Female | 1.11 (0.9, 1.37) | -0.08 (-0.35, 0.2) | 0.66 | 0.98 (0.8, 1.22) | 0.89 |
| Education | | | | | |
| 10 or more years | 1.05 (0.91, 1.21) | Ref. | | Ref. | |
| less than 10 years | 1.25 (1.03, 1.51) | 0.18 (-0.04, 0.4) | 0.21 | 1.21 (0.96, 1.52) | 0.18 |
| Income (10 thousands Japanese yen) | | | | | |
| Tertile 1 (6.9-159.0) | 0.92 (0.77, 1.1) | Ref. | | Ref. | |
| Tertile 2 (159.2-247.5) | 1.26 (1.04, 1.52) | 0.29 (0.06, 0.52) | 0.05 | 1.35 (1.06, 1.72) | 0.07 |
| Tertile 3 (247.6-1300) | 1.28 (1.03, 1.6) | 0.34 (0.1, 0.59) | 0.05 | 1.44 (1.1, 1.88) | 0.07 |
| Population density (/km2) | | | | | |
| Tertile 1 (19.7-1586.0) | 1.03 (0.83, 1.27) | Ref. | | Ref. | |
| Tertile 2 (1616.6-4342.1) | 1.04 (0.88, 1.24) | 0.06 (-0.24, 0.37) | 0.68 | 1.05 (0.8, 1.38) | 0.80 |
| Tertile 3 (4432.3-22279.2) | 1.37 (1.12, 1.68) | 0.21 (-0.06, 0.48) | 0.21 | 1.33 (1, 1.77) | 0.11 |
| Marital status | | | | | |
| Married | 1.09 (0.95, 1.26) | Ref. | | Ref. | |
| Not married | 1.24 (1.02, 1.5) | 0.11 (-0.16, 0.38) | 0.63 | 1.08 (0.86, 1.35) | 0.67 |
| Employment | | | | | |
| Current | 0.94 (0.72, 1.22) | -0.36 (-0.65, -0.06) | 0.05 | 0.72 (0.55, 0.95) | 0.07 |
| Retired | 1.27 (1.1, 1.46) | Ref. | | Ref. | |
| Never | 0.78 (0.56, 1.09) | -0.4 (-0.73, -0.07) | 0.05 | 0.69 (0.49, 0.96) | 0.07 |

© 2024 Nakagomi A et al. *JAMA Network Open.*

CI: confidence interval; RERI: relative excess risk due to interaction (additive interaction estimates); RHR: ratio of hazard ratio (multiplicative interaction estimates).

[a]P values are corrected with Benjamini–Hochberg procedure.

© 2024 Nakagomi A et al. *JAMA Network Open.*
