# Supplementary material for: Sociodemographic Heterogeneity in the Associations of Social Isolation With Mortality
Source: JAMA Netw Open. 2024 May 24;7(5):e2413132. doi: 10.1001/jamanetworkopen.2024.13132 (PMC11127126; doi:10.1001/jamanetworkopen.2024.13132)
Supplement: Supplement 2. — Data Sharing Statement [file jamanetwopen-e2413132-s002.pdf]

Data Sharing Statement

Nakagomi. Sociodemographic Heterogeneity in the Associations of Social Isolation With Mortality. *JAMA Netw Open*. Published May 24, 2024.
doi:10.1001/jamanetworkopen.2024.13132

Data
Data available: Yes
Data types: Deidentified participant data
How to access data: The dataset supporting the conclusions of this article is available upon reasonable request from the researchers admitted by the JAGES committee (dataadmin.ml@jages.net). All JAGES datasets have ethical or legal restrictions for public deposition because of the inclusion of sensitive information from human participants.
When available: With publication

Supporting Documents
Document types: None

Additional Information
Who can access the data: researchers whose proposed use of the data has been approved
Types of analyses: for a specified purpose
Mechanisms of data availability: after approval of a proposal
